# Association between bisphosphonate use and COVID-19 related outcomes

**DOI:** 10.7554/eLife.79548

**Published:** 2023-08-03

**Authors:** Jeffrey Thompson, Yidi Wang, Tobias Dreischulte, Olga Barreiro, Rodrigo J Gonzalez, Pavel Hanč, Colette Matysiak, Harold R Neely, Marietta Rottenkolber, Thomas Haskell, Stefan Endres, Ulrich H von Andrian

**Affiliations:** 1 Cerner Enviza Malvern United States; 2 Dept. of Immunology, Harvard Medical School Boston United States; 3 Institute of General Practice and Family Medicine, University Hospital of Ludwig Maximilians-University Munich Munich Germany; 4 Center of Integrated Protein Science Munich and Division of Clinical Pharmacology, University Hospital, LMU Munich, Germany Munich Germany; University Medical Center Utrecht Netherlands; Radboud University Medical Centre Netherlands

**Keywords:** Bisphosphonate, COVID-19, retrospective cohort study, Human

## Abstract

**Background:**

Although there are several efficacious vaccines against COVID-19, vaccination rates in many regions around the world remain insufficient to prevent continued high disease burden and emergence of viral variants. Repurposing of existing therapeutics that prevent or mitigate severe COVID-19 could help to address these challenges. The objective of this study was to determine whether prior use of bisphosphonates is associated with reduced incidence and/or severity of COVID-19.

**Methods:**

A retrospective cohort study utilizing payer-complete health insurance claims data from 8,239,790 patients with continuous medical and prescription insurance January 1, 2019 to June 30, 2020 was performed. The primary exposure of interest was use of any bisphosphonate from January 1, 2019 to February 29, 2020. Bisphosphonate users were identified as patients having at least one bisphosphonate claim during this period, who were then 1:1 propensity score-matched to bisphosphonate non-users by age, gender, insurance type, primary-care-provider visit in 2019, and comorbidity burden. Main outcomes of interest included: (a) any testing for SARS-CoV-2 infection; (b) COVID-19 diagnosis; and (c) hospitalization with a COVID-19 diagnosis between March 1, 2020 and June 30, 2020. Multiple sensitivity analyses were also performed to assess core study outcomes amongst more restrictive matches between BP users/non-users, as well as assessing the relationship between BP-use and other respiratory infections (pneumonia, acute bronchitis) both during the same study period as well as before the COVID outbreak.

**Results:**

A total of 7,906,603 patients for whom continuous medical and prescription insurance information was available were selected. A total of 450,366 bisphosphonate users were identified and 1:1 propensity score-matched to bisphosphonate non-users. Bisphosphonate users had lower odds ratios (OR) of testing for SARS-CoV-2 infection (OR = 0.22; 95%CI:0.21–0.23; p<0.001), COVID-19 diagnosis (OR = 0.23; 95%CI:0.22–0.24; p<0.001), and COVID-19-related hospitalization (OR = 0.26; 95%CI:0.24–0.29; p<0.001). Sensitivity analyses yielded results consistent with the primary analysis. Bisphosphonate-use was also associated with decreased odds of acute bronchitis (OR = 0.23; 95%CI:0.22–0.23; p<0.001) or pneumonia (OR = 0.32; 95%CI:0.31–0.34; p<0.001) in 2019, suggesting that bisphosphonates may protect against respiratory infections by a variety of pathogens, including but not limited to SARS-CoV-2.

**Conclusions:**

Prior bisphosphonate-use was associated with dramatically reduced odds of SARS-CoV-2 testing, COVID-19 diagnosis, and COVID-19-related hospitalizations. Prospective clinical trials will be required to establish a causal role for bisphosphonate-use in COVID-19-related outcomes.

**Funding:**

This study was supported by NIH grants, AR068383 and AI155865, a grant from MassCPR (to UHvA) and a CRI Irvington postdoctoral fellowship, CRI2453 (to PH).

## eLife digest

The COVID-19 pandemic challenged the world to rapidly develop strategies to combat the virus responsible for the disease. While several effective vaccines and new drugs have since become available, these therapies are not always easy to access and take time to generate and distribute. To address these challenges, researchers have tried to find ways to repurpose existing medications that are already commonly used and known to be safe.

One potential candidate are bisphosphonates, a family of drugs used to reduce bone loss in patients with osteoporosis. Bisphosphonates have been shown to boost the immune response to viral infections, and it has been observed that patients prescribed these drugs are less likely to develop or die from pneumonia. But whether bisphosphonates are effective against COVID-19 had not been fully explored.

To investigate, Thompson, Wang et al. analyzed insurance claims data from about 8 million patients between January 2019 and June 2020, including around 450,000 individuals that had filled a prescription for bisphosphonates. Patients prescribed bisphosphonates were then compared to non-users that were similar in terms of their gender, age, the type of health insurance they had, their access to healthcare, and other health comorbidities.

The study revealed that bisphosphonate users were around three to five times less likely to be tested for, diagnosed with, or hospitalized for COVID-19 during the first four months of the pandemic. They were also less commonly diagnosed with other respiratory infections in 2019, like bronchitis or pneumonia.

Although the results suggest that bisphosphonates provide some protection against COVID-19, they cannot directly prove it. Verifying that bisphosphonates can treat or prevent COVID-19 and/or other respiratory infections requires more studies that follow patients in real-time rather than studying previously collected data.

If such studies confirm the link, bisphosphonates could be a helpful tool to protect against COVID-19 or other virus outbreaks. The drugs are widely available, safe, and affordable, and therefore may provide an alternative for patients who cannot access other medications or vaccines.

## Introduction

Throughout the COVID-19 pandemic, massive global efforts to repurpose existing drugs as potential therapeutic options for COVID-19 have been undertaken. Drug repurposing, whereby a drug already proven to be safe and effective in humans for another approved clinical indication is evaluated for novel clinical use, may allow for faster identification and deployment of therapeutic agents compared to traditional drug discovery pipelines. Using in silico and in vitro analyses, a growing list of drugs have been suggested to be potentially efficacious in treating COVID-19 by either direct or indirect antiviral actions (Sultana et al., 2020). Another potentially beneficial class of drugs may be agents that boost or modulate anti-viral immune responses to SARS-CoV-2 infection to reduce clinical symptoms and/or mitigate disease progression. Regardless of the mechanism of action, ultimately, randomized prospective clinical studies are needed to test the safety and efficacy of each candidate in treating or preventing COVID-19. Observational studies can help prioritize candidates for prospective clinical testing, by examining associations between the use of a candidate drug and the incidence or severity of disease in users compared to a matched group of non-users. Drugs with strong observational evidence for potential effectiveness against COVID-19 may then be considered for prospective trials (Sultana et al., 2020).

Here, we have investigated bisphosphonates (BPs), a class of small-molecule drugs that inhibit bone resorption by osteoclasts (Roelofs et al., 2010b). BPs are widely prescribed as either oral or intravenous formulations to treat osteoporosis, Paget disease, and malignancy-induced hypercalcemia. Additionally, BPs are used as adjuvant therapy for breast cancer (Dhesy-Thind et al., 2017). BPs are subdivided into two classes, nitrogen-containing (amino-BPs) and nitrogen-free BPs (non-amino-BPs; Russell et al., 2008). Both accumulate in bone but have distinct molecular mechanisms by which they kill osteoclasts to prevent bone resorption (Roelofs et al., 2010b).

Aside from depleting osteoclasts, clinical and experimental studies indicate that BPs exert a plethora of immunomodulatory effects, providing a rationale for exploring BPs as potential repurposed drug candidates for COVID-19 (Brufsky et al., 2020). Indeed, amino-BPs regulate the activation, expansion, and/or function of a major subset of human γδT cells (Poccia et al., 2006; Hewitt et al., 2005; Tu et al., 2011) as well as neutrophils (Favot et al., 2013), monocytes (Roelofs et al., 2010a), and macrophages (Rogers and Holen, 2011; Wolf et al., 2006); they can modulate the antigen-presentation capacity of dendritic cells (Xia et al., 2018); and in animal studies, both amino-BPs and non-amino-BPs exerted potent adjuvant-like activity to boost antibody and T cells responses to viral antigens (Tonti et al., 2013). Furthermore, observational studies have reported decreased in-hospital mortality for patients in the ICU (Lee et al., 2016), and reduced incidence of pneumoniae and pneumonia-related mortality in patients treated with amino-BPs versus controls (Sing et al., 2020). These immunological and clinical effects of BPs combine with several other characteristics that make BPs well-suited as repurposed drug candidates in the context of a pandemic: they are globally accessible as generics, affordable, straightforward to administer, and have known safety profiles in adult (Suresh et al., 2014) and paediatric populations (Sbrocchi et al., 2010; George et al., 2015).

In light of these considerations, we have analysed a database of health insurance claims in the U.S. to determine if prior BP-use is associated with a differential incidence and/or severity of COVID-19-related outcomes. Specifically, we assessed the relationship between use of BPs and COVID-19-related hospitalizations and COVID-19 diagnosis, as well as testing for SARS-CoV-2 infection (as a proxy for severe COVID-19 symptoms given the restricted access to testing during the initial surge). Outcomes were measured from March 1, 2020 to June 30, 2020, a period that roughly coincided with the first wave of COVID-19 in the U.S. and predated the advent of potential outcome modifiers, such as vaccines or other effective treatment options.

## Methods

### Study design

**Figure 1.**
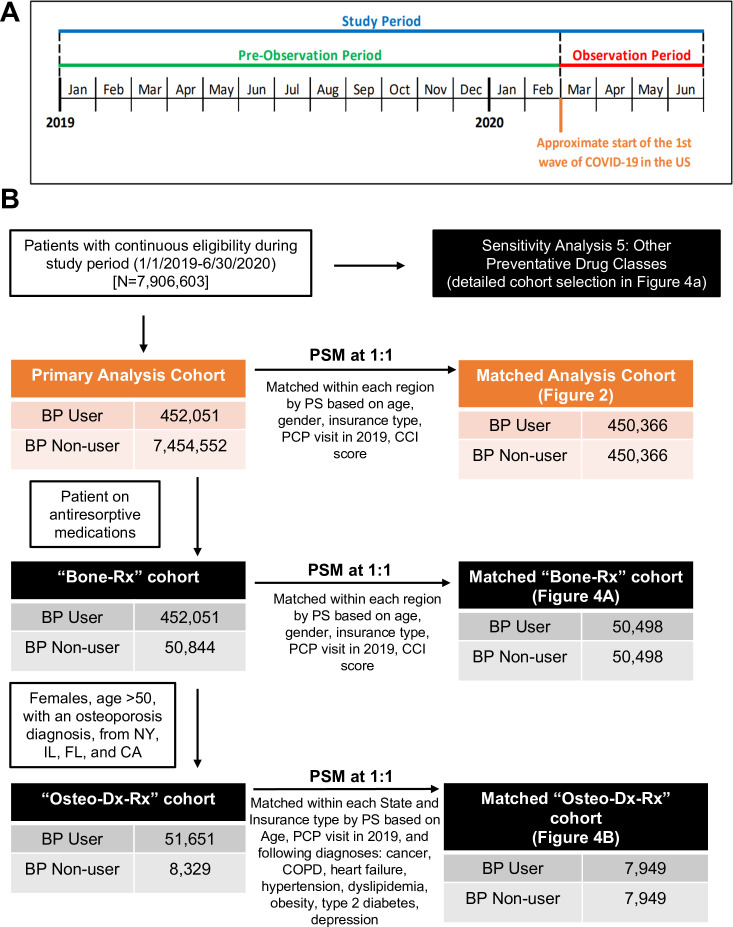
Study periods, cohort selection, and analyses of BP use on COVID-19-related outcomes. (**A**) Schematic overview of the study timeline. (**B**) Schematic flow diagram illustrating the identification of the study population and matched control populations for primary analysis and sensitivity analyses cohorts. *BP: bisphosphonate; CA: California; CCI: Charlson comorbidity index; CI: confidence interval; COPD: chronic obstructive pulmonary disease; FL: Florida; IL: Illinois; NY: New York; OR: odds ratio; PCP: primary care physician; PS: propensity score; PSM: propensity score match*.

A retrospective cohort study was performed using health insurance claims data from January 1, 2019 to June 30, 2020 (study period) in order to assess the relationship between use of BPs and three COVID-19-related outcomes: (a) testing for SARS-CoV-2 infection; (b) COVID-19 diagnosis; and (c) hospitalization with a COVID-19 diagnosis, whereby COVID-19-related hospitalization was deemed the primary endpoint and COVID-19 diagnosis and testing were secondary endpoints. Primary and secondary endpoints were assessed during the observation period of March 1, 2020 to June 30, 2020, roughly corresponding to the first nation-wide surge of COVID-19 in the U.S. (Figure 1A). In the primary analysis, the risk of COVID-19-related outcomes was assessed among BP users compared to a matched sample of BP non-users with similar demographic and clinical characteristics.

### Data source

Data used for this study included closed medical (inpatient and outpatient) and outpatient-pharmacy-dispensed claims between January 1, 2019 and June 30, 2020, from the Komodo Health payer-complete dataset (https://www.komodohealth.com). This dataset is derived from over 150 private insurers in the U.S. and includes patients with commercial, individual, state exchange-purchased, Medicare Advantage, and Medicaid managed-care insurance coverage. The dataset also provides information on insurance eligibility periods. Closed claims within this dataset represent those that had undergone insurance adjudication. In total, the Komodo Health payer-complete dataset includes health insurance claims data from over 140 million individuals in the U.S. from 2015 to 2020.

### Cohort definition

All patients were required to have continuous medical and prescription insurance eligibility during the entire study period. Patients with missing information for age, gender, insurance type, or state/region were excluded.

### Exposures of interest

The primary exposure of interest was the use of any amino- or non-amino BP medication. Exposure to BPs and all other medications of interest were assessed over a 14-month pre-observation period preceding the COVID-19 pandemic in the U.S. This long duration was chosen because of the extended bioavailability of BPs, which accumulate in bone where they are retained and slowly released for up to several years (Cremers et al., 2019). Patients were classified as BP users if they had any claim at any time during the pre-observation period for one of the following: alendronate, alendronic acid, etidronate, ibandronate, ibandronic acid, pamidronate, risedronate, and zoledronic acid (full details in **Appendix 1**).

### Timing of BP dose

The effect of timing and formulation of BPs on COVID-19-related outcomes was more closely examined by varying the window between BP exposure and outcome measurement. The primary analysis BP user cohort, along with their propensity-score matched (see below for cohort matching) BP non-user cohort, were stratified as follows: two cohorts were used as the reference comparator with known BP-exposure during all or most of the pre-observation and the entire observation period, specifically (i) BP users who took oral alendronic acid (dosed daily or weekly) throughout the pre-observation period (i.e. at least one claim or drug-on-hand in each quarter in 2019 and in Jan/Feb. 2020) that also had a days-supply extending past June 30, 2020, and (ii) users of infusion zoledronic acid (dosed annually) with a claim in Q3 or Q4 2019; two cohorts with BP-exposure only during the pre-observation period, namely (iii) users of alendronic acid occurring during the first six months of 2019 with days-covered ending prior to June 30, 2019 and no other BP claims thereafter, and (iv) users of zoledronic acid in January or February 2019 with no other BP claims during the remainder of the study period; and, two cohorts with short-term BP exposure, specifically new users of (v) alendronic acid or (vi) zoledronic acid in February 2020, with no prior BP claims during the pre-observation period.

### Covariates

As covariates, we considered factors that may influence either the use of BPs or potential modulators of primary or secondary study endpoints. These included: age; gender; insurance type (commercial, dual, Medicaid, Medicare); having had any primary care physician (PCP) visit in 2019; and comorbidity burden. The variable ‘PCP visit in 2019’ was used to control for prior healthcare-use behaviour and was assigned based on any physician office claim from January 1, 2019 to December 31, 2019 with one of the following provider types: family practice, general practice, geriatric medicine, internal medicine, and preventive medicine. Comorbidity score assignment was calculated following the Charlson Comorbidity Index (CCI) methodology (Quan et al., 2005), and was based on diagnosis codes present on any medical claim (inpatient or outpatient) occurring during the pre-observation period. The assigned CCI score was used as the comorbidity covariate for the primary cohort propensity score matching, but to better control for differences in comorbidity burden when assessing outcomes, all regression analyses involving the primary analysis cohort included the following individual comorbidity covariates in lieu of the aggregate CCI score: osteoporosis, cancer, chronic obstructive pulmonary disease (COPD), depression, dyslipidaemia, hypertension, obesity, type 2 diabetes, cardiovascular disease overall, sickle cell anemia, stroke, dementia, HIV/AIDS, chronic kidney disease/end-stage renal disease (CKD/ESRD), and liver disease (**Appendix 1**).

### Cohort matching

For the primary analysis, BP users were propensity-score (PS) matched to BP non-users via a PS calculated using multiple variables, including age, gender, insurance type, CCI, and any PCP visit in 2019, to yield comparable populations by demographics and clinical characteristics (Figure 1B). To account for the differential geographic spread of COVID-19 across the U.S. during the observation period, matching was performed within each geographic region separately (Northeast, Midwest, South, West) and then combined. In addition to this within-region stratified match, a cohort build was also performed after restricting to patients from New York (NY) state only, since this state was the site of the largest outbreak in the initial COVID-19 surge in the U.S. All matching algorithms used a greedy-match propensity score technique (Parsons, 2001) to match BP users to non-users with a maximum permitted propensity-score difference of 0.015.

### Definition of endpoints

Primary and secondary endpoints were assigned using inpatient and outpatient medical claims that occurred during the four-month observation period. The primary endpoint, COVID-19-related hospitalization, was assigned based on the presence of an International Classification of Diseases, Tenth Revision (ICD-10) code on any inpatient medical service claim indicating test-confirmed 2019 Novel Coronavirus (2019-nCoV) acute respiratory disease, specifically U07.1. The first secondary endpoint, SARS-CoV-2 testing, was assigned using Current Procedural Terminology (CPT) codes indicating a test for active infection, specifically 87635, 87636, and 87637. The second secondary endpoint, COVID-19-related diagnosis, was assigned based on any medical service claim with the ICD-10 diagnosis code U07.1.

### Statistical analysis

Unadjusted analyses assessing the association between BP-use and COVID-19-related outcomes were performed for the primary analysis cohort using chi-square tests for categorical variables and calculation of the crude unadjusted odds ratio (OR) in the matched cohort groups overall, when stratified by region and in NY state alone, and when further stratified by age group and gender. Chi-square tests for categorical variables and t-tests for continuous variables were also performed to assess differences in demographic and clinical characteristics of BP users compared to BP non-users both pre-match and post-match to assess the success of the propensity-score match.

Multivariate logistic regression analyses, modelled separately to determine the adjusted OR for each COVID-19-related primary and secondary outcome while adjusting for demographic and clinical characteristics, were performed on the matched primary analysis cohort with all regions combined, when stratified by region, and in NY state alone. The primary exposure of interest was BP-use (yes/no) during the pre-observation period. Additional demographic/clinical characteristics also included as regression model covariates were: age group, gender, region (for all regions-combined analyses), insurance type, PCP visit in 2019, and the following comorbid conditions: osteoporosis, cancer, COPD, depression, dyslipidaemia, hypertension, obesity, type 2 diabetes, cardiovascular disease overall, sickle cell anaemia, stroke, dementia, HIV/AIDS, CKD/ESRD, and liver disease. Demographic characteristics used in the matching procedure were also included in the final outcome regressions to control for the impact of those characteristics on outcomes modelled.

All tests were two-tailed, and p-values of less than 0.05 were considered significant. All analyses were performed using SAS 9.4 (Cary, NC).

### Sensitivity analyses

Multiple sensitivity analyses were performed to assess the reliability of the primary analysis results and/or to address potential unmeasured confounding (full details in **Appendix 1**).

1. The first sensitivity analysis addressed potential confounding by indication (i.e. the possibility of the indication for BP use rather than BP use itself being responsible for differences in outcomes among BP users and non-users) by restricting the control group to an active comparator cohort of patients who had used non-BP anti-resorptive bone medications during the pre-observation period. Users of non-BP anti-resorptive bone medications, the smaller patient population, were then 1:1 matched to BP users, providing a sample where all patients had used bone health medications during the pre-observation period (‘*Bone-Rx’* cohort) (Figure 1B). Cohort matching and regression modelling were performed following the same methodology employed for the primary analysis.
2. The second sensitivity analysis further addressed potential baseline differences between users of BPs and users of non-BP anti-resorptive bone medications in terms of indication for treatment and risk of SARS-CoV-2 exposure. To homogenise indication for treatment, we restricted the ‘Bone-Rx’ cohort to females aged older than 50 years with an osteoporosis diagnosis (ICD-10: M80.x, M81.x, M82.x), which is the main (but not the only) indication for use of anti-resorptive bone medications. In order to homogenise risk of COVID-19 exposure, we additionally (a) restricted both groups to residents of New York, Illinois, Florida, and California (four states with a high incidence of COVID-19 cases during the observation period, with each representing a geographic region) (CDC, 2021a), and (b) matched within each state by insurance-type strata (i.e. BP non-users matched to BP users with Medicaid coverage residing in New York) to control for differences in socioeconomic characteristics. Non-BP anti-resorptive bone medication users were then matched to BP users by age, PCP visit in 2019, and the following select comorbid conditions that include those thought to impact COVID-19 severity: cancer, COPD, depression, dyslipidaemia, heart failure, hypertension, obesity, and type 2 diabetes (Rosenthal et al., 2020). In addition to assessing COVID-19-related outcomes, the matched cohorts that resulted from this analysis, older female patients from New York, Illinois, Florida, or California with a diagnosis of osteoporosis who were users of BP or non-BP anti-resorptive medications (‘*Osteo-Dx-Rx’* cohort), were used for the third and fourth sensitivity analyses (see below).
3. The third sensitivity analysis assessed the relationship between BP-use and exploratory positive control outcomes (anticipated to be impacted by the immunomodulatory pharmacological mechanism of BPs) occurring in 2019. For this analysis, the primary, ‘*Bone-Rx’*, and *Osteo-Dx-Rx*” cohorts were restricted to BP users who had any BP claim during the first half of 2019 and their previously-assigned BP non-user matched pair to assess the relationship between BP-use and medical services for other respiratory infectious diseases (acute bronchitis, pneumonia).
4. The fourth sensitivity analysis addressed potential bias due to the 'healthy adherer' effect, whereby users of a preventive drug may have better disease outcomes due to their healthier behaviours rather than due to drug treatment itself (Ladova et al., 2014). Two strategies were employed to validate the findings from our primary analysis while controlling for the potential impact of healthy adherer effect-associated bias. First, we tested whether effects observed with exposure to BPs were similarly observed with exposure to other preventive drugs, namely statins, antihypertensives, antidiabetics, and antidepressants. Second, we assessed whether the association between BP-use and COVID-19-related outcomes was maintained among the matched user/non-user populations of these other preventive drugs, i.e. BP users were compared to BP non-users within, for example, the statin user population and separately within the matched statin non-user population.

## Results

### Study population

A total of 8,239,790 patients met the inclusion criterion of continuous medical and prescription insurance eligibility over the full study period, of which 333,107 were excluded due to missing demographic information, resulting in a total eligible sample of 7,906,603 patients (Figure 1B). Of this full population, 452,051 (5.7%) and 7,454,552 (94.3%) patients were classified as BP users and BP non-users, respectively. Within BP users, more than 99% were prescribed an amino-BP, with oral alendronic acid (75.4%), zoledronic acid infusion (11.5%), and oral ibandronic acid (8.4%) as the most prevalent formulations (Table 1).

**Table 1.** Most recent bisphosphonate claim among all users.

Drug (route)	N	%
Alendronate / alendronic acid (oral)	340,810	75.4%
Etidronate (oral)	14	0.0%
Ibandronate / ibandronic acid (oral)	37,988	8.4%
Ibandronic acid (injection/infusion)	1169	0.3%
Pamidronate (injection/infusion)	1121	0.2%
Risedronate (oral)	18,991	4.2%
Zoledronic acid (injection/infusion)	51,958	11.5%

Prior to propensity-score matching, there were significant differences between BP users and non-users across all demographic and clinical characteristics. BP users were older (age >60: 82.7% vs 27.7%; p<0.001), predominantly female (91.0% vs 57.2%; p<0.001), with a higher comorbidity burden (mean CCI 0.95 vs 0.60; p<0.001), with a larger proportion of patients residing in the Western U.S. (21.1% vs 15.4%; p<0.001), covered by Medicare (43.3% vs 13.7%; p<0.001), and having visited a PCP in 2019 (63.8% versus 44.7%; p<0.001). Propensity-score matching yielded 450,366 BP users and 450,366 BP non-users with no significant differences across all characteristics used in matching (Table 2). Differences did exist, however, in the distribution of individual comorbid condition indicators that were used as covariates in the regression analysis, with the BP non-user cohort having a higher proportion of patients with COPD (10.2% vs 8.5%; p<0.001), cardiovascular disease (25.1% vs 18.7%; p<0.001), dyslipidemia (36.9% vs 34.6%; p<0.001), hypertension (46.4% vs 38.8%; p<0.001), obesity (10.3% vs 6.7%; p<0.001), and type 2 diabetes (22.9% vs 18.2%; p<0.001). Over 98% of all BP user/non-user matches for the primary analysis cohort were completed with differences in matched propensity scores <0.000001 (overall mean difference of 0.000004, max difference of 0.0147).

**Table 2.** Primary analysis cohort (all regions), patient characteristics pre/post match.

	All Observations Unmatched							All Observations Matched						
	**All**		**BP Non-users**		**BP Users**		**p-value**	**All**		**BP Non-users**		**BP Users**		**p-value**
	**N**	**%**	**N**	**%**	**N**	**%**		**N**	**%**	**N**	**%**	**N**	**%**	
**All Patients**	**7,906,603**	**100.00%**	**7,454,552**	**94.30%**	**452,051**	**5.70%**		**900,732**	**100.00%**	**450,366**	**50.00%**	**450,366**	**50.00%**	
**Demographics**														
**Age**														
≤20	1,840,050	23.30%	1,838,922	24.70%	1,128	0.20%	<0.001	2,253	0.30%	1,125	0.20%	1,128	0.30%	1
21-40	1,446,999	18.30%	1,443,908	19.40%	3,091	0.70%		6,195	0.70%	3,104	0.70%	3,091	0.70%	
41-50	925,309	11.70%	916,758	12.30%	8,551	1.90%		17,096	1.90%	8,545	1.90%	8,551	1.90%	
51-60	1,250,190	15.80%	1,184,469	15.90%	65,721	14.50%		131,445	14.60%	65,724	14.60%	65,721	14.60%	
61-70	1,181,261	14.90%	1,024,383	13.70%	156,878	34.70%		313,822	34.80%	156,944	34.80%	156,878	34.80%	
71-80	783,775	9.90%	642,050	8.60%	141,725	31.40%		280,803	31.20%	140,366	31.20%	140,437	31.20%	
≥81	479,019	6.10%	404,062	5.40%	74,957	16.60%		149,118	16.60%	74,558	16.60%	74,560	16.60%	
**Gender**														
Female	4,670,960	59.10%	4,263,524	57.20%	407,436	90.10%	<0.001	811,497	90.10%	405,746	90.10%	405,751	90.10%	0.99
Male	3,235,643	40.90%	3,191,028	42.80%	44,615	9.90%		89,235	9.90%	44,620	9.90%	44,615	9.90%	
**Region**														
Midwest	1,467,802	18.60%	1,391,835	18.70%	75,967	16.80%	<0.001	151,802	16.90%	75,901	16.90%	75,901	16.90%	1
Northeast	2,152,560	27.20%	2,032,832	27.30%	119,728	26.50%		238,988	26.50%	119,494	26.50%	119,494	26.50%	
South	3,042,604	38.50%	2,881,718	38.70%	160,886	35.60%		319,408	35.50%	159,704	35.50%	159,704	35.50%	
West	1,243,637	15.70%	1,148,167	15.40%	95,470	21.10%		190,534	21.20%	95,267	21.20%	95,267	21.20%	
**Insurance**														
Commercial	3,938,603	49.80%	3,791,545	50.90%	147,058	32.50%	<0.001	294,070	32.60%	147,012	32.60%	147,058	32.70%	1
Dual	156,497	2.00%	125,090	1.70%	31,407	6.90%		59,936	6.70%	29,980	6.70%	29,956	6.70%	
Medicaid	2,594,500	32.80%	2,517,020	33.80%	77,480	17.10%		154,519	17.20%	77,272	17.20%	77,247	17.20%	
Medicare	1,217,003	15.40%	1,020,897	13.70%	196,106	43.40%		392,207	43.50%	196,102	43.50%	196,105	43.50%	
**PCP Visit 2019**														
No	4,283,697	54.20%	4,119,831	55.30%	163,866	36.20%	<0.001	327,383	36.30%	163,659	36.30%	163,724	36.40%	0.89
Yes	3,622,906	45.80%	3,334,721	44.70%	288,185	63.80%		573,349	63.70%	286,707	63.70%	286,642	63.60%	
**Clinical Characteristics**														
	mean	SD	mean	SD	mean	SD	p-value	mean	SD	mean	SD	mean	SD	p-value
CCI	0.62	1.38	0.6	1.35	0.95	1.76	<0.001	0.95	1.76	0.95	1.76	0.95	1.76	0.7
**Regression Comorbidity Covariates**														
	N	%	N	%	N	%	p-value	N	%	N	%	N	%	p-value
Osteoporosis	267,020	3.40%	135,231	1.80%	131,789	29.20%	<0.001	163,814	18.20%	32,390	7.20%	131,424	29.20%	<0.001
Cancer	419,083	5.30%	366,786	4.90%	52,297	11.60%	<0.001	94,148	10.50%	41,861	9.30%	52,287	11.60%	<0.001
CKD/ESRD	361,451	4.60%	328,633	4.40%	32,818	7.30%	<0.001	68,999	7.70%	36,182	8.00%	32,817	7.30%	<0.001
COPD	466,094	5.90%	427,850	5.70%	38,244	8.50%	<0.001	84,234	9.40%	45,990	10.20%	38,244	8.50%	<0.001
CVD	1,084,031	13.70%	999,526	13.40%	84,505	18.70%	<0.001	197,243	21.90%	112,933	25.10%	84,310	18.70%	<0.001
Dementia	125,811	1.60%	113,778	1.50%	12,033	2.70%	<0.001	24,921	2.80%	12,889	2.90%	12,032	2.70%	<0.001
Depression	571,303	7.20%	531,355	7.10%	39,948	8.80%	<0.001	86,280	9.60%	46,431	10.30%	39,849	8.80%	<0.001
Dyslipidemia	1,532,254	19.40%	1,375,920	18.50%	156,334	34.60%	<0.001	322,125	35.80%	166,360	36.90%	155,765	34.60%	<0.001
HIV/AIDS	33,229	0.40%	31,711	0.40%	1518	0.30%	<0.001	2897	0.30%	1379	0.30%	1,518	0.30%	0.01
Hypertension	1,899,063	24.00%	1,723,519	23.10%	175,544	38.80%	<0.001	384,059	42.60%	209,184	46.40%	174,875	38.80%	<0.001
Liver Disease	251,331	3.20%	231,664	3.10%	19,667	4.40%	<0.001	38,697	4.30%	19,031	4.20%	19,666	4.40%	0.001
Obesity	638,506	8.10%	608,083	8.20%	30,423	6.70%	<0.001	76,844	8.50%	46,498	10.30%	30,346	6.70%	<0.001
Sickle Cell Anemia	10,499	0.10%	10,292	0.10%	207	0.00%	<0.001	422	0.00%	215	0.00%	207	0.00%	0.7
Stroke	104,859	1.30%	97,001	1.30%	7,858	1.70%	<0.001	19,395	2.20%	11,569	2.60%	7,826	1.70%	<0.001
Type 2 Diabetes	978,239	12.40%	895,983	12.00%	82,256	18.20%	<0.001	184,978	20.50%	103,031	22.90%	81,947	18.20%	<0.001

Similar profiles in pre-match *versus* post-match characteristics were seen when patients were stratified by region or restricted to NY-state (Appendix 2—tables 1–3, Appendix 2—table 4, Appendix 2—table 5). Demographic distributions, including differences between BP user *versus* BP non-user characteristics pre-match *versus* post-match characteristics were seens pre- and post-matching for all sensitivity analysis cohorts are detailed in **Appendix 2**.

### BP use and COVID-19-related outcomes

**Figure 2.**
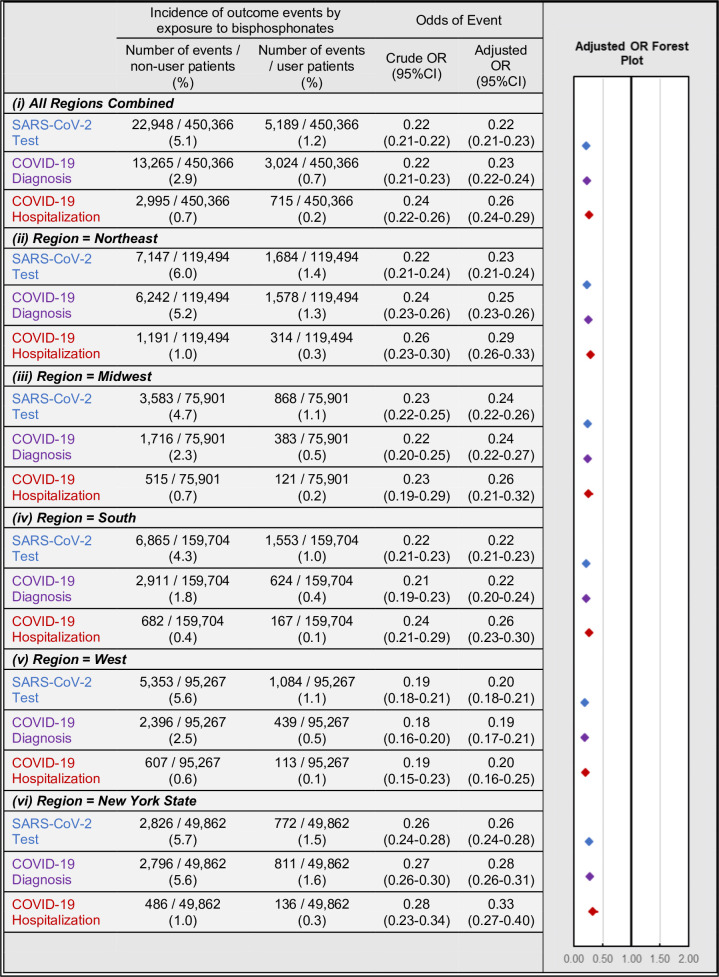
Association of BP use and COVID-19-related outcomes incidence (left) and regression-adjusted results for odds (right) of SARS-CoV-2 testing (blue), COVID-19 diagnosis (purple), and COVID-19-related hospitalizations (red) of BP users compared with BP non-users in the all-regions combined primary analysis cohort (i) and when stratified by region/state into: Northeast (ii), Midwest (iii), South (iv), West (v), and New York state (vi). For details see Figure 2—source data 1. Figure 2—source data 1.COVID-19-related outcomes in the primary analysis cohort.

Among the full matched cohort, BP users had significantly lower rates and unadjusted (crude) odds of testing (1.2% vs 5.1%; OR = 0.22; 95%CI:0.21–0.22; p<0.001), diagnosis (0.7% vs 2.9%; OR = 0.22; 95%CI:0.21–0.23; p<0.001), and hospitalization (0.2% vs 0.7%; OR = 0.24; 95%CI:0.22–0.26; p<0.001) as compared to BP non-users (Figure 2 and Appendix 3—figure 1). Consistent findings were seen when sub-stratifying the full matched cohort by age, gender, age*gender, within grouped regions, by individual region, and in NY-state alone (Appendix 2—tables 6–11).

Multivariate regression analyses yielded similar results for all outcomes while additionally controlling for patient demographic and comorbidity characteristics. In the full matched cohort, BP users had lower adjusted odds of testing (OR = 0.22; 95%CI:0.21–0.23; p<0.001), diagnosis (OR = 0.23; 95%CI:0.22–0.24; p<0.001), and hospitalizations (OR = 0.26; 95%CI:0.24–0.29; p<0.001). These findings were robust when comparing BP users with BP non-users when stratified by geographic region or NY-state alone.

### Timing of last BP exposure and COVID-19-related outcomes

**Figure 3.**
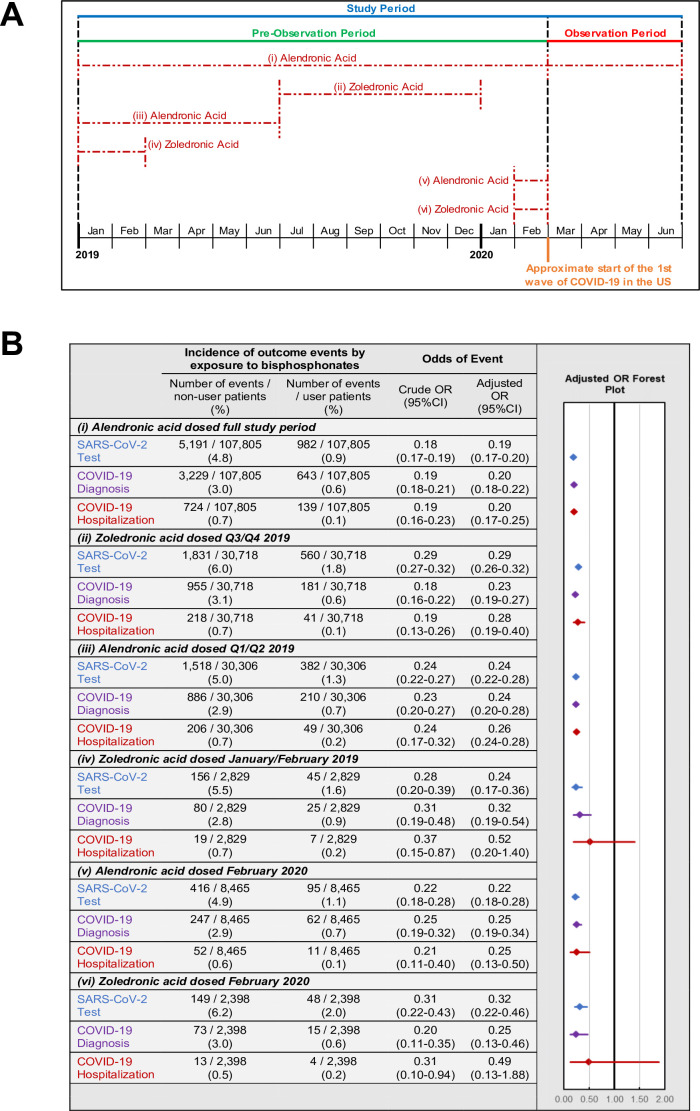
Timing of BP use and COVID-19-related outcomes. (A) Schematic of BP user sub-stratification by timing of exposure to alendronic acid or zoledronic acid prior to outcome assessment. Broken lines represent periods of active BP dosing. For zoledronic acid users, days covered was considered to extend 1 year past the dosing period based on dosing guidelines. (**B**) Incidence (left) and regression-adjusted results (right) for odds of SARS-CoV-2 testing, COVID-19 diagnosis, and COVID-19-related hospitalizations of BP users compared with BP non-users in pre-specified subgroups. For further details see Figure 3—source data 1. *CI: confidence interval; OR: odds ratio*. Figure 3—source data 1.Primary analysis cohort by timing of BP dosing, COVID-19-related outcomes.

The above results demonstrate that any BP exposure during the 14-months pre-observation period is associated with a marked reduction in each of the three COVID-19-related outcomes. To further investigate the relationship between COVID-19-related outcomes and the timing of BP exposure, we focused on the two most commonly prescribed BPs, alendronic acid (oral formulation dosed daily or weekly) and zoledronic acid (infusion dosed annually). For each BP type, COVID-19-related outcomes were assessed among users: (i-ii) with exposure or days covered (based on prescription frequency) during the pre-observation period and throughout the observation period; (iii-iv) with exposure or days covered ending prior to the observation period; and (v-vi) newly initiating therapy prior to the observation period (Figure 3A). Furthermore, all subgroups of BP users had decreased odds of COVID-19-related outcomes (Figure 3B) except for the odds of hospitalization among zoledronic acid users who were last dosed in January/February of 2019 (OR = 0.52; 95%CI:0.20–1.40; p=0.20) or newly initiated in February of 2020 (OR = 0.49; 95%CI:0.13–1.88; p=0.30).

### Sensitivity analysis 1: COVID-19-related outcomes among all users of anti-resorptive medications (‘Bone-Rx’ cohort)

**Figure 4.**
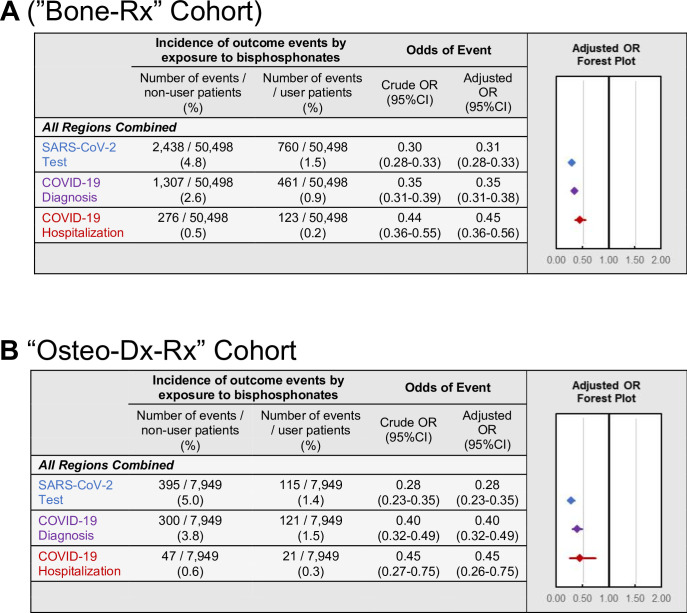
COVID-19-related outcomes among the Bone-RX and Osteo-Dx-Rx restricted cohorts. Incidence and forest plots summarizing regression-adjusted odds ratios of SARS-CoV-2 testing (blue), COVID-19 diagnosis (purple), and COVID-19-related hospitalizations (red) in the (**A**) ‘*Bone-Rx’* (see also Figure 4—source data 1) and (**B**) ‘*Osteo-Dx-Rx’* sensitivity analysis cohorts (see also Figure 4—source data 2). Figure 4—source data 1.Source data for Figure 4A: Bone-Rx cohort COVID-19-related outcomes. Figure 4—source data 2.Source data for Figure 4B: Osteo-Dx-Rx cohort COVID-19-related outcomes.

The first sensitivity analysis was performed to address potential confounding by indication. To validate our primary findings in more comparable cohorts, analysis was restricted to comparing BP users to patients using non-BP anti-resorptive bone medications during the pre-observation period. Compared to non-BP users of anti-resorptive medications, BP users had decreased odds of testing (OR = 0.31; 95%CI:0.28–0.33; p<0.001), diagnosis (OR = 0.35; 95%CI:0.31–0.38; p<0.001), and hospitalization (OR = 0.45; 95%CI:0.36–0.56; p<0.001) (Figure 4A and Appendix 3—figure 2). Furthermore, these findings were robust when assessed separately across every geographic region as well as NY state for all outcomes except hospitalizations when restricted to the Western U.S. (p=0.08; Appendix 2—table 12).

### Sensitivity analysis 2: COVID-19-related outcomes among users of anti-resorptive medications with a diagnosis of osteoporosis (‘Osteo-Dx-Rx’ cohort)

The second sensitivity analysis was performed to address the fact that, even after restricting the comparator cohort to users of anti-resorptive medications, differences may still exist between patient cohorts that could affect COVID-19-related outcomes, including different indications for anti-resorptive medication use and other uncontrolled patient characteristics. To address this, the association between BP use and COVID-19 related outcomes were examined in a cohort restricted to female patients over 50 years old, with a diagnosis of osteoporosis, using either a BP or a non-BP anti-resorptive bone medication, matched within insurance-type as a proxy for socioeconomic status, and selected from four states (NY, IL, FL, CA) with high incidences of COVID-19 cases during the observation period (CDC, 2021a; ‘*Osteo-Dx-Rx*’ cohort). In agreement with the results reported above, the decrease in odds of COVID-19-related outcomes in BP users remained robust for testing (OR = 0.28; 95%CI:0.23–0.35; p<0.001), diagnosis (OR = 0.40; 95%CI:0.32–0.49; p<0.001), and hospitalizations (OR = 0.45; 95%CI:0.26–0.75; p=0.003) (Figure 4B).

### Sensitivity analysis 3: Association of BP-use with exploratory positive control outcomes

**Figure 5.**
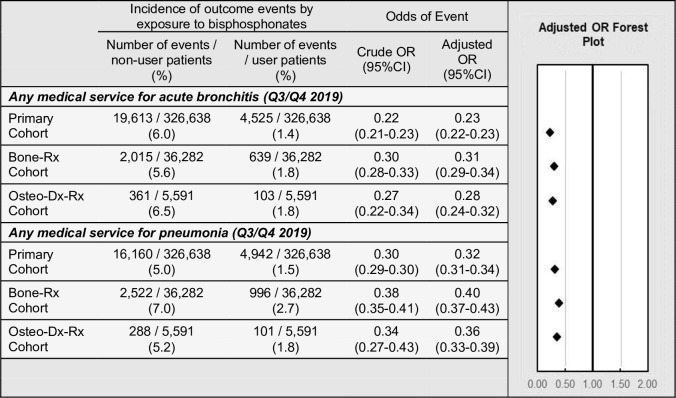
Exploratory outcomes among BP users versus BP non-users. Incidence and adjusted odds ratios of other respiratory infections, in the primary, ‘*Bone-Rx*’, and ‘*Osteo-Dx-Rx*’ cohorts. For details, see Figure 5—source data 1. *CI: confidence interval; OR: odds ratio*. Figure 5—source data 1.Positive control outcomes by primary, bone-Rx, and osteo-Dx-Rx cohorts.

The third sensitivity analysis was performed to assess if there is an association between BP-use and incidence of other respiratory infections, which has been previously reported (Sing et al., 2020). Medical services for acute bronchitis or pneumonia were measured during the second half of 2019, prior to the advent of COVID-19, in the primary, ‘*Bone-Rx’*, and ‘*Osteo-Dx-Rx’* cohorts. Regression modelling found that, among all cohort variations modelled, BP users had a decreased odds of any medical service related to acute bronchitis (point estimates of ORs ranged from 0.23 to 0.28) and pneumonia (point estimates of ORs ranged from 0.32 to 0.36) (Figure 5).

### Sensitivity analysis 4: Association of other preventive drugs with COVID-19-related outcomes

**Figure 6.**
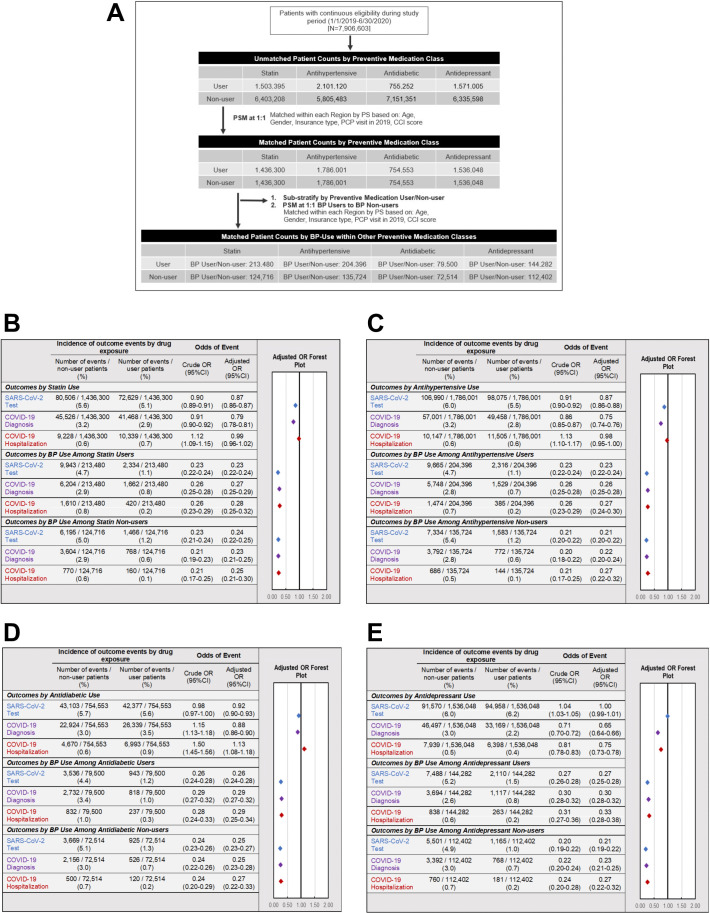
Association of other preventive drugs with COVID-19-related outcomes. (A). Schematic illustrating the identification of study populations and matched controls for each drug class. (**B–E**) Incidence and adjusted odds ratios of SARS-CoV-2 testing (blue), COVID-19 diagnosis (purple), and COVID-19-related hospitalizations (red) in users and non-users of (**B**) statins (see also Figure 6—source data 1), (**C**) antihypertensive medications (see also Figure 6—source data 2), (**D**) non-insulin antidiabetic medications (see also Figure 6—source data 3), and (**E**) antidepressant medications (see also Figure 6—source data 4). For each class of preventive medications, further analysis was performed comparing BP users and BP non-users within matched cohorts of medication users (middle) and medication non-users (bottom). *BP: bisphosphonate; CCI: Charlson comorbidity index; CI: confidence interval; COPD: chronic obstructive pulmonary disease; OR: odds ratio; PCP: primary care physician; PS: propensity score; PSM: propensity score match*. Figure 6—source data 1.Source data for Figure 6B: COVID-19-related outcomes by statin use overall & sub-stratified by BP use. Figure 6—source data 2.Source data for Figure 6C: COVID-19-related outcomes by antihypertensive use overall & sub-stratified by BP use. Figure 6—source data 3.Source data for Figure 6D: COVID-19-related outcomes by antidiabetic use overall & sub-stratified by BP use. Figure 6—source data 4.Source data for Figure 6E: COVID-19-related outcomes by antidepressant use overall & sub-stratified by BP use.

A potential pitfall in the interpretation of apparent effects of preventive medications on health outcomes is the so-called healthy adherer effect, whereby patients may have better outcomes due to their overall healthier behaviours and not due to active drug treatment itself (Ladova et al., 2014). To address this possibility of unmeasured confounding, a final sensitivity analysis was performed to evaluate the association between control exposures (i.e. use of other preventive medications such as statins, antihypertensives, antidiabetics, and antidepressants) and COVID-19-related outcomes (Figure 6A). In comparison to BPs, the impact of other preventive drug classes on COVID-19-related outcomes was much weaker overall (Figure 6B–E) and varied between geographic regions in terms of magnitude or direction (Appendix 2—tables 13–16). Furthermore, when assessing the impact of BP-use within matched user/non-user preventive drug cohorts (e.g. BP users compared to BP non-users among the matched statin user and statin non-user populations), we found BP-use to be consistently associated with lower odds of testing (point estimates of ORs ranged from 0.21 to 0.27), diagnosis (point estimates of ORs ranged from 0.22 to 0.30), and hospitalizations (point estimates of ORs ranged from 0.25 to 0.33) across all stratified preventive user/non-user cohorts (Figure 6B–E).

## Discussion

This study examined the association between recent exposure to BPs and subsequent COVID-19-related outcomes during the initial outbreak of the COVID-19 pandemic in the U.S. Our findings demonstrate that amino-BP users experienced a three- to five-fold reduced incidence of SARS-CoV-2 testing, COVID-19 diagnosis, and COVID-19-related hospitalization during this period. This dramatic difference in outcomes was consistently observed when comparing BP users to BP non-users in a propensity score-matched general population, when comparing to users of other anti-resorptive bone medications, when further restricting the latter cohort to female osteoporosis patients matched by comorbidities within state of residence and by insurance type, and when comparing BP users to BP non-users stratified by use of other preventive medications. Therefore, although there are confounding-related limitations inherent within retrospective studies, the consistency and strength of our observed associations when using various methods to control for unmeasured confounding support the contention that further prospective research should be performed to determine the true magnitude of the potential immunomodulatory effects of BP use.

Our findings are consistent with previous observational studies, prior to the advent of COVID-19, that had reported associations between BP use and reduced incidence of pneumonia and pneumonia-related mortality (Sing et al., 2020; Colón-Emeric et al., 2010; Reid et al., 2021). Accordingly, we observed in our population that BP use was associated with decreased odds of medical services for acute bronchitis and pneumonia during the second half of 2019. Taken together, these findings suggest that BPs may play a protective role in respiratory tract infections from a variety of causes, including SARS-CoV-2.

Other recent retrospective studies have explored, to some extent, associations of anti-resorptive medication use and COVID-19-related outcomes, albeit in much smaller patient populations than were analysed here. One study found no differences in the COVID-19-related risk of hospitalization (70.7% vs 72.7%, p = 0.16) and mortality (11.9% vs 12.8%, p = 0.386) among 1,997 female patients diagnosed with COVID-19 who received anti-osteoporosis medication as compared to propensity score-matched COVID-19 patients who were not receiving such drugs (Atmaca et al., 2022). This study did not examine the incidence of COVID-19 among BP users, but it raises the possibility that the subset of BP users who do develop sufficient pathology to be diagnosed with COVID-19 may have a similar clinical course as BP non-users. Another retrospective cohort study in Italy examining the association of oral amino-BP use and incidence of COVID-19-related hospitalization found no difference between BP users (12.32% (95% CI, 9.61–15.04)) and BP non-users (11.55% (95% CI, 8.91–14.20)) (Degli Esposti et al., 2021). However, the overall incidence of COVID-19 hospitalization in the primary cohort (151/126,370 patients, or 0.12%) of this study was markedly lower than in the present analysis (3,710/900,732 patients, or 0.41%). A third study examined the influence of various anti-osteoporosis drugs, including BPs, on the cumulative incidence of COVID-19 in 2,102 patients with non-inflammatory rheumatic conditions that were compared to population estimates in the same geographic region (Blanch-Rubió et al., 2020). In this analysis, users of non-BP anti-resorptive medications and zoledronate, but not users of oral BPs, had a lower incidence and relative risk of COVID-19 diagnosis and hospitalization. The observations with zoledronate are consistent with the findings reported here. However, we did not detect a significant impact of non-BP anti-resorptive medications in comparison to BPs, and we found a robust association between oral BP use and lower odds of COVID-19 diagnosis and related hospitalization. The reason for these discrepancies is unclear but could potentially reflect the large disparity in sample size between our study, which differed by more than three orders of magnitude. A fourth study, which used Israeli insurance data to perform an analysis involving two separate case-control matched cohorts to assess the risk of COVID-19 hospitalizations when stratified by recent medication use, also found that the odds COVID-19-related hospitalizations were lower among users of BPs, and ranged from an OR of 0.705 (95%CI: 0.522–0.935) to 0.567 (95%CI:0.400–0.789) (Israel et al., 2021).

The large size of our dataset allowed for a range of fully powered, stratified analyses to be performed to explore the robustness of our findings and to address unmeasured confounding factors and other sources of potential bias that can occur in retrospective studies using insurance claims data. Notwithstanding, a retrospective analysis of insurance claims data has inevitable limitations that should be considered. Specifically, there is the potential that key patient characteristics impacting outcomes could not be derived from claims data. For example, the interpretation of our findings depends, in part, on the assumption that BP users and non-users had a similar risk of SARS-CoV-2 infection during the observation period. However, our dataset does not allow us to restrict patient observations to those with known exposure to SARS-CoV-2. Therefore, to minimize potential differences in SARS-CoV-2 exposure between BP users and non-users in our primary study cohort, we implemented additional analytical strategies, including the sensitivity analyses, as well as matching BP users to BP non-users within geographical regions and specific states.

Despite these efforts, it is important to note that we have limited information to assess and match BP users to BP non-users by sociodemographic risk factors, such as socio-economic status and racial/ethnic minority status, that are associated with COVID-19 incidence and mortality (Karmakar et al., 2021; Rogers et al., 2020). Notably, Black/African-American and Hispanic patients have been shown to have significantly higher test positivity rates (Kaufman et al., 2021; Escobar et al., 2021; Jacobson et al., 2021; Rubin-Miller, 2020) and severity of disease at the time of testing (Rubin-Miller, 2020). Furthermore, Black/African American (Azar et al., 2020) and Hispanic patients were found to have a higher incidence of COVID-19 infection (Escobar et al., 2021; CDC, 2021b) and odds of COVID-19 related hospital admission even after adjustment for comorbidities (Nau et al., 2021), residence in a low-income area (Rubin-Miller, 2020), and insurance plan (Azar et al., 2020; Price-Haywood et al., 2020; Muñoz-Price et al., 2020). The greater COVID-19 burden in these groups is likely due to a combination of systemic health inequities as well as a disproportionate representation among essential workers (Selden and Berdahl, 2020; US Bureau of Labor Statistics, 2019), which could potentially increase their exposure risk to SARS-CoV-2. In addition, there are known variations in the prevalence of osteoporosis between different racial groups, which could potentially result in disproportionate frequencies of BP prescriptions (No authors listed, 2021). The potential confounding due to socio-economic status and differential prevalence of osteoporosis among racial/ethnic groups was addressed in our analysis of the ‘*Osteo-Dx-Rx’* cohort where we compared BP users to non-users after restricting to female patients with a diagnosis of osteoporosis, all using anti-resorptive bone medications, and matched by insurance type (proportion of Medicaid and dual Medicare/Medicaid users) as a proxy for social-economic status (Figure 4B). Nevertheless, this strategy cannot rigorously rule out a potential under-representation of groups with higher sociodemographic risk factors among BP users that could have contributed to the observed decreased odds of COVID-19 related outcomes in our primary analyses.

The potential bias introduced by a putative differential racial/ethnic group composition of BP users *versus* BP non-users is at least partially addressed by a recent study of a large Californian cohort of female BP users (Black et al., 2020). Compared to the racial composition of California at-large (a proxy for BP non-users) (United States Census Bureau, 2019), BP users were predominantly Non-Hispanic White (36.5% in California *versus* 53.3% among BP users). The proportions of Black/African-Americans and Asians among BP users in that study were similar to those in California at-large, whereas Hispanic patients represented a smaller percentage (24%) of BP users as compared to Hispanics in the state’s general population (39.4%). Based on these findings and the reported differential case rates of COVID-19 infections among racial groups in California (Reitsma et al., 2021), we can estimate the race-adjusted incidence of COVID-19 in populations reflecting the composition of BP users and non-users (Black et al., 2020) to be 1.7% and 2.1%, respectively. By comparison, in our study the actual rate of COVID-19 diagnosis in the Western US was 2.5% for BP non-users *versus* 0.46% for BP users (Figure 2), indicating that the uneven representation of ethnic/racial groups cannot fully explain the observed differences in COVID-19 related outcomes. Moreover, we note that racial/ethnic minorities are also under-represented among statin users (Salami et al., 2017), but statin-users in our primary cohort had similar odds of COVID-19 hospitalization as statin non-users (Figure 6B). Similarly, Black/African-Americans and Hispanics have lower utilization rates of antidepressants (Chen and Rizzo, 2008) and Hispanics were also reported to be undertreated with antihypertensive medications (Gu et al., 2017). Our analysis of COVID-19-related outcomes among users and non-users of antihypertensives showed a modest decrease in COVID-19 diagnosis and minimal association with COVID-19-related hospitalization (Figure 6C). By contrast, users of antidepressants had uniformly lower odds for both endpoints (Figure 6E), which is consistent with other recent studies (Israel et al., 2021; Hoertel et al., 2021; Zimniak et al., 2021). However, regardless of the class of non-BP preventive drugs analysed, concomitant BP use was consistently associated with dramatically decreased odds of COVID-19 diagnosis and hospitalization as well as testing for SARS-CoV-2 (Figure 6B–E).

Furthermore, specifically looking at the rate of SARS-CoV-2 testing in California (Escobar et al., 2021; Jacobson et al., 2021) or nation-wide (Kaufman et al., 2021), the proportions of different racial and ethnic groups among tested patients were nearly identical to estimates for the state or national population. Thus, the observed association between BP use and reduced testing for SARS-CoV-2 infection in our nation-wide cohorts is unlikely to be explained by potential differences in racial composition between BP users and non-users. It also seems unlikely that exposure to BPs reduces the actual incidence of SARS-CoV-2 infections. More likely, we propose that immune-modulatory effects of BPs may enhance the anti-viral response of BP users to SARS-CoV-2 and mitigate the development of symptoms. Milder or absent symptoms may have caused infected BP users to be less likely to seek testing. Moreover, because there was a nationwide shortage of available tests for SARS-CoV-2 during the observation period, patients needed to present with sufficiently severe disease symptoms to be eligible for testing, so fewer test-seeking BP users may have qualified. Consequently, a larger proportion of uncaptured ’silent' infections among BP users could explain why fewer diagnoses and hospitalizations were observed in this group.

The scarceness of COVID-19 tests combined with the strain on healthcare systems during the observation period could potentially have resulted in a misclassification bias whereby some patients may have been falsely diagnosed and/or hospitalized with COVID-19 without having received a confirmatory test. However, this bias should equally affect BP users and BP non-users and bias our findings towards the null. Relatedly, limited hospital capacity during the observation period could have led to rationing of inpatient hospital beds based on severity of disease and likelihood to survive (Emanuel et al., 2020). However, matching by age and comorbidities should produce patient populations with similar characteristics used for rationing.

A further limitation of our study is the lack of information on the result of COVID-19 tests received by patients. Therefore, as discussed above, the incidence and odds of COVID-19 testing should not be viewed as a proxy for the rate of infection, but rather reflects the incidence of patients with severe enough symptoms or exposure to warrant testing. Another potential source of confounding is the possibility that some patients in our study were classified as BP non-users due to the absence of BP exposure during the pre-observation period but may have received a BP during the observation period. The potential misclassification of BP non-users, however, would bias towards the null hypothesis, and was only seen in 1.92% of the matched BP non-user population.

An additional limitation is potential censoring of patients who died during the observation period, resulting in truncated insurance eligibility and exclusion based on the continuous insurance eligibility requirement. However, modelling the impact of censoring by using death rates observed in BP users and non-users in the first six months of 2020 and attributing all deaths as COVID-19-related did not significantly alter the decreased odds of COVID-19 diagnosis in BP users (see **Appendix 3**).

Another limitation in the current study is related to a potential ‘double correction’ of patient characteristics that were included in both the propensity score matching procedure as well as the outcome regression modelling, which could lead to overfitting of the regression models and an overestimation of the measured treatment effect. Covariates were included in the regression models since these characteristics could have differential impacts on the outcomes themselves, and our results show that the adjusted ORs were in fact slightly larger (showing a decreased effect size) when compared to unadjusted ORs, which show the difference in effect sizes of the matched populations alone.

Furthermore, another potential limitation in both the primary and ‘Bone-Rx’ cohorts is imbalanced comorbidity burden in BP user and non-user cohorts post-match. Table 1 shows there is differential prevalence of most co-morbid diseases despite matched cumulative CCI score between BP user and BP non-user cohorts. However, this limitation is in part addressed given (1) these covariates were controlled for during our regression analyses on study outcomes, and (2) that the key study findings were also observed in the ‘Osteo-Dx-Rx’ cohort, which matched based on individual comorbidities.

Additionally, limitations may be present due to misclassification bias of study outcomes due to the specific procedure/diagnostic codes used as well as the potential for residual confounding occurring for patient characteristics related to study outcomes that are unable to be operationalized in claims data, which would impact all cohort comparisons. For SARS-CoV-2 testing, procedure codes were limited to those testing for active infection, and therefore observations could be missed if they were captured via antibody testing (CPT 86318, 86328). These codes were excluded a priori due to the focus on the symptomatic COVID-19 population. Furthermore, for the COVID-19 diagnosis and hospitalization outcomes, all events were identified using the ICD-10 code for lab-confirmed COVID-19 (U07.1), and therefore events with an associated diagnosis code for suspected COVID-19 (U07.2) were not included. This was done to have a more stringent algorithm when identifying COVID-19-related events, and any impact of events identified using U07.2 is considered minimal, as previous studies of the early COVID-19 outbreak have found that U07.1 alone has a positive predictive value of 94% (Kluberg et al., 2022), and for this study U07.1 captured 99.2%, 99.0%, and 97.5% of all COVID-19 patient-diagnoses for the primary, ‘Bone-Rx’, and ‘Osteo-Dx-Rx’ cohorts, respectively.

Another potential limitation of this study relates to the positivity assumption, which when building comparable treatment cohorts is violated when the comparator population does not have an indication for the exposure being modelled (Petersen et al., 2012). This limitation is present in the primary cohort comparisons between BP users and BP non-users, as well as in the sensitivity analyses involving other preventive medications. This limitation, however, is mitigated by the fact that the outcomes in this study are related to infectious disease and are not direct clinical outcomes of known treatment benefits of BPs. The fact that the clinical benefits being assessed – the impact of BPs on COVID-related outcomes – was essentially unknown clinically at the time of the study data minimizes the impact of violation of the positivity assumption. Furthermore, our sensitivity analyses involving the ‘Bone-Rx’ and ‘Osteo-Dx-Rx’ cohorts did not suffer this potential violation, and the results from those analyses support those from the primary analysis cohort comparisons.

Moreover, we note that the propensity score-matched BP users and BP non-users in the primary analysis cohort mainly consisted of older females. According to the CDC,~75% and 95% of US women between 60–69 and 70–79 suffer from either low bone mass or osteoporosis, respectively (https://www.cdc.gov/nchs/data/databriefs/db93.pdf). Essentially all women (and 70% of men) above age 80 suffer from these conditions, which often go undiagnosed. Women aged 60 and older represent ~75% of our study population (Table 1). Although bone density measurements are not available for non-BP users in the matched primary cohort, there is a high probability that the incidence of osteoporosis and/or low bone mass in these patients was similar to the national average. Thus, BP therapy would have been indicated for most non-BP users in the matched primary cohort, and arguably, for these patients the positivity assumption was not violated.

One large potential bias to consider when comparing BP users to BP non-users is the healthy adherer effect, whereby adherence to drug therapy is associated with overall healthier behavior (Dormuth et al., 2009; Curtis et al., 2011). During the COVID-19 pandemic, this could have potentially resulted in differences between BP users and non-users such as, for example, adherence to mask-wearing, hand washing, or social distancing. However, if this effect accounted for the observed association between BP use and COVID-19-related outcomes, one would expect that users of other preventive medications would show similar associations. However, as discussed above, other preventive drug classes had a variable directional impact on the odds of COVID-19-related events, and sub-analyses within each drug class identified a strong association between concomitant BP use and decreased COVID-19-related events (Figure 6B–E). These analyses were based on the assumption that the association of unmeasured confounders with other drugs is comparable in magnitude and quality as for BPs. Taken together, these results suggest the observed association between BP use and COVID-19-related outcomes cannot solely be attributed to general behaviors associated with the healthy adherer effect.

Notably, several observational studies have reported that the use of one of our comparator preventive drug classes, statins, is associated with a lower risk of mortality in hospitalized COVID-19 patients (Israel et al., 2021; Lohia et al., 2021; Zhang et al., 2020). Indeed, statins are currently being tested as an adjunct therapy for COVID-19 (NCT04380402). In our study population, statin use was associated with moderately decreased odds of SARS-CoV-2 testing and COVID-19 diagnosis, though at a much smaller magnitude than BPs, and was not consistently associated with reduced odds of COVID-19-related hospitalizations. Our analysis did not address the clinical course of hospitalized patients, so these results are not necessarily conflicting. However, we note that in our primary cohort, as many as 15.2% of statin users concomitantly used a BP. Indeed, within statin users, stratification by BP use revealed that the decreased odds of SARS-CoV-2 testing, COVID-19 diagnosis, and COVID-19-related hospitalizations remained regardless of statin use. Future studies on disease outcomes of hospitalized COVID-19 patients with antecedent use of BPs and statins alone or in combination are needed to clarify the effects of each drug class.

The differential association of amino-BPs *versus* statins with COVID-19 related outcomes is somewhat unexpected because both target the same biochemical pathway, albeit at different enzymatic steps (Xia et al., 2018). Statins block HMG-CoA reductase, the first and key rate-limiting enzyme in the mevalonate pathway (Istvan and Deisenhofer, 2001). Amino-BPs, which account for >99% of BPs prescribed in our study, inhibit a downstream enzyme in the same metabolic pathway, farnesyl pyrophosphate synthase (FPPS), which converts geranyl pyrophosphate to farnesyl pyrophosphate (Kavanagh et al., 2006). FPPS blockade disrupts protein prenylation and interferes with cytoskeletal rearrangement, membrane ruffling and vesicular trafficking in osteoclasts, thus preventing bone resorption (Russell, 2007). However, the anti-osteolytic activity of BPs per se is unlikely to account for the observed association between BP use and decreased incidence of COVID-19 and, more broadly, respiratory tract infections, because patients treated with non-BP anti-resorptive bone health medications have higher odds of respiratory infections (Sing et al., 2020 and this study).

Another consequence of mevalonate pathway inhibition by both statins and amino-BPs is arrested endosomal maturation in antigen-presenting cells resulting in enhanced antigen presentation, T cell activation and humoral immunity (Xia et al., 2018). In addition to this adjuvant-like effect, FPPS blockade by amino-BPs causes the intracellular accumulation of the enzyme’s substrate, isopentyl diphosphate (IPP), in myeloid leukocytes, which then stimulate Vγ9Vδ2 T cells (Wang et al., 2011; Nada et al., 2017), a large population of migratory innate lymphocytes in humans that are thought to play an important role in host defense against infectious pathogens (Ribot et al., 2021), including SARS-CoV-1^6^. Experiments in humanized mice that were challenged with influenza viruses have shown that amino-BP-induced expansion of Vγ9Vδ2 T cells markedly improves viral control and mitigates disease severity and mortality (Tu et al., 2011; Zheng et al., 2015). However, since statins act upstream of FPPS, they are expected to inhibit IPP synthesis and, hence, have been shown to counteract the stimulatory effect of amino-BPs on Vγ9Vδ2 T cells (Wang et al., 2011). However, statins and amino-BPs do not always antagonize each other. In vitro, concomitant statin and amino-BP use has been shown to be synergistic in inhibition of cancer cell growth, but mainly through downstream inhibition of geranylgeranyl transferases and subsequent protein prenylation by statins (Abdullah et al., 2017). The fact that the observed reduction in COVID-19-related outcomes in BP users was not altered by concomitant statin use implies that the apparent protective effects of amino-BPs may not rely solely on stimulation of Vγ9Vδ2 T cells. Indeed, in mice (in which BPs are not known to stimulate γδ T cells), BPs potently boost systemic and mucosal antiviral antibody and T cell responses (Tonti et al., 2013). This effect was also seen with non-nitrogenous BPs, which do not antagonize FPPS (Tonti et al., 2013). In the present study, the number of patients who used non-nitrogenous BPs was less than 20, and therefore too small to determine any impact on COVID-19-related outcomes. Nevertheless, in aggregate, these clinical and pre-clinical findings raise the possibility that BPs may exert (at least some) immuno-stimulatory effects by engaging an as yet unidentified additional pathway, regardless of their nitrogen content.

Irrespective of the precise molecular mechanism of action, BPs have been reported to exert a plethora of effects on additional immune cell populations in humans, including NK cells (Sarhan et al., 2017) and regulatory T cells (Liu et al., 2016). Moreover, studies of patients treated with amino-BPs found impaired chemotaxis and generation of reactive oxygen species by neutrophils (Kuiper et al., 2012; Chadwick et al., 2020), a population of inflammatory cells whose dysregulated recruitment and activation are strongly implicated in the pathogenesis of severe COVID-19 (Meizlish et al., 2021; Reusch et al., 2021). Thus, BPs may provide therapeutic benefits during infections with SARS-CoV-2 through modulation of both innate and adaptive immune responses. However, further studies to directly test these pleiotropic immuno-modulatory effects of BPs and to assess their relative contribution to the host response to SARS-CoV-2 infection are needed.

We conclude that, despite several caveats discussed above, the association between BP use and decreased odds of COVID-19-related endpoints was robust in analyses comparing BP users to BP non-users. Large differences were detected regardless of age, sex or geographic location that remained robust when using multiple approaches to address unmeasured confounding and/or potential sources of bias. These retrospective findings strongly suggest that BPs should be considered for prophylactic use in individuals at risk of SARS-CoV-2 infection. However, additional well-controlled prospective clinical studies will be needed to rigorously assess whether the observed reduction in COVID-19-related outcomes is directly caused by BPs and remains true in patient populations not commonly prescribed BPs.

A number of BPs are globally available as relatively affordable generics that are generally well tolerated and could be prescribed for off-label use. Rare, but severe adverse events that have been linked to BP use include osteonecrosis of the jaw (Migliorati et al., 2006) and atypical femur fractures (Saita et al., 2015), which are both associated with long-term BP therapy. In this context, it is important to consider the relationship between the timing of BP exposure and COVID-19-related outcomes. Remarkably, BP users of alendronic acid whose prescription ended more than eight months prior to the observation period, as well as users who initiated alendronic acid therapy immediately preceding the observation period, had similarly decreased odds of COVID-19-related outcomes (Figure 3B). A likely explanation for the observed long-term protection after transient BP use may be the well-documented retention of BPs in bone resulting in half-lives of several years (Cremers et al., 2019). Small amounts of stored BPs are continuously released, especially in regions of high bone turnover, which may result in persistent exposure of immune cells either systemically or preferentially in bone marrow, a site of active immune cell trafficking (Mazo et al., 2005; Zhao et al., 2012) where anti-viral immune responses can be initiated in response to respiratory infection (Hermesh et al., 2010). Thus, BP use at the time of infection may not be necessary for protection against COVID-19. Rather, our results suggest that prophylactic BP therapy may be sufficient to achieve a potentially rapid and sustained immune modulation resulting in profound mitigation of the incidence and/or severity of infections by SARS-CoV-2.

## Figures and Tables

**Appendix 3—figure 1.**
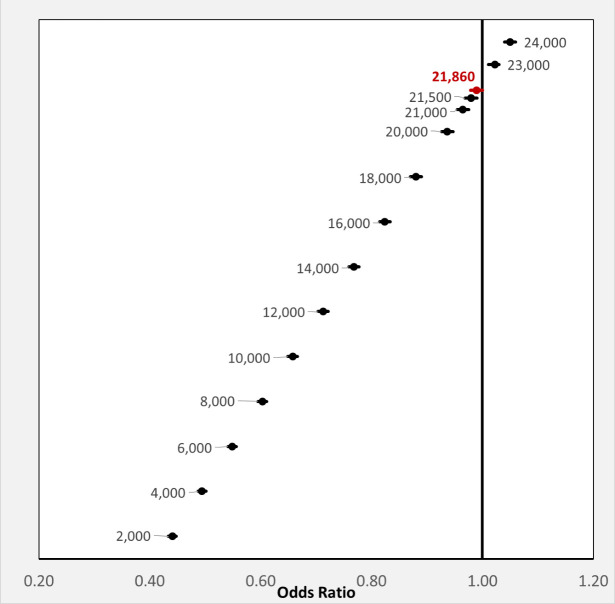
Full cohort: dds ratio by additional number of BP users classified as having COVID-19 diagnosis.Forest plot of the change in the crude odds ratio (OR) of BP users having a COVID-19 diagnosis as a factor of the additional number of BP users needed to be classified as having a COVID-19 diagnosis to reach statistical non-significance for all observations.

**Appendix 3—figure 2.**
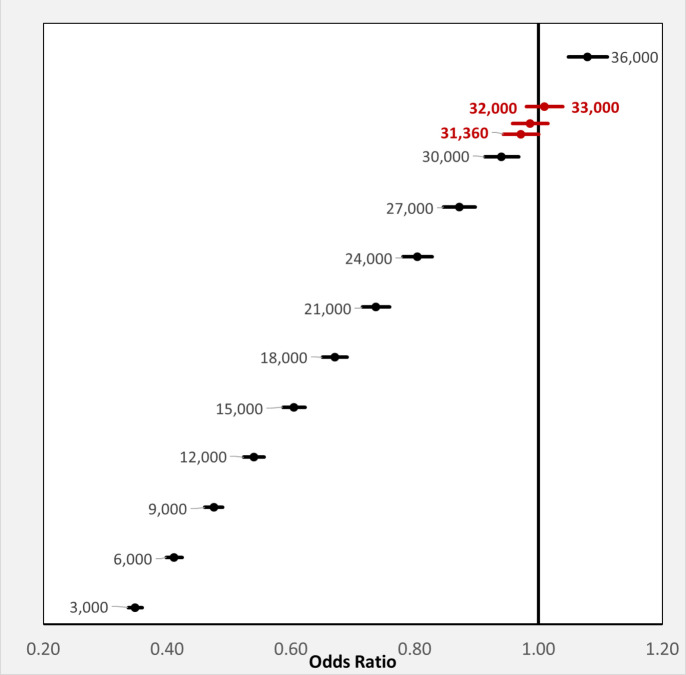
Bone-Rx cohort: odds ratio by additional number of BP users classified as having COVID-19 diagnosis. Forest plot of the change in the crude odds ratio (OR) of BP users having a COVID-19 diagnosis as a factor of the additional number of BP users needed to be classified as having a COVID-19 diagnosis to reach statistical non-significance when comparing BP users to users of non-BP anti-resorptive bone medication

## Data Availability

Excel spreadsheets of source data are provided as supplemental information for figures 1C, 2B, 3A-D, and 4B-E.The administrative claims data used in this study cannot be made publicly available as it as it is a business product of Komodo Health, who contracts with insurers to develop the combined de-identified dataset under agreements that no patient-level data is permitted outside of the Komodo Health analytics environment. All analyses for this current study were performed in the Komodo Health analytics environment.An interested researcher may contact the corresponding author listed in this article by electronic mail at the address listed, who can then further connect them to a researcher at the company who is familiar with the study. The data was analyzed using Microsoft Excel software.

## Appendix 1

### Study Methods

#### Section 1: Variable Assignment

##### Outcomes

The following details the identification algorithms and associated codes that were used to identify outcomes of interest, including COVID-19-related as well as the exploratory outcomes that were assessed during sensitivity analyses.

##### Primary outcomes

SARS-CoV-2 testing

- Any medical services claim with a procedure code indicating polymerase chain reaction (PCR) testing for active SARS-CoV-2 infection 3/1/2020-6/30/2020
- Identified using HCPCS codes: 87635, 87636, 87637

COVID-19 diagnosis

- Any medical services claim with a diagnosis code indicating COVID-19 3/1/2020-6/30/2020
- Identified using ICD-10 code U07.1x

COVID-19-related hospitalization

- Any medical services claim occurring in an inpatient setting with a diagnosis code indicating COVID-19 3/1/2020-6/30/2020
- Identified using ICD-10 code U07.1x

##### Exploratory outcomes (study observation period)

Acute cholecystitis-related service

- Any medical services claim occurring in an emergency room/inpatient setting with a diagnosis indicating acute cholecystitis 3/1/2020-6/30/2020
- Identified using ICD-10 codes K81.0x

Acute pancreatitis-related service

- Any medical services claim occurring in an emergency room/inpatient setting with a diagnosis indicating acute pancreatitis 3/1/2020-6/30/2020
- Identified using ICD-10 codes K85.x

##### Exploratory outcomes (2019)

Acute cholecystitis-related service

- Any medical services claim occurring in an emergency room/inpatient setting with a diagnosis indicating acute cholecystitis 7/1/2019-12/31/2019
- Identified using ICD-10 codes K81.0x

Acute pancreatitis-related service

- Any medical services claim occurring in an emergency room/inpatient setting with a diagnosis indicating acute pancreatitis 7/1/2019-12/31/2019
- Identified using ICD-10 codes K85.x

Acute bronchitis-related service

- Any medical services claim with a diagnosis indicating acute bronchitis 7/1/2019-12/31/2019
- Identified using ICD-10 codes J20.x-J21.x

Acute pneumonia-related service

- Any medical services claim with a diagnosis indicating acute bronchitis 7/1/2019-12/31/2019
- Identified using ICD-10 codes J13.x-J18.x

##### Osteonecrosis

Osteonecrosis

- Any medical services claim with a diagnosis indicating drug-induced osteonecrosis 1/1/2019-6/30/2020
- Identified using ICD-10 codes M87.1x

##### Drug-exposure assignment

The following details the identification algorithms and associated inputs used for drug-exposure classification of study subjects into users/non-users of bisphosphonates, non-bisphosphonates osteoporosis medications, statins, antihypertensives, non-insulin antidiabetics, and antidepressants.

Bisphosphonates

- Any outpatient prescription or in-office dispensing 1/1/2019-2/29/2020
- Drugs included: alendronate, alendronic acid, etidronate, ibandronate, ibandronic acid, pamidronate, risedronate, and zoledronic acid

Non-BP anti-resorptive bone health medications

- Any outpatient prescription or in-office dispensing 1/1/2019-2/29/2020
- Drugs included: denosumab, calcitonin, raloxifene, romosozumab-aqqg, teriparatide, abaloparatide, or bazedoxifene

Statins

- Any outpatient prescription 1/1/2019-2/29/2020
- Drugs included: pravastatin, rosuvastatin, fluvastatin, atorvastatin, pitavastatin, or simvastatin

Antihypertensives

- Any non-ophthalmic, non-injection, outpatient prescription claim for a beta-blocker, calcium channel blocker, or renin angiotensin system antagonist 1/1/2019-2/29/2020
- Drugs included: acebutolol, atenolol, betaxolol, bisoprolol, carvedilol, labetalol, metoprolol, nadolol, nebivolol, penbutolol, pindolol, propranolol, timolol, amlodipine, diltiazem, felodipine, isradipine, nicardipine, nifedipine, nisoldipine, verapamil, aliskiren, azilsartan, benazepril, candesartan, captopril, enalapril, eprosartan, fosinopril, irbesartan, lisinopril, losartan, moexipril, olmesartan, perindopril, quinapril, ramipril, sacubitril, telmisartan, trandolapril, valsartan

Antidiabetics

- Any outpatient prescription claim for a non-insulin antidiabetic medication 1/1/2019-2/29/2020
- Drugs included: metformin, chlorpropamide, glimepiride, glipizide, glyburide, tolazamide, tolbutamide, pioglitazone, rosiglitazone, alogliptin, linagliptin, saxagliptin, sitagliptin, albiglutide, dulaglutide, exenatide, liraglutide, lixisenatide, semaglutide, nateglinide, repaglinide, canagliflozin, dapagliflozin, empagliflozin, ertugliflozin

Antidepressants

- Any outpatient prescription claim for a selective serotonin reuptake inhibitor, norepinephrine-dopamine reuptake inhibitor, serotonin-norepinephrine reuptake inhibitor, tricyclic, tetracyclic, modified cyclic, or MAO inhibitor medication 1/1/2019-2/29/2020
- Drugs included: amoxapine, bupropion, citalopram, clomipramine, desipramine, desvenlafaxine, doxepin, duloxetine, escitalopram, esketamine, fluoxetine, fluvoxamine, imipramine, isocarboxazid, levomilnacipran, maprotiline, mirtazapine, nefazodone, nortriptyline, paroxetine, phenelzine, protriptyline, selegiline, sertraline, tranylcypromine, trazodone, trimipramine, venlafaxine, vilazodone, vortioxetine

##### Charlson comorbidity condition assignment

The following ICD-10 codes were used to assign the CCI condition-specific indicators that are used to calculate the overall CCI score. The time period used for identification of condition-specific indicators was the entire pre-observation period (1/1/2019-2/29/2020).

Myocardial infarction

- ICD-10 codes: I21.x, I22.x, I25.2

Congestive heart failure

- ICD-10 codes: I09.9, I11.0, I13.0, I13.2, I25.5, I42.0, I42.5 - I42.9, I43.x, I50.x, P29.0

Peripheral vascular disease

- ICD-10 codes: I70.x, I71.x, I73.8, I73.9, I77.1, I79.0, I79.2, K55.1, K55.8, K55.9, Z95.8, Z95.9

Cerebrovascular disease

- ICD-10 codes: G45.x, G46.x, H34.0, I60.x-I69.x

Dementia

- ICD-10 codes: F00.x - F03.x, F05.1, G30.x, G31.1

Chronic pulmonary disease

- ICD-10 codes: I27.8, I27.9, J40.x - J47.x, J60.x - J67.x, J68.4, J70.1, J70.3

Rheumatologic disease

- ICD-10 codes: M05.x, M06.x, M31.5, M32.x - M34.x, M35.1, M35.3, M36.0

Peptic ulcer disease

- ICD-10 codes: K25.x-K28.x

Mild liver disease

- ICD-10 codes: B18.x, K70.0 - K70.3, K70.9, K71.3 - K71.5, K71.7, K73.x, K74.x, K76.0, K76.2 - K76.4, K76.8, K76.9, Z94.4

Diabetes without chronic complications

- ICD-10 codes: E10.0, E10.1, E10.6, E10.8, E10.9, E11.0, E11.1, E11.6, E11.8, E11.9, E12.0, E12.1, E12.6, E12.8, E12.9, E13.0, E13.1, E13.6, E13.8, E13.9, E14.0, E14.1, E14.6, E14.8, E14.9

Diabetes with chronic complications

- ICD-10 codes: E10.2 - E10.5, E10.7, E11.2 - E11.5, E11.7, E12.2 - E12.5, E12.7, E13.2 - E13.5, E13.7, E14.2 - E14.5, E14.7

Hemiplegia or paraplegia

- ICD-10 codes: G04.1, G11.4, G80.1, G80.2, G81.x, G82.x, G83.0 - G83.4, G83.9

Renal disease

- ICD-10 codes: I12.0, I13.1, N03.2 - N03.7, N05.2 - N05.7, N18.x, N19.x, N25.0, Z49.0 - Z49.2, Z94.0, Z99.2

Any tumor, leukemia, or lymphoma

- ICD-10 codes: C00.x - C26.x, C30.x - C34.x, C37.x - C41.x, C43.x, C45.x - C58.x, C60.x - C76.x, C81.x - C85.x, C88.x, C90.x - C97.x

Moderate or severe liver disease

- ICD-10 codes: I85.0, I85.9, I86.4, I98.2, K70.4, K71.1, K72.1, K72.9, K76.5, K76.6, K76.7

Metastatic solid tumor

- ICD-10 codes: C77.x - C80.x

AIDS/HIV

- ICD-10 codes: B20.x - B22.x, B24.x

##### Additional condition covariate assignment

The following details the ICD-10 diagnosis codes that were used to identify comorbid conditions. For all condition indicators classification was based on all medical claims occurring during the pre-observation period (1/1/2019-2/29/2020).

Osteoporosis: M80.x, M81.x, M82.x

- Cardiovascular disease overall: I3x.x-I4x.x, I20.x-I28.x, I50.x-I52.x
- Cancer: C0x.x - C9x.x
- Chronic kidney disease (CKD)/ end-stage renal disease (ESRD): I12.0, I13.1, N03.2 - N03.7, N05.2 - N05.7, N18.x, N19.x, N25.0, Z49.0 - Z49.2, Z94.0, Z99.2
- Chronic obstructive pulmonary disease (COPD): J43.x, J44.x
- Dementia: F00.x - F03.x, F05.1, G30.x, G31.1
- Depression: F32.x, F33.x
- Dyslipidemia: E78.x
- Heart failure: I50.x, I11.0xx, I13.0xx, I13.2xx
- HIV/AIDS: B20.x - B22.x, B24.x
- Hypertension: I10.x, I12.x, I11.9xx, I13.1xx
- Liver disease: B18.x, K70.0 - K70.3, K70.9, K71.3 - K71.5, K71.7, K73.x, K74.x, K76.0, K76.2 - K76.4, K76.8, K76.9, Z94.4, I85.0, I85.9, I86.4, I98.2, K70.4, K71.1, K72.1, K72.9, K76.5, K76.6, K76.7
- Obesity: E66.x
- Sickle cell disease: D57.x
- Stroke: I63.x
- Type 2 diabetes: E11.x

#### Sensitivity Analysis (1): COVID-19-related outcomes in “*Bone-Rx*” cohort

##### Overview and rationale

- The first sensitivity analysis was performed to validate the robustness of the primary findings by limiting all BP non-users to those who had used non-BP anti-resorptive bone health medications during the pre-observation period, thus yielding a more comparable comparator cohort that was also receiving bone health medication therapy.
- The use of an active-comparator cohort was done to reduce the impact of unmeasured confounding that may have occurred in the primary analysis due to the use of the derived Charlson Comorbidity Index composite score as the only comorbidity matching covariate. Restriction of the patient population to users of any non-BP anti-resorptive bone health medication prior to propensity-score matching improves the probability of having drug user/non-user matches with more similar clinical characteristics.
- This sensitivity analysis, further, also acted to increase the robustness and reliability of the matched user/non-user outcome comparisons since non-BP anti-resorptive bone health medication users represented the smaller portion of the total bone health medication-user population (“*Bone-Rx*” cohort) and therefore were matched to their best BP-user pair.

##### Analysis cohort definition(s)

- Continuous medical and prescription insurance coverage 1/1/2019-6/30/2020
- Patients with ≥1 claim for any anti-resorptive bone health medication 1/1/2019-2/29/2020

##### Exposures of interest

- Patients were assigned into the BP user cohort if they had any claim 1/1/2019-2/29/2020 for one of the following: alendronate, alendronic acid, etidronate, ibandronate, ibandronic acid, pamidronate, risedronate, and zoledronic acid.
- Patients were assigned into the non-BP any anti-resorptive bone health medication user cohort if: (1) they had any claim 1/1/2019-2/29/2020 for one of the following: denosumab, calcitonin, raloxifene, romosozumab-aqqg, teriparatide, abaloparatide, or bazedoxifene; and (2) they had no BP claims 1/1/2019-2/29/2020.

##### Outcomes

- SARS-CoV-2 testing, COVID-19 diagnosis, and COVID-19-related hospitalizations

##### Cohort matching

- Non-BP anti-resorptive bone health medication users were matched to BP users based on age, gender, insurance type, any PCP visit in 2019, and comorbidity score. Matching was performed within each region separately (northeast, midwest, south, west) and then combined as well as in NY-state alone.

##### Statistical analyses

- Same as was performed for the primary analysis cohort.

#### Sensitivity Analysis (2): COVID-19-related outcomes in “*Osteo-Dx-Rx*” cohort

##### Overview and rationale

- The second sensitivity analysis was performed to further assess the robustness of the primary analysis findings by performing a highly restricted comparator cohort matching that included patients diagnosed and treated for osteoporosis (“*Osteo-Dx-Rx*” cohort).
- The relationship between COVID-19-related outcomes and BP-exposure was modelled after restricting anti-resorptive bone health medication users to those most likely to use BPs and matching BP non-users to BP users based on the presence of comorbid diagnoses within insurance type in four states with early COVID-19 spread representing each to further reduce confounding related to differences in demographic/clinical characteristics amongst BP users/non-users, confounding due to socioeconomic status (insurance type as proxy), and confounding due to differences in COVID-19-exposure risk based on geography.

##### Analysis cohort definition(s)

- Continuous medical and prescription insurance coverage 1/1/2019-6/30/2020
- Patients with ≥1 claim for any osteoporosis medication 1/1/2019-2/29/2020 who also met the following criteria: (i) female; (ii) age 51 or older; (iii) identified as residing in New York, Illinois, Florida, or California; and (iv) had ≥1 medical claim indicating a diagnosis of osteoporosis 1/1/2019-2/29/2020

##### Exposures of interest

- Patients were assigned into the BP user cohort if they had any claim 1/1/2019-2/29/2020 for one of the following: alendronate, alendronic acid, etidronate, ibandronate, ibandronic acid, pamidronate, risedronate, and zoledronic acid.
- Patients were assigned into the non-BP anti-resorptive bone health medication user cohort if: (1) they had any claim 1/1/2019-2/29/2020 for one of the following: denosumab, calcitonin, raloxifene, romosozumab-aqqg, teriparatide, abaloparatide, or bazedoxifene; and (2) they had no BP claims 1/1/2019-2/29/2020.

##### Outcomes

- SARS-CoV-2 testing, COVID-19 diagnosis, and COVID-19-related hospitalizations

##### Cohort matching

- Non- anti-resorptive bone health medication users were matched to BP users based on age, PCP visit in 2019, and the presence of the following comorbid conditions (assigned using ICD-10 codes on claims occurring 1/1/2019-2/29/2020): cancer, chronic obstructive pulmonary disease, depression, dyslipidaemia, heart failure, hypertension, obesity, and type 2 diabetes.
- Matching was performed within each state when stratified by insurance type (commercial, dual, Medicaid, Medicare).

##### Statistical analyses

Multivariate logistic regression analyses, modelled separately for each COVID-19-related outcome of interest, were performed on the unmatched and matched samples after combining all patient observations. In addition to the key exposure variable (indicating BP user versus non-BP user), the regression model also included demographic/clinical covariate for age group, region, insurance type, PCP visit in 2019, and the following comorbid conditions: osteoporosis, cancer, chronic obstructive pulmonary disease, depression, dyslipidaemia, hypertension, obesity, type 2 diabetes, cardiovascular disease overall, sickle cell anemia, stroke, dementia, HIV/AIDS, chronic kidney disease/end-stage renal disease, and liver disease.

#### Sensitivity Analysis (3): Association of BP-use with exploratory negative control outcomes

##### Overview and rationale

- The third sensitivity analysis was performed to assess the relationship between BP-use and outcomes not anticipated to be impacted by the pharmacological mechanism of BPs.
- This was performed by modelling the relationship between BP-exposure and other outcomes occurring (1) during the study observation, and (2) during the second half of 2019 among BP users with claims during the first half of 2019 and their previously-assigned BP non-user matched pair, in the primary, “*Bone-Rx*”, and “*Osteo-Dx-Rx*” cohorts.
- Outcomes modelled included any acute cholecystitis-related or acute pancreatitis-related inpatient/emergency-room (ER) service, used as exploratory outcomes not predicted to be modulated by BP exposure to assess the validity of the core COVID-19-related outcomes.

##### Analysis cohort definition(s)

- Patients who were included in the primary analysis cohort for assessment of (1) outcomes occurring during the study observation period; for (2) outcomes assessed during the second half of 2019 the cohort was restricted to among BP users with claims during the first half of 2019 and their previously-assigned BP non-user matched pair.
- Patients who met all eligibility criteria to be included in the ‘*Bone-Rx’* cohort for assessment of (1) outcomes occurring during the study observation period; for (2) outcomes assessed during the second half of 2019 the cohort was restricted to among BP users with claims during the first half of 2019 and their previously-assigned BP non-user matched pair.
- Patients who met all eligibility criteria to be included in the ‘*Osteo-Dx-Rx’* cohort for assessment of (1) outcomes occurring during the study observation period; for (2) outcomes assessed during the second half of 2019 the cohort was restricted to among BP users with claims during the first half of 2019 and their previously-assigned BP non-user matched pair.

##### Exposures of interest

- For the primary analysis cohort, the BP user / BP non-user assignment was the same as used in the core analyses.
- For the “*Bone-Rx*” and “*Osteo-Dx-Rx*” cohorts, assignment was the same as used in those analyses stratifying medication users into BP users and non-BP medication users.

##### Outcomes

- Any medical claim from an ER/inpatient setting with a diagnosis indicating acute cholecystitis (ICD-10 code K81.0x) occurring 3/1/2020-6/30/2020 (observation period)
- Any medical claim from an ER/inpatient setting with a diagnosis indicating acute pancreatitis (ICD-10 code K85.x) occurring 3/1/2020-6/30/2020 (observation period)
- Any medical claim from an ER/inpatient setting with a diagnosis indicating acute cholecystitis (ICD-10 code K81.0x) occurring 7/1/2019-12/31/2019 (2019)
- Any medical claim from an ER/inpatient setting with a diagnosis indicating acute pancreatitis (ICD-10 code K85.x) occurring 7/1/2019-12/31/2019 (2019)

##### Cohort matching

NA; all cohorts previously matched.

##### Statistical analyses

Multivariate logistic regression analyses were performed using the same methodologies employed when assessing COVID-19 outcomes that were cohort-build-specific (i.e. followed previous approach detailed for each respective cohort build) to assess the odds of acute cholecystitis or acute pancreatitis.

#### Sensitivity Analysis (4): Association of BP-use with exploratory positive control outcomes in 2019

##### Overview and rationale

- The fourth sensitivity analysis was performed to assess the relationship between BP-use and select outcomes occurring in 2019 to validate the theorized BP mechanism of action.
- This was performed by modelling the relationship between BP-exposure in the first half of 2019 and other outcomes occurring during the second half of 2019 in the primary, “*Bone-Rx*”, and “*Osteo-Dx-Rx*” cohorts, specifically medical services for other infectious respiratory conditions (acute bronchitis, pneumonia), used to assess the validity of the relationship between BP-use and decreased respiratory infections.

##### Analysis cohort definition(s)

- The following criteria were applied to all three cohort build variations (primary analysis cohort, “*Bone-Rx*” cohort, “*Osteo-Dx-Rx*” cohort): (i) BP users were restricted to those with any BP claim 1/1/2019-6/30/2019, and the remaining previously-classified BP-user patients with their first BP-claim date occurring on/after 7/1/2019 were excluded; (ii) BP non-users were restricted to their BP-user matched-pair previously assigned.

##### Exposures of interest

- In all cohort build variations, the previously-classified BP user cohorts were restricted to those with any BP-claim 1/1/2019-6/30/2019; all other previously-classified BP users were excluded.

##### Outcomes

- Any medical claim with a diagnosis indicating acute bronchitis (ICD-10 code J20.x-J21.x) occurring 7/1/2019-12/31/2019
- Any medical claim with a diagnosis indicating pneumonia (ICD-10 code J13.x-J18.x) occurring 7/1/2019-12/31/2019

##### Cohort matching

- NA; all cohorts previously matched.

##### Statistical analyses

- Multivariate logistic regression analyses were performed using the same methodologies employed when assessing COVID-19-related outcomes that were cohort-build-specific (i.e. followed previous approach detailed for each respective cohort build) to assess the odds of acute bronchitis, or pneumonia.

#### Sensitivity Analysis (5): Association between use of other drug classes and COVID-19-related outcomes

##### Overview and rationale

- The fifth sensitivity analysis was performed to assess whether the observed protective effect of BPs may be associated with general healthier behaviours in patients using any medication rather than specifically BP use. To assess this unmeasured confounding due to the healthy adherer effect, which is a type of potential bias where patients may have better outcomes due to their heathier behaviours and not better outcomes related to active drug treatment itself, the first sensitivity analysis evaluated the association between use of other preventive medications (statin, antihypertensive, antidiabetic, antidepressant) and COVID-19-related outcomes were evaluated.
- This was performed following the same techniques used in the primary cohort matching and analyses but when assigned drug exposure cohorts based on the use of statin, antihypertensive, antidiabetic, or antidepressant medications. The consistency of methods was done to permit direct comparison on the association between drug-use and COVID-19-related outcomes to assess whether the healthy adherer effect alone accounts for the decrease in the odds of COVID-19 outcomes when comparing BP users to non-users in the primary analysis. Evidence to support the contention that the HAE is a significant source of unmeasured confounding would necessitate that other drug classes display a similar statistically significant trend and/or magnitude when comparing drug users to non-users. Variability in directional impact, magnitude, and/or statistical significance would, conversely, suggest that the healthy adherer effect itself does not account for the differences seen when comparing BP users to BP non-users.
- This sensitivity analysis, additionally, also employed a unique nested-matching technique wherein BP users were matched to BP non-users within the other-medication-class matched populations when stratified into the already matched but mutually exclusive user/non-user cohorts. This was performed to: (1) assess whether the decreased odds of COVID-19-realted outcomes in BP users compared to BP non-users was robust, even amongst cohorts displaying an increase in the odds of COVID-19-related outcomes; and (2) to assess whether the magnitude of decrease in odds of COVID-19-related outcomes amongst BP users compared to BP non-users seen in the primary analysis is impacted by use of other medication classes, including some that have also been identified as being associated with a reduced incidence and/or severity of COVID-19-related outcomes.

##### Analysis cohort definition(s)

- Continuous medical and prescription insurance coverage 1/1/2019-6/30/2020 (*all*)
- Patients with any claim for another drug class of interest (statin, antihypertensive, antidiabetic, antidepressant) medication 1/1/2019-2/29/2020 were classified users
- Among the propensity-score matched drug user/non-user cohorts, a further stratification and propensity-score matching based on BP use 1/1/2019-2/29/2020 to yield the following: (i) drug user/BP user matched to drug user/BP non-user, (ii) drug non-user/BP user matched to drug non-user/BP non-user.

##### Exposures of interest

- Patients were assigned into the statin user cohort if they had any claim 1/1/2019-2/29/2020 for one of the following: pravastatin, rosuvastatin, fluvastatin, atorvastatin, pitavastatin, or simvastatin
- Patients were assigned into the antihypertensive user cohort if they had any non-ophthalmic, non-injection claim 1/1/2019-2/29/2020 for a beta blocker, calcium channel blocker, or renin-angiotensin system antagonist medication.
- Patients were assigned into the antidiabetic user cohort if they had any claim 1/1/2019-2/29/2020 for one of the following non-insulin medications: metformin, chlorpropamide, glimepiride, glipizide, glyburide, tolazamide, tolbutamide, pioglitazone, rosiglitazone, alogliptin, linagliptin, saxagliptin, sitagliptin, albiglutide, dulaglutide, exenatide, liraglutide, lixisenatide, semaglutide, nateglinide, repaglinide, canagliflozin, dapagliflozin, empagliflozin, ertugliflozin
- Patients were assigned into the antidepressant user cohort if they had any claim 1/1/2019-2/29/2020 for one of the following: amoxapine, bupropion, citalopram, clomipramine, desipramine, desvenlafaxine, doxepin, duloxetine, escitalopram, esketamine, fluoxetine, fluvoxamine, imipramine, isocarboxazid, levomilnacipran, maprotiline, mirtazapine, nefazodone, nortriptyline, paroxetine, phenelzine, protriptyline, selegiline, sertraline, tranylcypromine, trazodone, trimipramine, venlafaxine, vilazodone, vortioxetine

##### Outcomes

- SARS-CoV-2 testing, COVID-19 diagnosis, and COVID-19-related hospitalizations

##### Cohort matching

- For the larger drug-class analyses, matching was performed following the same methods used in the primary analysis: users were matched to non-users based on age, gender, insurance type, any PCP visit in 2019, and comorbidity score. Matching was performed within each region separately (northeast, midwest, south, west) and then combined, as well as in NY-state alone.
- Following this matching procedure, a nested BP user to BP non-user propensity score match was then performed on the aforementioned matched populations (i.e. within the separate and already matched statin user and statin non-user populations). Matching was performed using the same list of demographic/clinical characteristics, and was also performed within each region separately (northeast, midwest, south, west) and then combined as well as in NY-state alone.

##### Statistical analyses

- Same as was performed for the primary analysis cohort.

## Appendix 2

### Additional study results; cohort characteristics pre/post match

#### Primary analysis study population

##### Northeast region

A total of 2,152,560 patients identified as residing in the northeast were included in the unmatched primary analysis cohort comparisons, of which 119,728 (5.6%) and 2,032,832 (94.4%) were classified as BP users and BP non-users, respectively (Appendix 2—table 1). Prior to propensity-score matching, there were significant differences across all demographic and clinical characteristics. Compared to BP non-users, BP users were older (97.5% age ≥51 vs 49.8%; *P*<0.001), predominantly female (90.5% vs 57.4%; *P*<0.001), with higher comorbidity burden (mean CCI = 0.93 versus 0.65; *P*<0.001), insured by Medicare (46.5% vs 18.0%; *P*<0.001), and have had a primary-care physician (PCP) visit in 2019 (58.3% vs 42.8%; *P*<0.001). Propensity-score matching yielded 119,494 BP users and 119,494 BP non-users with no significant differences across examined characteristics. A total of 234 BP users from the northeast region in the unmatched primary analysis cohort were not assigned an applicable BP non-user pair during the matching procedure and were excluded from the matched BP user population.

##### Midwest region

A total of 1,467,802 patients identified as residing in the midwest were included in the unmatched primary analysis cohort comparisons, of which 75,967 (5.2%) and 1,391,835 (94.8%) were classified as BP users and BP non-users, respectively (Appendix 2—table 2). Prior to propensity-score matching, there were significant differences across all demographic and clinical characteristics. Compared to BP non-users, BP users were older (96.6% age ≥51 vs 44.0%; *P*<0.001), predominantly female (90.3% vs 57.1%; *P*<0.001), with higher comorbidity burden (mean CCI = 0.99 versus 0.56; *P*<0.001), insured by Medicare (43.6% vs 14.5%; *P*<0.001), and have had a primary-care physician (PCP) visit in 2019 (62.2% vs 51.0%; *P*<0.001). Propensity-score matching yielded 75,901 BP users and 75,901 BP non-users with no significant differences across examined characteristics. A total of 66 BP users from the midwest region in the unmatched primary analysis cohort were not assigned an applicable BP non-user pair during the matching procedure and were excluded from the matched BP user population.

##### South region

A total of 3,042,604 patients identified as residing in the south were included in the unmatched primary analysis cohort comparisons, of which 160,886 (5.3%) and 2,881,718 (94.7%) were classified as BP users and BP non-users, respectively (Appendix 2—table 3). Prior to propensity-score matching, there were significant differences across all demographic and clinical characteristics. Compared to BP non-users, BP users were older (96.8% age ≥51 vs 39.2%; *P*<0.001), predominantly female (90.6% vs 57.4%; *P*<0.001), with higher comorbidity burden (mean CCI = 0.86 versus 0.55; *P*<0.001), insured by Medicare (41.0% vs 11.3%; *P*<0.001), and have had a primary-care physician (PCP) visit in 2019 (66.1% vs 49.2%; *P*<0.001). Propensity-score matching yielded 159,704 BP users and 159,704 BP non-users with no significant differences across examined characteristics. A total of 1,182 BP users from the south region in the unmatched primary analysis cohort were not assigned an applicable BP non-user pair during the matching procedure and were excluded from the matched BP user population.

##### West region

A total of 1,243,637 patients identified as residing in the west were included in the unmatched primary analysis cohort comparisons, of which 95,470 (7.7%) and 1,148,167 (92.3%) were classified as BP users and BP non-users, respectively (Appendix 2—table 4). Prior to propensity-score matching, there were significant differences across all demographic and clinical characteristics. Compared to BP non-users, BP users were older (97.8% age ≥51 vs 43.5%; *P*<0.001), predominantly female (88.7% vs 56.4%; *P*<0.001), with higher comorbidity burden (mean CCI = 1.08 versus 0.66; *P*<0.001), insured by Medicare (43.5% vs 11.0%; *P*<0.001), and have had a primary-care physician (PCP) visit in 2019 (67.7% vs 45.3%; *P*<0.001). Propensity-score matching yielded 95,267 BP users and 95,267 BP non-users with no significant differences across examined characteristics. A total of 203 BP users from the west region in the unmatched primary analysis cohort were not assigned an applicable BP non-user pair during the matching procedure and were excluded from the matched BP user population.

##### New York State

A total of 968,296 patients identified as residing in New York state were included in the unmatched primary analysis NY-state restricted cohort, of which 50,035 (5.2%) and 918,261 (94.8%) were classified as BP users and BP non-users, respectively (Appendix 2—table 5). Prior to propensity-score matching, there were significant differences across all demographic and clinical characteristics. Compared to BP non-users, BP users were older (98.1% age ≥51 vs 50.7%; *P*<0.001), predominantly female (90.9% vs 57.5%; *P*<0.001), with higher comorbidity burden (mean CCI = 0.95 versus 0.63; *P*<0.001), insured by Medicare (57.7% vs 19.5%; *P*<0.001), and have had a primary-care physician (PCP) visit in 2019 (62.7% vs 45.3%; *P*<0. 001). Propensity-score matching yielded 49,862 BP users and 49,862 BP non-users with no significant differences across examined characteristics. A total of 173 BP users from the unmatched New York state primary analysis cohort were not assigned an applicable BP non-user pair during the matching procedure and were excluded from the matched BP user population.

#### Bone-Rx analysis study population

##### All observations (all regions combined)

A total of 502,895 patients were included in the unmatched “*Bone-Rx*” analysis cohort comparisons, of which 452,051 (89.9%) and 50,844 (10.1%) were classified as BP users and BP non-users, respectively (Appendix 2—table 17). Prior to propensity-score matching, there were significant differences across all demographic and clinical characteristics. Compared to BP non-users, BP users were younger (47.9% age ≥71 vs 55.2%; *P*<0.001), predominantly female (90.1% vs 87.2%; *P*<0.001), with a lower comorbidity burden (mean CCI = 0.95 vs 1.99; *P*<0.001), with a larger proportion of patients residing in the west (21.1% versus 15.8%; *P*<0.001), a lower proportion covered by Medicare (43.4% vs 47.5%; *P*<0.001), and a lower proportion have had a primary-care physician (PCP) visit in 2019 (63.8% vs 64.3%; *P*=0.009). Propensity-score matching yielded 50,498 BP users and 50,498 BP non-users with no significant differences across examined characteristics. A total of 346 BP non-users from the unmatched “*Bone-Rx*” analysis cohort were not assigned an applicable BP user pair during the matching procedure and were excluded from the matched BP non-user population.

##### Northeast region

A total of 135,867 patients identified as residing in the northeast were included in the unmatched “*Bone-Rx*” analysis cohort comparisons, of which 119,728 (88.1%) and 16,139 (11.9%) were classified as BP users and BP non-users, respectively (Appendix 2—table 18). Prior to propensity-score matching based on BP-use, there were significant differences across all demographic and clinical characteristics except for any PCP visit in 2019 (*P*=0.95). Compared to BP non-users, BP users were younger (48.1% age ≥71 vs 54.8%; *P*<0.001), predominantly female (90.5% vs 87.5%; *P*<0.001), with a lower comorbidity burden (mean CCI = 0.93 vs 1.97; *P*<0.001), and a lower proportion insured by Medicare (46.5% vs 54.0%; *P*<0.001). Propensity-score matching yielded 15,993 BP users and 15,993 BP non-users with no significant differences across examined characteristics. A total of 146 BP non-users from the northeast region in the unmatched “*Bone-Rx*” analysis cohort were not assigned an applicable BP user pair during the matching procedure and were excluded from the matched BP non-user population.

##### Midwest region

A total of 85,391 patients identified as residing in the midwest were included in the unmatched “*Bone-Rx*” analysis cohort comparisons, of which 75,967 (89.0%) and 9,424 (11.0%) were classified as BP users and BP non-users, respectively (Appendix 2—table 19). Prior to propensity-score matching, there were significant differences across all demographic and clinical characteristics. Compared to BP non-users, BP users were younger (43.0% age ≥71 vs 54.1%; *P*<0.001), predominantly female (90.3% versus 86.1%; *P*<0.001), with a lower comorbidity burden (mean CCI = 0.99 versus 2.12; *P*<0.001), had a lower proportion insured by Medicare (43.6% versus 51.9%; *P*<0.001), with a lower proportion having a primary-care physician (PCP) visit in 2019 (62.2% vs 64.7%; *P*<0.001). Propensity-score matching yielded 9,360 BP users and 9,360 BP non-users with no significant differences across examined characteristics. A total of 64 BP non-users from the midwest region in the unmatched “*Bone-Rx*” analysis cohort were not assigned an applicable BP user pair during the matching procedure and were excluded from the matched BP non-user population.

##### South region

A total of 178,118 patients identified as residing in the south were included in the unmatched “*Bone-Rx*” analysis cohort comparisons, of which 160,886 (90.3%) and 17,232 (9.7%) were classified as BP users and BP non-users, respectively (Appendix 2—table 20). Prior to propensity-score matching, there were significant differences across all demographic and clinical characteristics except for any PCP visit in 2019 (*P*=0.45). Compared to BP non-users, BP users were younger (46.6% age ≥71 vs 53.3%; *P*<0.001), predominantly female (90.6% vs 88.1%; *P*<0.001), with a lower comorbidity burden (mean CCI = 0.86 vs 1.86; *P*<0.001), and a lower proportion insured by Medicare (41.0% vs 44.0%; *P*<0.001). Propensity-score matching yielded 17,140 BP users and 17,140 BP non-users with no significant differences across examined characteristics. A total of 92 BP non-users from the south region in the unmatched “*Bone-Rx*” analysis cohort were not assigned an applicable BP user pair during the matching procedure and were excluded from the matched BP non-user population.

##### West region

A total of 103,519 patients identified as residing in the west were included in the unmatched “*Bone-Rx*” analysis cohort comparisons, of which 95,470 (92.2%) and 8,049 (7.8%) were classified as BP users and BP non-users, respectively (Appendix 2—table 21). Prior to propensity-score matching, there were significant differences across all demographic and clinical characteristics. Compared to BP non-users, BP users were younger (54.1% age ≥71 vs 61.6%; *P*<0.001), predominantly female (88.7% vs 86.2%; *P*<0.001), with a lower comorbidity burden (mean CCI = 1.08 versus 2.17; *P*<0.001), insured by Medicare (43.5% vs 36.9%; *P*<0.001), with a lower proportion having a primary-care physician (PCP) visit in 2019 (67.7% vs 71.6%; *P*<0.001). Propensity-score matching yielded 8,005 BP users and 8,005 BP non-users with no significant differences across examined characteristics. A total of 44 BP non-users from the west region in the unmatched “*Bone-Rx*” analysis cohort were not assigned an applicable BP user pair during the matching procedure and were excluded from the matched BP non-user population.

##### New York State

A total of 57,397 patients identified as residing in New York state were included in the unmatched “*Bone-Rx*” analysis NY-state restricted cohort, of which 50,035 (87.2%) and 7,362 (12.8%) were classified as BP users and BP non-users, respectively (Appendix 2—table 22). Prior to propensity-score matching, there were significant differences across all demographic and clinical characteristics except for any PCP visit in 2019 (*P*=0.35). Compared to BP non-users, BP users were younger (53.2% age ≥11 vs 54.5%; *P*<0.001), predominantly female (90.9% vs 89.5%; *P*<0.001), with a lower comorbidity burden (mean CCI = 0.95 vs 1.81; *P*<0.001), and a higher proportion insured by Medicaid (18.3% vs 13.8%; *P*<0.001). Propensity-score matching yielded 7,254 BP users and 7,254 BP non-users with no significant differences across examined characteristics. A total of 108 BP non-users from the unmatched New York state “*Bone-Rx*” analysis cohort were not assigned an applicable BP user pair during the matching procedure and were excluded from the matched BP non-user population.

#### Osteo-Dx-Rx analysis study population

A total of 60,043 female patients age ≥51 with a diagnosis of osteoporosis who resided in New York (NY), Illinois (IL), Florida (FL), or California (CA) were included in the unmatched “*Osteo-Dx-Rx*” analysis cohort comparison, of which 51,651 (86.0%) and 8,392 (14.0%) were classified as BP users and BP non-users, respectively (Appendix 2—table 23). Prior to propensity-score matching, which was performed within each state by insurance type, there were significant differences across all demographic and clinical characteristics except the proportion of patients with a diagnosis of dyslipidemia (*P*=0.08). Compared to BP non-users, BP users were younger (18.8% age ≥81 vs 26.0%; *P*<0.001), with a larger proportion of patients residing in CA (42.5% vs 30.5%; *P*<0.001), insured by Medicaid (23.1% versus 21.3%; *P*<0.001), have had a primary-care physician (PCP) visit in 2019 (77.4% vs 71.1%; *P*<0.001), had a higher proportion with a diagnosis of obesity (11.2% vs 9.6%; *P*<0.001), and had a lower proportion diagnosed with the following: cancer (11.8% vs 19.4%; *P*<0.001), COPD (10.1% vs 16.2%; *P*<0.001), heart failure (6.1% vs 10.7%; *P*<0.001), hypertension (58.0% vs 60.9%; *P*<0.001), type 2 diabetes (25.6% vs 26.9%; *P*<0.01), and depression (13.9% vs 15.2%; *P*<0.001). Propensity-score matching yielded 7,949 BP users and 7,949 BP non-users with no significant differences across examined characteristics. A total of 443 BP non-users from the unmatched “*Osteo-Dx-Rx*” analysis cohort were not assigned an applicable BP user pair during the matching procedure and were excluded from the matched BP non-user population.

#### Statin user/non-user analysis

##### Statin-use comparison: All observations (all regions combined)

A total of 7,906,603 patients were included in the unmatched analysis cohort comparison of statin-use, of which 1,503,395 (19.0%) and 6,403,208 (81.0%) were classified as statin users and statin non-users, respectively (Appendix 2—table 24). Prior to propensity-score matching, there were significant differences across all demographic and clinical characteristics. Compared to statin non-users, statin users were older (87.9% age ≥51 vs 37.1%; *P*<0.001), with a higher proportion of males (41.1% vs 40.9%; *P*<0.001), from the northeast (29.7% versus 26.6%; *P*<0.001), with higher comorbidity burden (mean CCI = 1.15 vs 0.49; *P*<0.001), insured by Medicare (32.7% vs 11.3%; *P*<0.001), and have had a primary-care physician (PCP) visit in 2019 (66.1% vs 44.1%; *P*<0.001). Propensity-score matching yielded 1,436,300 statin users and 1,436,300 statin non-users with no significant differences across age group, region, insurance type, and having had any PCP visit in 2019. The final matched population did, however, display statistically significant differences between statin users and statin non-users for gender (58.7% vs 58.4% male; *P*<0.001) and mean CCI (1.11 vs 1.12; *P*<0.001). These differences, however, are small in magnitude, and were statistically significant due to the underlying statistical power associated with the large sample size. A total of 67,095 statin users from the unmatched analysis cohort were not assigned an applicable statin non-user pair during the matching procedure and were excluded from the matched statin user population.

##### Statin-use comparison: New York State

A total of 968,296 patients identified as residing in New York state were included in the unmatched analysis cohort comparison of statin-use, of which 206,301 (21.3%) and 761,995 (78.7%) were classified as statin users and statin non-users, respectively (Appendix 2—table 25). Prior to propensity-score matching, there were significant differences across all demographic and clinical characteristics. Compared to statin non-users, statin users were older (90.3% age ≥51 vs 43.1%; *P*<0.001), with a higher proportion of males (42.0% vs 40.4%; *P*<0.001), with higher comorbidity burden (mean CCI = 0.94=0.94=0.94 1.17 vs 0.51; *P*<0.001), insured by Medicare (47.4% versus 14.5%; *P*<0.001), and have had a primary-care physician (PCP) visit in 2019 (64.0% vs 41.3%; *P*<0.001). Propensity-score matching yielded 185,536 statin users and 185,536 statin non-users with no significant differences across age group, gender, insurance type, and having had any PCP visit in 2019. The final matched population did, however, display statistically significant differences between statin users and statin non-users for mean CCI (1.06 vs 1.08; *P*<0.001). This difference, however, is small in magnitude, and was statistically significant due to the underlying statistical power associated with the large sample size. A total of 20,765 statin users from the unmatched analysis cohort were not assigned an applicable statin non-user pair during the matching procedure and were excluded from the matched statin user population.

##### BP-use comparison within statin users: All regions combined

Of the 1,436,300 statin users from the statin user/non-user propensity-score matching analysis, a total of 217,981 (15.2%) and 1,218,319 (84.8%) were classified as BP users and BP non-users, respectively (Appendix 2—table 26). Prior to propensity-score matching based on BP-use, there were significant differences across all demographic and clinical characteristics except for any PCP visit in 2019 (*P*=0.27). Compared to BP non-users, BP users were older (98.9% age ≥51 vs 85.3%; *P*<0.001), with a higher proportion of females (90.1% vs 53.1%; *P*<0.001), from the west (21.7% vs 14.0%; *P*<0.001), with lower comorbidity burden (mean CCI = 0.95 vs 1.13; *P*<0.001), and insured by Medicare (50.8% vs 29.7%; *P*<0.001). Propensity-score matching yielded 213,480 BP users and 213,480 BP non-users with no significant differences across examined characteristics. A total of 4,501 BP users were not assigned an applicable BP non-user pair during the matching procedure and were excluded from the matched BP user population.

##### BP-use comparison within statin users: New York State

Of the 185,536 statin users from the statin user/non-user propensity-score matching analysis on patients residing in New York state, a total of 23,863 (12.9%) and 161,673 (87.1%) were classified as BP users and BP non-users, respectively (Appendix 2—table 27). Prior to propensity-score matching based on BP-use, there were significant differences across all demographic and clinical characteristics except for any PCP visit in 2019 (*P*=0.33). Compared to BP non-users, BP users were older (99.3% age ≥51 vs 87.7%; *P*<0.001), with a higher proportion of females (91.2% vs 53.3%; *P*<0.001), with lower comorbidity burden (mean CCI = 0.92 versus 1.08; *P*<0.001), and insured by Medicare (66.4% vs 41.9%; *P*<0.001). Propensity-score matching yielded 23,736 BP users and 23,736 BP non-users with no significant differences across examined characteristics. A total of 127 BP users were not assigned an applicable BP non-user pair during the matching procedure and were excluded from the matched BP user population.

##### BP-use comparison within statin non-users: All regions combined

Of the 1,436,300 statin non-users from the statin user/non-user propensity-score matching analysis, a total of 124,843 (8.7%) and 1,311,457 (91.3%) were classified as BP users and BP non-users, respectively (Appendix 2—table 28). Prior to propensity-score matching based on BP-use, there were significant differences across all demographic and clinical characteristics. Compared to BP non-users, BP users were older (98.7% age ≥51 vs 86.3%; *P*<0.001), with a higher proportion of females (89.6% vs 55.5%; *P*<0.001), from the west (21.4% vs 14.6%; *P*<0.001), with lower comorbidity burden (mean CCI = 1.02 versus 1.13; *P*<0.001), insured by Medicare (45.8% vs 31.7%; *P*<0.001), and have had a primary-care physician (PCP) visit in 2019 (71.7% vs 63.9%; *P*<0.001). Propensity-score matching yielded 124,716 BP users and 124,716 BP non-users with no significant differences across examined characteristics. A total of 127 BP users were not assigned an applicable BP non-user pair during the matching procedure and were excluded from the matched BP user population.

##### BP-use comparison within statin non-users: New York State

Of the 185,536 statin non-users from the statin user/non-user propensity-score matching analysis on patients residing in New York state, a total of 14,546 (7.8%) and 170,990 (92.2%) were classified as BP users and BP non-users, respectively (Appendix 2—table 29). Prior to propensity-score matching based on BP-use, there were significant differences across all demographic and clinical characteristics. Compared to BP non-users, BP users were older (99.2% age ≥51 vs 88.4%; *P*<0.001), with a higher proportion of females (90.6% vs 55.1%; *P*<0.001), with lower comorbidity burden (mean CCI = 0.95 vs 1.09; *P*<0.001), insured by Medicare (59.7% versus 43.7%; *P*<0.001), and have had a primary-care physician (PCP) visit in 2019 (70.5% vs 59.4%; *P*<0.001). Propensity-score matching yielded 14,521 BP users and 14,521 BP non-users with no significant differences across examined characteristics. A total of 25 BP users were not assigned an applicable BP non-user pair during the matching procedure and were excluded from the matched BP user population.

#### Antihypertensive user/non-user analysis

##### Antihypertensive-use comparison: All observations (all regions combined)

A total of 7,906,603 patients were included in the unmatched analysis cohort comparison of antihypertensive-use, of which 2,101,120 (26.6%) and 5,805,483 (73.4%) were classified as antihypertensive users and antihypertensive non-users, respectively (Appendix 2—table 30). Prior to propensity-score matching, there were significant differences across all demographic and clinical characteristics. Compared to antihypertensive non-users, antihypertensive users were older (80.8% age ≥51 vs 34.4%; *P*<0.001), with a higher proportion of females (60.4% vs 58.6%; *P*<0.001), from the northeast (27.8% vs 27.0%; *P*<0.001), with higher comorbidity burden (mean CCI = 1.13 vs 0.43; *P*<0.001), insured by Medicare (29.5% vs 10.3%; *P*<0.001), and have had a primary-care physician (PCP) visit in 2019 (64.2% vs 39.2%; *P*<0.001). Propensity-score matching yielded 1,786,001 antihypertensive users and 1,786,001 antihypertensive non-users with no significant differences across age group, gender, region, insurance type, and having had any PCP visit in 2019. The final matched population did, however, display statistically significant difference between antihypertensive users and antihypertensive non-users for mean CCI (1.64 vs 1.66; *P*<0.05). This difference, however, is small in magnitude, and was statistically significant due to the underlying statistical power associated with the large sample size. A total of 315,119 antihypertensive users from the unmatched analysis cohort were not assigned an applicable antihypertensive non-user pair during the matching procedure and were excluded from the matched antihypertensive user population.

##### Antihypertensive-use comparison: New York State

A total of 968,296 patients identified as residing in New York state were included in the unmatched analysis cohort comparison of antihypertensive-use, of which 258,652 (26.7%) and 709,644 (73.3%) were classified as antihypertensive users and antihypertensive non-users, respectively (Appendix 2—table 31). Prior to propensity-score matching, there were significant differences across all demographic and clinical characteristics. Compared to antihypertensive non-users, antihypertensive users were older (86.6% age ≥51 vs 40.9%; *P*<0.001), with a higher proportion of females (59.4% vs 59.2%; *P*=0.02), with higher comorbidity burden (mean CCI = 1.17 vs 0.46; *P*<0.001), insured by Medicare (45.9% vs 12.6%; *P*<0.001), and have had a primary-care physician (PCP) visit in 2019 (62.4% vs 40.3%; *P*<0.001). Propensity-score matching yielded 203,624 antihypertensive users and 203,624 antihypertensive non-users with no significant differences across examined characteristics. A total of 55,028 antihypertensive users from the unmatched analysis cohort were not assigned an applicable antihypertensive non-user pair during the matching procedure and were excluded from the matched antihypertensive user population.

##### BP-use comparison within antihypertensive users: All regions combined

Of the 1,786,001 antihypertensive users from the antihypertensive user/non-user propensity-score matching analysis, a total of 206,613 (11.6%) and 1,579,388 (88.4%) were classified as BP users and BP non-users, respectively (Appendix 2—table 32). Prior to propensity-score matching based on BP-use, there were significant differences across all demographic and clinical characteristics. Compared to BP non-users, BP users were older (98.2% age ≥51 vs 75.2%; *P*<0.001), with a higher proportion of females (89.7% vs 56.6%; *P*<0.001), from the west (22.0% vs 14.3%; *P*<0.001), with lower comorbidity burden (mean CCI = 0.94 versus 0.95; *P*=0.02), insured by Medicare (48.6% vs 24.4%; *P*<0.001), and have not had a primary-care physician (PCP) visit in 2019 (41.2% vs 40.1%; *P*<0.001). Propensity-score matching yielded 204,396 BP users and 204,396 BP non-users with no significant differences across examined characteristics. A total of 2,217 BP users were not assigned an applicable BP non-user pair during the matching procedure and were excluded from the matched BP user population.

##### BP-use comparison within antihypertensive users: New York State

Of the 203,624 antihypertensive users from the antihypertensive user/non-user propensity-score matching analysis on patients residing in New York state, a total of 21,213 (10.4%) and 182,411 (89.6%) were classified as BP users and BP non-users, respectively (Appendix 2—table 33). Prior to propensity-score matching based on BP-use, there were significant differences across all demographic and clinical characteristics. Compared to BP non-users, BP users were older (98.8% age ≥51 vs 81.4%; *P*<0.001), with a higher proportion of females (90.9% vs 55.5%; *P*<0.001), with lower comorbidity burden (mean CCI = 0.88 vs 0.95; *P*<0.001), insured by Medicare (64.1% vs 35.9%; *P*<0.001), and have not had a primary-care physician (PCP) visit in 2019 (53.4% vs 55.7%; *P*<0.001). Propensity-score matching yielded 21,126 BP users and 21,126 BP non-users with no significant differences across examined characteristics. A total of 87 BP users were not assigned an applicable BP non-user pair during the matching procedure and were excluded from the matched BP user population.

##### BP-use comparison within antihypertensive non-users: All regions combined

Of the 1,786,001 antihypertensive non-users from the antihypertensive user/non-user propensity-score matching analysis, a total of 136,016 (7.6%) and 1,649,985 (92.4%) were classified as BP users and BP non-users, respectively (Appendix 2—table 34). Prior to propensity-score matching based on BP-use, there were significant differences across all demographic and clinical characteristics. Compared to BP non-users, BP users were older (97.7% age ≥51 vs 76.3%; *P*<0.001), with a higher proportion of females (90.5% vs 58.0%; *P*<0.001), from the west (20.3% vs 14.8%; *P*<0.001), with lower comorbidity burden (mean CCI = 0.88 versus 0.96; *P*<0.001), insured by Medicare (40.7% vs 26.0%; *P*<0.001), and have had a primary-care physician (PCP) visit in 2019 (68.0% vs 59.0%; *P*<0.001). Propensity-score matching yielded 135,724 BP users and 135,724 BP non-users with no significant differences across examined characteristics. A total of 292 BP users were not assigned an applicable BP non-user pair during the matching procedure and were excluded from the matched BP user population.

##### BP-use comparison within antihypertensive non-users: New York State

Of the 203,624 antihypertensive non-users from the antihypertensive user/non-user propensity-score matching analysis on patients residing in New York state, a total of 14,051 (6.9%) and 189,573 (93.1%) were classified as BP users and BP non-users, respectively (Appendix 2—table 35). Prior to propensity-score matching based on BP-use, there were significant differences across all demographic and clinical characteristics. Compared to BP non-users, BP users were older (98.7% age ≥51 vs 82.1%; *P*<0.001), with a higher proportion of females (91.3% vs 56.8%; *P*<0.001), with lower comorbidity burden (mean CCI = 0.81 vs 0.96; *P*<0.001), insured by Medicare (54.9% vs 37.7%; *P*<0.001), and have had a primary-care physician (PCP) visit in 2019 (66.3% vs 54.7%; *P*<0.001). Propensity-score matching yielded 13,983 BP users and 13,983 BP non-users with no significant differences across examined characteristics. A total of 68 BP users were not assigned an applicable BP non-user pair during the matching procedure and were excluded from the matched BP user population.

#### Antidiabetic user/non-user analysis

##### Antidiabetic-use cComparison: All observations (all regions combined)

A total of 7,906,603 patients were included in the unmatched analysis cohort comparison of antidiabetic-use, of which 755,252 (9.6%) and 7,151,351 (90.4%) were classified as antidiabetic users and antidiabetic non-users, respectively (Appendix 2—table 36). Prior to propensity-score matching, there were significant differences across all demographic and clinical characteristics. Compared to antidiabetic non-users, antidiabetic users were older (79.4% age ≥51 vs 43.3%; *P*<0.001), with a higher proportion of females (60.8% vs 58.9%; *P*<0.001), from the northeast (28.8% vs 27.1%; *P*<0.001), with higher comorbidity burden (mean CCI = 1.25 vs 0.55; *P*<0.001), insured by Medicare (26.2% vs 14.2%; *P*<0.001), and have had a primary-care physician (PCP) visit in 2019 (66.5% vs 43.6%; *P*<0.001). Propensity-score matching yielded 754,553 antidiabetic users and 754,553 antidiabetic non-users with no significant differences across examined characteristics. A total of 699 antidiabetic users from the unmatched analysis cohort were not assigned an applicable antidiabetic non-user pair during the matching procedure and were excluded from the matched antidiabetic user population.

##### Antidiabetic-use comparison: New York State

A total of 968,296 patients identified as residing in New York state were included in the unmatched analysis cohort comparison of antidiabetic-use, of which 105,117 (10.9%) and 863,179 (89.1%) were classified as antidiabetic users and antidiabetic non-users, respectively (Appendix 2—table 37). Prior to propensity-score matching, there were significant differences across all demographic and clinical characteristics. Compared to antidiabetic non-users, antidiabetic users were older (83.8% age ≥51 vs 49.4%; *P*<0.001), with a higher proportion of males (42.2% vs 40.6%; *P*<0.001), with higher comorbidity burden (mean CCI = 1.34 vs 0.56; *P*<0.001), insured by Medicare (40.5% vs 19.2%; *P*<0.001), and have had a primary-care physician (PCP) visit in 2019 (64.6% vs 43.9%; *P*<0.001). Propensity-score matching yielded 104,691 antidiabetic users and 104,691 antidiabetic non-users with no significant differences across examined characteristics. A total of 426 antidiabetic users from the unmatched analysis cohort were not assigned an applicable antidiabetic non-user pair during the matching procedure and were excluded from the matched antidiabetic user population.

##### BP-use comparison within antidiabetic users: All regions combined

Of the 754,553 antidiabetic users from the antidiabetic user/non-user propensity-score matching analysis, a total of 80,529 (10.7%) and 674,024 (89.3%) were classified as BP users and BP non-users, respectively (Appendix 2—table 38). Prior to propensity-score matching based on BP-use, there were significant differences across all demographic and clinical characteristics. Compared to BP non-users, BP users were older (98.2% age ≥51 vs 75.2%; *P*<0.001), with a higher proportion of females (98.5% vs 77.1%; *P*<0.001), from the west (22.2% versus 14.2%; *P*<0.001), with a higher comorbidity burden (mean CCI = 1.32 versus 1.23; *P*<0.001), insured by Medicare (45.2% vs 24.0%; *P*<0.001), and have had a primary-care physician (PCP) visit in 2019 (69.5% vs 66.1%; *P*<0.001). Propensity-score matching yielded 79,500 BP users and 79,500 BP non-users with no significant differences across examined characteristics. A total of 1,029 BP users were not assigned an applicable BP non-user pair during the matching procedure and were excluded from the matched BP user population.

##### BP-use comparison within antidiabetic users: New York State

Of the 104,691 antidiabetic users from the antidiabetic user/non-user propensity-score matching analysis on patients residing in New York state, a total of 9,529 (9.1%) and 95,162 (90.9%) were classified as BP users and BP non-users, respectively (Appendix 2—table 39). Prior to propensity-score matching based on BP-use, there were significant differences across all demographic and clinical characteristics. Compared to BP non-users, BP users were older (99.1% age ≥51 vs 82.2%; *P*<0.001), with a higher proportion of females (90.1% vs 54.5%; *P*<0.001), with a higher comorbidity burden (mean CCI = 1.46 vs 1.31; *P*<0.001), insured by Medicare (64.6% vs 38.2%; *P*<0.001), and have had a primary-care physician (PCP) visit in 2019 (66.3% vs 64.4%; *P*<0.001). Propensity-score matching yielded 9,456 BP users and 9,456 BP non-users with no significant differences across examined characteristics. A total of 73 BP users were not assigned an applicable BP non-user pair during the matching procedure and were excluded from the matched BP user population.

##### BP-use comparison within antidiabetic non-users: All regions combined

Of the 754,553 antidiabetic non-users from the antidiabetic user/non-user propensity-score matching analysis, a total of 73,173 (9.7%) and 681,380 (90.3%) were classified as BP users and BP non-users, respectively (Appendix 2—table 40). Prior to propensity-score matching based on BP-use, there were significant differences across all demographic characteristics, but no difference was seen in mean CCI (1.24 vs 1.24; *P*=0.92). Compared to BP non-users, BP users were older (98.0% age ≥51 vs 77.3%; *P*<0.001), with a higher proportion of females (88.9% vs 57.7%; *P*<0.001), from the west (20.1% vs 14.5%; *P*<0.001), insured by Medicare (40.0% vs 24.8%; *P*<0.001), and have had a primary-care physician (PCP) visit in 2019 (74.1% vs 65.7%; *P*<0.001). Propensity-score matching yielded 72,514 BP users and 72,514 BP non-users with no significant differences across examined characteristics. A total of 659 BP users were not assigned an applicable BP non-user pair during the matching procedure and were excluded from the matched BP user population.

##### BP-use comparison within antidiabetic non-users: New York State

Of the 104,691 antidiabetic non-users from the antidiabetic user/non-user propensity-score matching analysis on patients residing in New York state, a total of 9,275 (8.9%) and 95,416 (91.1%) were classified as BP users and BP non-users, respectively (Appendix 2—table 41). Prior to propensity-score matching based on BP-use, there were significant differences across all demographic and clinical characteristics. Compared to BP non-users, BP users were older (99.0% age ≥51 vs 82.2%; *P*<0.001), with a higher proportion of females (89.2% vs 54.7%; *P*<0.001), with a higher comorbidity burden (mean CCI = 1.37 vs 1.32; *P*<0.01), insured by Medicare (57.7% vs 38.9%; *P*<0.001), and have had a primary-care physician (PCP) visit in 2019 (72.5% vs 63.8%; *P*<0.001). Propensity-score matching yielded 13,983 BP users and 13,983 BP non-users with no significant differences across examined characteristics. A total of 131 BP users were not assigned an applicable BP non-user pair during the matching procedure and were excluded from the matched BP user population.

#### Antidepressant user/non-user analysis

##### Antidepressant-use comparison: All observations (all regions combined)

A total of 7,906,603 patients were included in the unmatched analysis cohort comparison of antidepressant-use, of which 1,571,005 (19.9%) and 6,335,598 (80.1%) were classified as antidepressant users and antidepressant non-users, respectively (Appendix 2—table 42). Prior to propensity-score matching, there were significant differences across all demographic and clinical characteristics. Compared to antidepressant non-users, antidepressant users were older (58.6% age ≥51 vs 43.8%; *P*<0.001), with a higher proportion of females (72.8% vs 55.7%; *P*<0.001), from the midwest (22.1% vs 17.7%; *P*<0.001), with higher comorbidity burden (mean CCI = 0.90 vs 0.55; *P*<0.001), insured by Medicare (18.5% vs 14.6%; *P*<0.001), and have had a primary-care physician (PCP) visit in 2019 (61.1% versus 42.0%; *P*<0.001). Propensity-score matching yielded 1,536,048 antidepressant users and 1,536,048 antidepressant non-users with no significant differences across examined characteristics. A total of 34,957 antidepressant users from the unmatched analysis cohort were not assigned an applicable antidepressant non-user pair during the matching procedure and were excluded from the matched antidepressant user population.

##### Antidepressant-use comparison: New York State

A total of 968,296 patients identified as residing in New York state were included in the unmatched analysis cohort comparison of antidepressant-use, of which 136,081 (14.1%) and 832,215 (85.9%) were classified as antidepressant users and antidepressant non-users, respectively (Appendix 2—table 43). Prior to propensity-score matching, there were significant differences across all demographic and clinical characteristics. Compared to antidepressant non-users, antidepressant users were older (66.3% age ≥51 vs 51.0%; *P*<0.001), with a higher proportion of females (71.2% vs 57.3%; *P*<0.001), with higher comorbidity burden (mean CCI = 0.98 vs 0.59; *P*<0.001), insured by Medicare (32.2% vs 19.8%; *P*<0.001), and have had a primary-care physician (PCP) visit in 2019 (60.7% vs 43.8%; *P*<0.001). Propensity-score matching yielded 135,516 antidepressant users and 135,516 antidepressant non-users with no significant differences across examined characteristics. A total of 565 antidepressant users from the unmatched analysis cohort were not assigned an applicable antidepressant non-user pair during the matching procedure and were excluded from the matched antidepressant user population.

##### BP-use comparison within antidepressant users: All regions combined

Of the 1,536,048 antidepressant users from the antidepressant user/non-user propensity-score matching analysis, a total of 145,109 (9.4%) and 1,390,939 (90.6%) were classified as BP users and BP non-users, respectively (Appendix 2—table 44). Prior to propensity-score matching based on BP-use, there were significant differences across all demographic and clinical characteristics. Compared to BP non-users, BP users were older (96.7% age ≥51 vs 54.4%; *P*<0.001), with a higher proportion of females (91.9% vs 70.2%; *P*<0.001), from the west (19.6% versus 13.9%; *P*<0.001), with a higher comorbidity burden (mean CCI = 1.09 versus 0.84; *P*<0.001), insured by Medicare (42.4% vs 16.2%; *P*<0.001), and have had a primary-care physician (PCP) visit in 2019 (64.6% vs 60.2%; *P*<0.001). Propensity-score matching yielded 144,282 BP users and 144,282 BP non-users with no significant differences across examined characteristics. A total of 827 BP users were not assigned an applicable BP non-user pair during the matching procedure and were excluded from the matched BP user population.

##### BP-use comparison within antidepressant users: New York State

Of the 135,516 antidepressant users from the antidepressant user/non-user propensity-score matching analysis on patients residing in New York state, a total of 12,950 (9.6%) and 122,566 (90.4%) were classified as BP users and BP non-users, respectively (Appendix 2—table 45). Prior to propensity-score matching based on BP-use, there were significant differences across all demographic and clinical characteristics. Compared to BP non-users, BP users were older (97.8% age ≥51 vs 63.0%; *P*<0.001), with a higher proportion of females (92.6% vs 68.9%; *P*<0.001), with a higher comorbidity burden (mean CCI = 1.13 vs 0.95; *P*<0.001), insured by Medicare (60.8% vs 29.1%; *P*<0.001), and have had a primary-care physician (PCP) visit in 2019 (65.3% vs 60.1%; *P*<0.001). Propensity-score matching yielded 12,859 BP users and 12,859 BP non-users with no significant differences across examined characteristics. A total of 91 BP users were not assigned an applicable BP non-user pair during the matching procedure and were excluded from the matched BP user population.

##### BP-use comparison within antidepressant non-users: All regions combined

Of the 1,536,048 antidepressant non-users from the antidepressant user/non-user propensity-score matching analysis, a total of 113,110 (7.4%) and 1,422,938 (92.6%) were classified as BP users and BP non-users, respectively (Appendix 2—table 46). Prior to propensity-score matching based on BP-use, there were significant differences across all demographic characteristics. Compared to BP non-users, BP users were older (97.1% age ≥51 vs 55.4%; *P*<0.001), with a higher proportion of females (93.2% vs 70.6%; *P*<0.001), from the west (20.0% versus 14.0%; *P*<0.001), with a higher comorbidity burden (mean CCI = 1.06 versus 0.85; *P*<0.001), insured by Medicare (40.4% vs 17.0%; *P*<0.001), and have had a primary-care physician (PCP) visit in 2019 (71.2% vs 59.8%; *P*<0.001). Propensity-score matching yielded 112,402 BP users and 112,402 BP non-users with no significant differences across examined characteristics. A total of 708 BP users were not assigned an applicable BP non-user pair during the matching procedure and were excluded from the matched BP user population.

##### BP-use comparison within antidepressant non-users: New York State

Of the 135,516 antidepressant non-users from the antidepressant user/non-user propensity-score matching analysis on patients residing in New York state, a total of 10,174 (7.5%) and 125,342 (92.5%) were classified as BP users and BP non-users, respectively (Appendix 2—table 47). Prior to propensity-score matching based on BP-use, there were significant differences across all demographic and clinical characteristics. Compared to BP non-users, BP users were older (98.4% age ≥51 vs 63.7%; *P*<0.001), with a higher proportion of females (93.6% vs 69.4%; *P*<0.001), with a higher comorbidity burden (mean CCI = 1.13 vs 0.95; *P*<0.01), insured by Medicare (60.0% vs 29.9%; *P*<0.001), and have had a primary-care physician (PCP) visit in 2019 (71.7% vs 59.7%; *P*<0.001). Propensity-score matching yielded 10,091 BP users and 10,091 BP non-users with no significant differences across examined characteristics. A total of 83 BP users were not assigned an applicable BP non-user pair during the matching procedure and were excluded from the matched BP user population.

**Appendix 2—table 1.** Primary Analysis Cohort (Region=Northeast), Patient Characteristics Pre/Post Match.

	Region=Northeast Unmatched							Region=Northeast Matched						
	**All**		**BP Non-users**		**BP Users**		**p-value**	**All**		**BP Non-users**		**BP Users**		**p-value**
	**N**	**%**	**N**	**%**	**N**	**%**		**N**	**%**	**N**	**%**	**N**	**%**	
**All Patients**	**2,152,560**	**100.00%**	**2,032,832**	**94.40%**	**119,728**	**5.60%**		**238,988**	**100.00%**	**119,494**	**50.00%**	**119,494**	**50.00%**	
**Age**														
≤20	363,637	16.90%	363,401	17.90%	236	0.20%	<0.001	474	0.20%	238	0.20%	236	0.20%	1
21-40	397,377	18.50%	396,613	19.50%	764	0.60%		1,528	0.60%	764	0.60%	764	0.60%	
41-50	261,570	12.20%	259,528	12.80%	2,042	1.70%		4,084	1.70%	2,042	1.70%	2,042	1.70%	
51-60	372,238	17.30%	354,228	17.40%	18,010	15.00%		36,020	15.10%	18,010	15.10%	18,010	15.10%	
61-70	354,331	16.50%	313,237	15.40%	41,094	34.30%		82,233	34.40%	41,139	34.40%	41,094	34.40%	
71-80	252,712	11.70%	215,151	10.60%	37,561	31.40%		74,831	31.30%	37,393	31.30%	37,438	31.30%	
≥81	150,695	7.00%	130,674	6.40%	20,021	16.70%		39,818	16.70%	19,908	16.70%	19,910	16.70%	
**Gender**														
Female	1,275,611	59.30%	1,167,241	57.40%	108,370	90.50%	<0.001	216,273	90.50%	108,137	90.50%	108,136	90.50%	0.99
Male	876,949	40.70%	865,591	42.60%	11,358	9.50%		22,715	9.50%	11,357	9.50%	11,358	9.50%	
**Insurance**														
Commercial	1,050,795	48.80%	1,017,502	50.10%	33,293	27.80%	<0.001	66,552	27.80%	33,259	27.80%	33,293	27.90%	0.99
Dual	47,773	2.20%	40,168	2.00%	7,605	6.40%		15,114	6.30%	7,576	6.30%	7,538	6.30%	
Medicaid	631,863	29.40%	608,649	29.90%	23,214	19.40%		46,094	19.30%	23,047	19.30%	23,047	19.30%	
Medicare	422,129	19.60%	366,513	18.00%	55,616	46.50%		111,228	46.50%	55,612	46.50%	55,616	46.50%	
**PCP Visit 2019**														
No	1,212,394	56.30%	1,162,527	57.20%	49,867	41.70%	<0.001	99,741	41.70%	49,874	41.70%	49,867	41.70%	0.98
Yes	940,166	43.70%	870,305	42.80%	69,861	58.30%		139,247	58.30%	69,620	58.30%	69,627	58.30%	
**Continuous Outcomes**														
	**mean**	**SD**	**mean**	**SD**	**mean**	**SD**	**p-value**	**mean**	**SD**	**mean**	**SD**	**mean**	**SD**	**p-value**
**CCI**	0.67	1.42	0.65	1.4	0.93	1.71	<0.001	0.93	1.71	0.93	1.71	0.93	1.71	0.96

BP: bisphosphonate; CCI: Charlson Comorbidity Index; PCP: primary care physician; SD: standard deviation.

**Appendix 2—table 2.** Primary Analysis Cohort (Region=Midwest), Patient Characteristics Pre/Post Match.

	Region=Midwest Unmatched							Region=Midwest Matched						
	All		BP Non-users		BP Users		p-value	All		BP Non-users		BP Users		p-value
	N	%	N	%	N	%		N	%	N	%	N	%	
All Patients	1,467,802	100.0%	1,391,835	94.8%	75,967	5.2%		151,802	100.0%	75,901	50.0%	75,901	50.0%	
Age														
≤20	310,027	21.1%	309,759	22.3%	268	0.4%	<0.001	537	0.4%	269	0.4%	268	0.4%	1.00
21-40	287,236	19.6%	286,643	20.6%	593	0.8%		1,188	0.8%	595	0.8%	593	0.8%	
41-50	185,240	12.6%	183,556	13.2%	1,684	2.2%		3,367	2.2%	1,683	2.2%	1,684	2.2%	
51-60	246,230	16.8%	233,992	16.8%	12,238	16.1%		24,478	16.1%	12,240	16.1%	12,238	16.1%	
61-70	224,668	15.3%	196,172	14.1%	28,496	37.5%		56,991	37.5%	28,495	37.5%	28,496	37.5%	
71-80	130,563	8.9%	109,442	7.9%	21,121	27.8%		42,153	27.8%	21,075	27.8%	21,078	27.8%	
≥81	83,838	5.7%	72,271	5.2%	11,567	15.2%		23,088	15.2%	11,544	15.2%	11,544	15.2%	
**Gender**														
Female	863,156	58.8%	794,578	57.1%	68,578	90.3%	<0.001	137,028	90.3%	68,516	90.3%	68,512	90.3%	0.97
Male	604,646	41.2%	597,257	42.9%	7,389	9.7%		14,774	9.7%	7,385	9.7%	7,389	9.7%	
**Insurance**														
Commercial	885,651	60.3%	854,518	61.4%	31,133	41.0%	<0.001	62,243	41.0%	31,110	41.0%	31,133	41.0%	1.00
Dual	28,190	1.9%	24,584	1.8%	3,606	4.7%		7,211	4.8%	3,605	4.7%	3,606	4.8%	
Medicaid	318,596	21.7%	310,473	22.3%	8,123	10.7%		16,136	10.6%	8,079	10.6%	8,057	10.6%	
Medicare	235,365	16.0%	202,260	14.5%	33,105	43.6%		66,212	43.6%	33,107	43.6%	33,105	43.6%	
**PCP Visit 2019**														
No	711,308	48.5%	682,601	49.0%	28,707	37.8%	<0.001	57,398	37.8%	28,691	37.8%	28,707	37.8%	0.93
Yes	756,494	51.5%	709,234	51.0%	47,260	62.2%		94,404	62.2%	47,210	62.2%	47,194	62.2%	
**Continuous Outcomes**														
	**mean**	**SD**	**mean**	**SD**	**mean**	**SD**	**p-value**	**mean**	**SD**	**mean**	**SD**	**mean**	**SD**	**p-value**
**CCI**	0.59	1.37	0.56	1.34	0.99	1.86	<0.001	0.99	1.86	0.99	1.85	1.00	1.86	0.77

BP: bisphosphonate; CCI: Charlson Comorbidity Index; PCP: primary care physician; SD: standard deviation.

**Appendix 2—table 3.** Primary Analysis Cohort (Region=South), Patient Characteristics Pre/Post Match.

	Region=South Unmatched							Region=South Matched						
	All		BP Non-users		BP Users		p-value	All		BP Non-users		BP Users		p-value
	N	%	N	%	N	%		N	%	N	%	N	%	
All Patients	3,042,604	100.0%	2,881,718	94.7%	160,886	5.3%		319,408	100.0%	159,704	50.0%	159,704	50.0%	
Age														
≤20	890,677	29.3%	890,203	30.9%	474	0.3%	<0.001	943	0.3%	469	0.3%	474	0.3%	1.00
21-40	527,971	17.4%	526,794	18.3%	1,177	0.7%		2,364	0.7%	1,187	0.7%	1,177	0.7%	
41-50	338,262	11.1%	334,841	11.6%	3,421	2.1%		6,839	2.1%	3,418	2.1%	3,421	2.1%	
51-60	442,757	14.6%	417,664	14.5%	25,093	15.6%		50,186	15.7%	25,093	15.7%	25,093	15.7%	
61-70	409,854	13.5%	353,958	12.3%	55,896	34.7%		111,800	35.0%	55,904	35.0%	55,896	35.0%	
71-80	272,761	9.0%	222,156	7.7%	50,605	31.5%		99,223	31.1%	49,605	31.1%	49,618	31.1%	
≥81	160,322	5.3%	136,102	4.7%	24,220	15.1%		48,053	15.0%	24,028	15.0%	24,025	15.0%	
**Gender**														
Female	1,800,166	59.2%	1,654,351	57.4%	145,815	90.6%	<0.001	289,263	90.6%	144,630	90.6%	144,633	90.6%	0.99
Male	1,242,438	40.8%	1,227,367	42.6%	15,071	9.4%		30,145	9.4%	15,074	9.4%	15,071	9.4%	
**Insurance**														
Commercial	1,475,456	48.5%	1,416,166	49.1%	59,290	36.9%	<0.001	118,587	37.1%	59,297	37.1%	59,290	37.1%	1.00
Dual	53,474	1.8%	39,414	1.4%	14,060	8.7%		25,752	8.1%	12,874	8.1%	12,878	8.1%	
Medicaid	1,121,606	36.9%	1,099,957	38.2%	21,649	13.5%		43,299	13.6%	21,650	13.6%	21,649	13.6%	
Medicare	392,068	12.9%	326,181	11.3%	65,887	41.0%		131,770	41.3%	65,883	41.3%	65,887	41.3%	
**PCP Visit 2019**														
No	1,701,040	55.9%	1,646,572	57.1%	54,468	33.9%	<0.001	108,601	34.0%	54,275	34.0%	54,326	34.0%	0.85
Yes	1,341,564	44.1%	1,235,146	42.9%	106,418	66.1%		210,807	66.0%	105,429	66.0%	105,378	66.0%	
**Continuous Outcomes**														
	**mean**	**SD**	**mean**	**SD**	**mean**	**SD**	**p-value**	**mean**	**SD**	**mean**	**SD**	**mean**	**SD**	**p-value**
**CCI**	0.57	1.31	0.55	1.28	0.86	1.70	<0.001	0.86	1.70	0.86	1.70	0.86	1.71	0.84

BP: bisphosphonate; CCI: Charlson Comorbidity Index; PCP: primary care physician; SD: standard deviation.

**Appendix 2—table 4.** Primary Analysis Cohort (Region=West), Patient Characteristics Pre/Post Match.

	Region=West Unmatched							Region=West Matched						
	All		BP Non-users		BP Users		p-value	All		BP Non-users		BP Users		p-value
	N	%	N	%	N	%		N	%	N	%	N	%	
All Patients	1,243,637	100.0%	1,148,167	92.3%	95,470	7.7%		190,534	100.0%	95,267	50.0%	95,267	50.0%	
Age														
≤20	275,709	22.2%	275,559	24.0%	150	0.2%	<0.001	299	0.2%	149	0.2%	150	0.2%	1.00
21-40	234,415	18.8%	233,858	20.4%	557	0.6%		1,115	0.6%	558	0.6%	557	0.6%	
41-50	140,237	11.3%	138,833	12.1%	1,404	1.5%		2,806	1.5%	1,402	1.5%	1,404	1.5%	
51-60	188,965	15.2%	178,585	15.6%	10,380	10.9%		20,761	10.9%	10,381	10.9%	10,380	10.9%	
61-70	192,408	15.5%	161,016	14.0%	31,392	32.9%		62,798	33.0%	31,406	33.0%	31,392	33.0%	
71-80	127,739	10.3%	95,301	8.3%	32,438	34.0%		64,596	33.9%	32,293	33.9%	32,303	33.9%	
≥81	84,164	6.8%	65,015	5.7%	19,149	20.1%		38,159	20.0%	19,078	20.0%	19,081	20.0%	
**Gender**														
Female	732,027	58.9%	647,354	56.4%	84,673	88.7%	<0.001	168,933	88.7%	84,463	88.7%	84,470	88.7%	0.96
Male	511,610	41.1%	500,813	43.6%	10,797	11.3%		21,601	11.3%	10,804	11.3%	10,797	11.3%	
**Insurance**														
Commercial	526,701	42.4%	503,359	43.8%	23,342	24.4%	<0.001	46,688	24.5%	23,346	24.5%	23,342	24.5%	1.00
Dual	27,060	2.2%	20,924	1.8%	6,136	6.4%		11,859	6.2%	5,925	6.2%	5,934	6.2%	
Medicaid	522,435	42.0%	497,941	43.4%	24,494	25.7%		48,990	25.7%	24,496	25.7%	24,494	25.7%	
Medicare	167,441	13.5%	125,943	11.0%	41,498	43.5%		82,997	43.6%	41,500	43.6%	41,497	43.6%	
**PCP Visit 2019**														
No	658,955	53.0%	628,131	54.7%	30,824	32.3%	<0.001	61,643	32.4%	30,819	32.4%	30,824	32.4%	0.98
Yes	584,682	47.0%	520,036	45.3%	64,646	67.7%		128,891	67.6%	64,448	67.6%	64,443	67.6%	
**Continuous Outcomes**														
	**mean**	**SD**	**mean**	**SD**	**mean**	**SD**	**p-value**	**mean**	**SD**	**mean**	**SD**	**mean**	**SD**	**p-value**
**CCI**	0.69	1.46	0.66	1.42	1.08	1.84	<0.001	1.09	1.83	1.08	1.83	1.09	1.84	0.73

BP: bisphosphonate; CCI: Charlson Comorbidity Index; PCP: primary care physician; SD: standard deviation.

**Appendix 2—table 5.** Primary Analysis Cohort (Region=New York State), Patient Characteristics Pre/Post Match.

	Region=New York State Unmatched							Region=New York State Matched						
	All		BP Non-users		BP Users		p-value	All		BP Non-users		BP Users		p-value
	N	%	N	%	N	%		N	%	N	%	N	%	
All Patients	968,296	100.0%	918,261	94.8%	50,035	5.2%		99,724	100.0%	49,862	50.0%	49,862	50.0%	
Age														
≤20	133,178	13.8%	133,128	14.5%	50	0.1%	<0.001	102	0.1%	52	0.1%	50	0.1%	1.00
21-40	192,959	19.9%	192,731	21.0%	228	0.5%		453	0.5%	225	0.5%	228	0.5%	
41-50	127,794	13.2%	127,139	13.8%	655	1.3%		1,311	1.3%	656	1.3%	655	1.3%	
51-60	172,444	17.8%	166,080	18.1%	6,364	12.7%		12,732	12.8%	6,368	12.8%	6,364	12.8%	
61-70	159,912	16.5%	143,776	15.7%	16,136	32.2%		32,265	32.4%	16,129	32.3%	16,136	32.4%	
71-80	120,117	12.4%	102,655	11.2%	17,462	34.9%		34,693	34.8%	17,352	34.8%	17,341	34.8%	
≥81	61,892	6.4%	52,752	5.7%	9,140	18.3%		18,168	18.2%	9,080	18.2%	9,088	18.2%	
**Gender**														
Female	573,610	59.2%	528,152	57.5%	45,458	90.9%	<0.001	90,567	90.8%	45,282	90.8%	45,285	90.8%	0.97
Male	394,686	40.8%	390,109	42.5%	4,577	9.1%		9,157	9.2%	4,580	9.2%	4,577	9.2%	
**Insurance**														
Commercial	500,918	51.7%	490,503	53.4%	10,415	20.8%	<0.001	20,830	20.9%	10,415	20.9%	10,415	20.9%	1.00
Dual	6,814	0.7%	5,218	0.6%	1,596	3.2%		3,154	3.2%	1,581	3.2%	1,573	3.2%	
Medicaid	252,366	26.1%	243,191	26.5%	9,175	18.3%		18,044	18.1%	9,019	18.1%	9,025	18.1%	
Medicare	208,198	21.5%	179,349	19.5%	28,849	57.7%		57,696	57.9%	28,847	57.9%	28,849	57.9%	
**PCP Visit 2019**														
No	521,282	53.8%	502,609	54.7%	18,673	37.3%	<0.001	37,253	37.4%	18,616	37.3%	18,637	37.4%	0.89
Yes	447,014	46.2%	415,652	45.3%	31,362	62.7%		62,471	62.6%	31,246	62.7%	31,225	62.6%	
**Continuous Outcomes**														
	**mean**	**SD**	**mean**	**SD**	**mean**	**SD**	**p-value**	**mean**	**SD**	**mean**	**SD**	**mean**	**SD**	**p-value**
**CCI**	0.65	1.39	0.63	1.37	0.95	1.68	<0.001	0.95	1.68	0.95	1.67	0.95	1.68	0.93

BP: bisphosphonate; CCI: Charlson Comorbidity Index; PCP: primary care physician; SD: standard deviation.

**Appendix 2—table 6.** Unadjusted COVID-19-Related Outcomes Stratified by Age, Sex, & Age by Sex; Matched Primary Analysis Cohort, All-Regions Combined.

	Primary Analysis Cohort, All Regions Matched																
	All		SARS-CoV-2 Test					COVID-19 Diagnosis					COVID-19 Hospitalization				
	N	%	N	%	OR		p-value	N	%	OR		p-value	N	%	OR		p-value
					LL	UL				LL	UL				LL	UL	
All Patients	900,732	100.0%	28,137	3.1%				16,289	1.8%				3,710	0.4%			
BP User	450,366	50.0%	5,189	1.2%	0.22		<0.001	3,024	0.7%	0.22		<0.001	715	0.2%	0.24		<0.001
BP Non-user	450,366	50.0%	22,948	5.1%	0.21	0.22		13,265	2.9%	0.21	0.23		2,995	0.7%	0.22	0.26	
**By Age**																	
Age ≤20	2,253	100.0%	67	3.0%				14	0.6%				2	0.1%			
BP User	1,128	50.1%	29	2.6%	0.75		0.26	2	0.2%	0.16		0.007	2	0.2%	NA		NA
BP Non-user	1,125	49.9%	38	3.4%	0.46	1.23		12	1.1%	0.04	0.74		0	0.0%	NA	NA	
Age 21-40	6,195	100.0%	335	5.4%				115	1.9%				13	0.2%			
BP User	3,091	49.9%	58	1.9%	0.20		<0.001	15	0.5%	0.15		<0.001	4	0.1%	0.45		0.27
BP Non-user	3,104	50.1%	277	8.9%	0.15	0.26		100	3.2%	0.08	0.25		9	0.3%	0.14	1.45	
Age 41-50	17,096	100.0%	894	5.2%				270	1.6%				54	0.3%			
BP User	8,551	50.0%	188	2.2%	0.25		<0.001	48	0.6%	0.21		<0.001	14	0.2%	0.35		<0.001
BP Non-user	8,545	50.0%	706	8.3%	0.21	0.29		222	2.6%	0.15	0.29		40	0.5%	0.19	0.64	
Age 51-60	131,445	100.0%	5,765	4.4%				2,371	1.8%				397	0.3%			
BP User	65,721	50.0%	1,104	1.7%	0.22		<0.001	456	0.7%	0.23		<0.001	83	0.1%	0.26		<0.001
BP Non-user	65,724	50.0%	4,661	7.1%	0.21	0.24		1,915	2.9%	0.21	0.26		314	0.5%	0.21	0.34	
Age 61-70	313,822	100.0%	10,438	3.3%				5,029	1.6%				1,035	0.3%			
BP User	156,878	50.0%	1,843	1.2%	0.21		<0.001	939	0.6%	0.23		<0.001	173	0.1%	0.20		<0.001
BP Non-user	156,944	50.0%	8,595	5.5%	0.20	0.22		4,090	2.6%	0.21	0.24		862	0.5%	0.17	0.24	
Age 71-80	280,803	100.0%	7,179	2.6%				4,827	1.7%				1,212	0.4%			
BP User	140,437	50.0%	1,309	0.9%	0.22		<0.001	877	0.6%	0.22		<0.001	234	0.2%	0.24		<0.001
BP Non-user	140,366	50.0%	5,870	4.2%	0.20	0.23		3,950	2.8%	0.20	0.23		978	0.7%	0.21	0.27	
Age ≥81	149,118	100.0%	3,459	2.3%				3,663	2.5%				997	0.7%			
BP User	74,560	50.0%	658	0.9%	0.23		<0.001	687	0.9%	0.22		<0.001	205	0.3%	0.26		<0.001
BP Non-user	74,558	50.0%	2,801	3.8%	0.21	0.25		2,976	4.0%	0.21	0.24		792	1.1%	0.22	0.30	
**Female Patients**	**811,497**	**100.0%**	**24,936**	**3.1%**				**14,367**	**1.8%**				**3,127**	**0.4%**			
BP User	405,751	50.0%	4,519	1.1%	0.21		<0.001	2,667	0.7%	0.22		<0.001	593	0.1%	0.23		<0.001
BP Non-user	405,746	50.0%	20,417	5.0%	0.21	0.22		11,700	2.9%	0.21	0.23		2,534	0.6%	0.21	0.25	
**By Age**																	
Age ≤20	885	100.0%	26	2.9%				7	0.8%				1	0.1%			
BP User	442	49.9%	11	2.5%	0.73		0.43	1	0.2%	0.17		0.12	1	0.2%	NA		NA
BP Non-user	443	50.1%	15	3.4%	0.33	1.60		6	1.4%	0.02	1.38		0	0.0%	NA	NA	
Age 21-40	3,765	100.0%	218	5.8%				64	1.7%				9	0.2%			
BP User	1,879	49.9%	40	2.1%	0.21		<0.001	12	0.6%	0.23		<0.001	3	0.2%	0.50		0.51
BP Non-user	1,886	50.1%	178	9.4%	0.15	0.30		52	2.8%	0.12	0.43		6	0.3%	0.13	2.01	
Age 41-50	13,542	100.0%	730	5.4%				206	1.5%				37	0.3%			
BP User	6,774	50.0%	157	2.3%	0.26		<0.001	43	0.6%	0.26		<0.001	11	0.2%	0.42		0.01
BP Non-user	6,768	50.0%	573	8.5%	0.21	0.31		163	2.4%	0.18	0.36		26	0.4%	0.21	0.85	
Age 51-60	119,205	100.0%	5,200	4.4%				2,093	1.8%				327	0.3%			
BP User	59,602	50.0%	973	1.6%	0.22		<0.001	399	0.7%	0.23		<0.001	64	0.1%	0.24		<0.001
BP Non-user	59,603	50.0%	4,227	7.1%	0.20	0.23		1,694	2.8%	0.21	0.26		263	0.4%	0.18	0.32	
Age 61-70	290,276	100.0%	9,474	3.3%				4,506	1.6%				885	0.3%			
BP User	145,131	50.0%	1,639	1.1%	0.20		<0.001	851	0.6%	0.23		<0.001	144	0.1%	0.19		<0.001
BP Non-user	145,145	50.0%	7,835	5.4%	0.19	0.21		3,655	2.5%	0.21	0.25		741	0.5%	0.16	0.23	
Age 71-80	253,094	100.0%	6,304	2.5%				4,254	1.7%				1,026	0.4%			
BP User	126,559	50.0%	1,140	0.9%	0.21		<0.001	769	0.6%	0.22		<0.001	193	0.2%	0.23		<0.001
BP Non-user	126,535	50.0%	5,164	4.1%	0.20	0.23		3,485	2.8%	0.20	0.23		833	0.7%	0.20	0.27	
Age ≥81	130,730	100.0%	2,984	2.3%				3,237	2.5%				842	0.6%			
BP User	65,364	50.0%	559	0.9%	0.22		<0.001	592	0.9%	0.22		<0.001	177	0.3%	0.26		<0.001
BP Non-user	65,366	50.0%	2,425	3.7%	0.20	0.25		2,645	4.0%	0.20	0.24		665	1.0%	0.22	0.31	
**Male Patients**	**89,235**	**100.0%**	**3,201**	**3.6%**				**1,922**	**2.2%**				**583**	**0.7%**			
BP User	44,615	50.0%	670	1.5%	0.25		<0.001	357	0.8%	0.22		<0.001	122	0.3%	0.26		<0.001
BP Non-user	44,620	50.0%	2,531	5.7%	0.23	0.28		1,565	3.5%	0.20	0.25		461	1.0%	0.22	0.32	
**By Age**																	
Age ≤20	1,368	100.0%	41	3.0%				7	0.5%				1	0.1%			
BP User	686	50.1%	18	2.6%	0.77		0.42	1	0.1%	0.16		0.07	1	0.1%	NA		NA
BP Non-user	682	49.9%	23	3.4%	0.41	1.44		6	0.9%	0.02	1.37		0	0.0%	NA	NA	
Age 21-40	2,430	100.0%	117	4.8%				51	2.1%				4	0.2%			
BP User	1,212	49.9%	18	1.5%	0.17		<0.001	3	0.2%	0.06		<0.001	1	0.1%	0.33		0.63
BP Non-user	1,218	50.1%	99	8.1%	0.10	0.28		48	3.9%	0.02	0.19		3	0.2%	0.03	3.22	
Age 41-50	3,554	100.0%	164	4.6%				64	1.8%				17	0.5%			
BP User	1,777	50.0%	31	1.7%	0.22		<0.001	5	0.3%	0.08		<0.001	3	0.2%	0.21		0.01
BP Non-user	1,777	50.0%	133	7.5%	0.15	0.33		59	3.3%	0.03	0.21		14	0.8%	0.06	0.74	
Age 51-60	12,240	100.0%	565	4.6%				278	2.3%				70	0.6%			
BP User	6,119	50.0%	131	2.1%	0.29		<0.001	57	0.9%	0.25		<0.001	19	0.3%	0.37		<0.001
BP Non-user	6,121	50.0%	434	7.1%	0.24	0.35		221	3.6%	0.19	0.34		51	0.8%	0.22	0.63	
Age 61-70	23,546	100.0%	964	4.1%				523	2.2%				150	0.6%			
BP User	11,747	49.9%	204	1.7%	0.26		<0.001	88	0.7%	0.20		<0.001	29	0.2%	0.24		<0.001
BP Non-user	11,799	50.1%	760	6.4%	0.22	0.30		435	3.7%	0.16	0.25		121	1.0%	0.16	0.36	
Age 71-80	27,709	100.0%	875	3.2%				573	2.1%				186	0.7%			
BP User	13,878	50.1%	169	1.2%	0.23		<0.001	108	0.8%	0.23		<0.001	41	0.3%	0.28		<0.001
BP Non-user	13,831	49.9%	706	5.1%	0.19	0.27		465	3.4%	0.18	0.28		145	1.0%	0.20	0.40	
Age ≥81	18,388	100.0%	475	2.6%				426	2.3%				155	0.8%			
BP User	9,196	50.0%	99	1.1%	0.26		<0.001	95	1.0%	0.28		<0.001	28	0.3%	0.22		<0.001
BP Non-user	9,192	50.0%	376	4.1%	0.20	0.32		331	3.6%	0.22	0.35		127	1.4%	0.14	0.33	

BP: bisphosphonate; LL: lower 95% confidence interval level; NA: not applicable; OR: odds ratio; UL: upper 95% confidence interval level.

**Appendix 2—table 7.** Unadjusted COVID-19-Related Outcomes Stratified by Age, Sex, & Age by Sex; Matched Primary Analysis Cohort, Region=Northeast.

	Region=Northeast Matched																
	All		SARS-CoV-2 Test					COVID-19 Diagnosis					COVID-19 Hospitalization				
	N	%	N	%	OR		p-value	N	%	OR		p-value	N	%	OR		p-value
					LL	UL				LL	UL				LL	UL	
All Patients	238,988	100.0%	8,831	3.7%				7,820	3.3%				1,505	0.6%			
BP User	119,494	50.0%	1,684	1.4%	0.22		<0.001	1,578	1.3%	0.24		<0.001	314	0.3%	0.26		<0.001
BP Non-user	119,494	50.0%	7,147	6.0%	0.21	0.24		6,242	5.2%	0.23	0.26		1,191	1.0%	0.23	0.30	
**By Age**																	
Age ≤20	474	100.0%	14	3.0%				7	1.5%				2	0.4%			
BP User	236	49.8%	7	3.0%	1.01		0.99	2	0.8%	0.40		0.45	2	0.8%	NA		NA
BP Non-user	238	50.2%	7	2.9%	0.35	2.92		5	2.1%	0.08	2.07		0	0.0%	NA	NA	
Age 21-40	1,528	100.0%	93	6.1%				55	3.6%				5	0.3%			
BP User	764	50.0%	14	1.8%	0.16		<0.001	7	0.9%	0.14		<0.001	1	0.1%	0.25		0.37
BP Non-user	764	50.0%	79	10.3%	0.09	0.29		48	6.3%	0.06	0.31		4	0.5%	0.03	2.23	
Age 41-50	4,084	100.0%	234	5.7%				118	2.9%				18	0.4%			
BP User	2,042	50.0%	53	2.6%	0.27		<0.001	17	0.8%	0.16		<0.001	6	0.3%	0.50		0.16
BP Non-user	2,042	50.0%	181	8.9%	0.20	0.37		101	4.9%	0.10	0.27		12	0.6%	0.19	1.33	
Age 51-60	36,020	100.0%	1,863	5.2%				1,190	3.3%				160	0.4%			
BP User	18,010	50.0%	353	2.0%	0.22		<0.001	237	1.3%	0.24		<0.001	38	0.2%	0.31		<0.001
BP Non-user	18,010	50.0%	1,510	8.4%	0.19	0.25		953	5.3%	0.21	0.28		122	0.7%	0.22	0.45	
Age 61-70	82,233	100.0%	3,200	3.9%				2,424	2.9%				403	0.5%			
BP User	41,094	50.0%	597	1.5%	0.22		<0.001	507	1.2%	0.26		<0.001	79	0.2%	0.24		<0.001
BP Non-user	41,139	50.0%	2,603	6.3%	0.20	0.24		1,917	4.7%	0.23	0.28		324	0.8%	0.19	0.31	
Age 71-80	74,831	100.0%	2,266	3.0%				2,306	3.1%				493	0.7%			
BP User	37,438	50.0%	442	1.2%	0.23		<0.001	475	1.3%	0.25		<0.001	99	0.3%	0.25		<0.001
BP Non-user	37,393	50.0%	1,824	4.9%	0.21	0.26		1,831	4.9%	0.23	0.28		394	1.1%	0.20	0.31	
Age ≥81	39,818	100.0%	1,161	2.9%				1,720	4.3%				424	1.1%			
BP User	19,910	50.0%	218	1.1%	0.22		<0.001	333	1.7%	0.23		<0.001	89	0.4%	0.26		<0.001
BP Non-user	19,908	50.0%	943	4.7%	0.19	0.26		1,387	7.0%	0.20	0.26		335	1.7%	0.21	0.33	
**Female Patients**	**216,273**	**100.0%**	**7,897**	**3.7%**				**6,941**	**3.2%**				**1,263**	**0.6%**			
BP User	108,136	50.0%	1,483	1.4%	0.22		<0.001	1,392	1.3%	0.24		<0.001	255	0.2%	0.25		<0.001
BP Non-user	108,137	50.0%	6,414	5.9%	0.21	0.23		5,549	5.1%	0.23	0.26		1,008	0.9%	0.22	0.29	
**By Age**																	
Age ≤20	180	100.0%	4	2.2%				3	1.7%				1	0.6%			
BP User	90	50.0%	2	2.2%	1.00		1.00	1	1.1%	0.49		1.00	1	1.1%	NA		NA
BP Non-user	90	50.0%	2	2.2%	0.14	7.26		2	2.2%	0.04	5.55		0	0.0%	NA	NA	
Age 21-40	864	100.0%	59	6.8%				32	3.7%				4	0.5%			
BP User	431	49.9%	10	2.3%	0.19		<0.001	6	1.4%	0.22		<0.001	1	0.2%	0.33		0.62
BP Non-user	433	50.1%	49	11.3%	0.09	0.37		26	6.0%	0.09	0.54		3	0.7%	0.03	3.22	
Age 41-50	3,176	100.0%	176	5.5%				87	2.7%				13	0.4%			
BP User	1,588	50.0%	40	2.5%	0.28		<0.001	15	0.9%	0.20		<0.001	5	0.3%	0.62		0.40
BP Non-user	1,588	50.0%	136	8.6%	0.19	0.40		72	4.5%	0.11	0.35		8	0.5%	0.20	1.91	
Age 51-60	32,612	100.0%	1,690	5.2%				1,048	3.2%				125	0.4%			
BP User	16,306	50.0%	310	1.9%	0.21		<0.001	206	1.3%	0.24		<0.001	31	0.2%	0.33		<0.001
BP Non-user	16,306	50.0%	1,380	8.5%	0.18	0.24		842	5.2%	0.20	0.27		94	0.6%	0.22	0.49	
Age 61-70	76,403	100.0%	2,933	3.8%				2,181	2.9%				343	0.4%			
BP User	38,200	50.0%	536	1.4%	0.21		<0.001	456	1.2%	0.26		<0.001	63	0.2%	0.22		<0.001
BP Non-user	38,203	50.0%	2,397	6.3%	0.19	0.23		1,725	4.5%	0.23	0.28		280	0.7%	0.17	0.29	
Age 71-80	67,857	100.0%	2,021	3.0%				2,063	3.0%				416	0.6%			
BP User	33,930	50.0%	393	1.2%	0.23		<0.001	413	1.2%	0.24		<0.001	77	0.2%	0.23		<0.001
BP Non-user	33,927	50.0%	1,628	4.8%	0.21	0.26		1,650	4.9%	0.22	0.27		339	1.0%	0.18	0.29	
Age ≥81	35,181	100.0%	1,014	2.9%				1,527	4.3%				361	1.0%			
BP User	17,591	50.0%	192	1.1%	0.23		<0.001	295	1.7%	0.23		<0.001	77	0.4%	0.27		<0.001
BP Non-user	17,590	50.0%	822	4.7%	0.19	0.26		1,232	7.0%	0.20	0.26		284	1.6%	0.21	0.34	
**Male Patients**	**22,715**	**100.0%**	**934**	**4.1%**				**879**	**3.9%**				**242**	**1.1%**			
BP User	11,358	50.0%	201	1.8%	0.26		<0.001	186	1.6%	0.26		<0.001	59	0.5%	0.32		<0.001
BP Non-user	11,357	50.0%	733	6.5%	0.22	0.31		693	6.1%	0.22	0.30		183	1.6%	0.24	0.43	
**By Age**																	
Age ≤20	294	100.0%	10	3.4%				4	1.4%				1	0.3%			
BP User	146	49.7%	5	3.4%	1.01		0.98	1	0.7%	0.33		0.62	1	0.7%	NA		NA
BP Non-user	148	50.3%	5	3.4%	0.29	3.58		3	2.0%	0.03	3.24		0	0.0%	NA	NA	
Age 21-40	664	100.0%	34	5.1%				23	3.5%				1	0.2%			
BP User	333	50.2%	4	1.2%	0.12		<0.001	1	0.3%	0.04		<0.001	0	0.0%	NA		NA
BP Non-user	331	49.8%	30	9.1%	0.04	0.35		22	6.6%	0.01	0.32		1	0.3%	NA	NA	
Age 41-50	908	100.0%	58	6.4%				31	3.4%				5	0.6%			
BP User	454	50.0%	13	2.9%	0.27		<0.001	2	0.4%	0.06		<0.001	1	0.2%	0.25		0.37
BP Non-user	454	50.0%	45	9.9%	0.14	0.50		29	6.4%	0.02	0.27		4	0.9%	0.03	2.23	
Age 51-60	3,408	100.0%	173	5.1%				142	4.2%				35	1.0%			
BP User	1,704	50.0%	43	2.5%	0.31		<0.001	31	1.8%	0.27		<0.001	7	0.4%	0.25		<0.001
BP Non-user	1,704	50.0%	130	7.6%	0.22	0.45		111	6.5%	0.18	0.40		28	1.6%	0.11	0.57	
Age 61-70	5,830	100.0%	267	4.6%				243	4.2%				60	1.0%			
BP User	2,894	49.6%	61	2.1%	0.29		<0.001	51	1.8%	0.26		<0.001	16	0.6%	0.37		<0.001
BP Non-user	2,936	50.4%	206	7.0%	0.21	0.38		192	6.5%	0.19	0.35		44	1.5%	0.21	0.65	
Age 71-80	6,974	100.0%	245	3.5%				243	3.5%				77	1.1%			
BP User	3,508	50.3%	49	1.4%	0.24		<0.001	62	1.8%	0.33		<0.001	22	0.6%	0.39		<0.001
BP Non-user	3,466	49.7%	196	5.7%	0.17	0.32		181	5.2%	0.24	0.44		55	1.6%	0.24	0.64	
Age ≥81	4,637	100.0%	147	3.2%				193	4.2%				63	1.4%			
BP User	2,319	50.0%	26	1.1%	0.21		<0.001	38	1.6%	0.23		<0.001	12	0.5%	0.23		<0.001
BP Non-user	2,318	50.0%	121	5.2%	0.13	0.32		155	6.7%	0.16	0.33		51	2.2%	0.12	0.43	

BP: bisphosphonate; LL: lower 95% confidence interval level; NA: not applicable; OR: odds ratio; UL: upper 95% confidence interval level.

**Appendix 2—table 8.** Unadjusted COVID-19-Related Outcomes Stratified by Age, Sex, & Age by Sex; Matched Primary Analysis Cohort, Region=Midwest.

	Region=Midwest Matched																
	All		SARS-CoV-2 Test					COVID-19 Diagnosis					COVID-19 Hospitalization				
	N	%	N	%	OR		p-value	N	%	OR		p-value	N	%	OR		p-value
					LL	UL				LL	UL				LL	UL	
All Patients	151,802	100.0%	4,451	2.9%				2,099	1.4%				636	0.4%			
BP User	75,901	50.0%	868	1.1%	0.23		<0.001	383	0.5%	0.22		<0.001	121	0.2%	0.23		<0.001
BP Non-user	75,901	50.0%	3,583	4.7%	0.22	0.25		1,716	2.3%	0.20	0.25		515	0.7%	0.19	0.29	
**By Age**																	
Age ≤20	537	100.0%	15	2.8%				2	0.4%				0	0.0%			
BP User	268	49.9%	6	2.2%	0.66		0.44	0	0.0%	NA		NA	0	0.0%	NA		NA
BP Non-user	269	50.1%	9	3.3%	0.23	1.89		2	0.7%	NA	NA		0	0.0%	NA	NA	
Age 21-40	1,188	100.0%	62	5.2%				17	1.4%				1	0.1%			
BP User	593	49.9%	7	1.2%	0.12		<0.001	2	0.3%	0.13		0.002	0	0.0%	NA		NA
BP Non-user	595	50.1%	55	9.2%	0.05	0.26		15	2.5%	0.03	0.57		1	0.2%	NA	NA	
Age 41-50	3,367	100.0%	184	5.5%				46	1.4%				16	0.5%			
BP User	1,684	50.0%	36	2.1%	0.23		<0.001	10	0.6%	0.27		<0.001	2	0.1%	0.14		0.002
BP Non-user	1,683	50.0%	148	8.8%	0.16	0.33		36	2.1%	0.14	0.55		14	0.8%	0.03	0.62	
Age 51-60	24,478	100.0%	951	3.9%				293	1.2%				80	0.3%			
BP User	12,238	50.0%	180	1.5%	0.22		<0.001	52	0.4%	0.21		<0.001	15	0.1%	0.23		<0.001
BP Non-user	12,240	50.0%	771	6.3%	0.19	0.26		241	2.0%	0.16	0.29		65	0.5%	0.13	0.40	
Age 61-70	56,991	100.0%	1,764	3.1%				671	1.2%				189	0.3%			
BP User	28,496	50.0%	322	1.1%	0.21		<0.001	123	0.4%	0.22		<0.001	35	0.1%	0.23		<0.001
BP Non-user	28,495	50.0%	1,442	5.1%	0.19	0.24		548	1.9%	0.18	0.27		154	0.5%	0.16	0.33	
Age 71-80	42,153	100.0%	1,009	2.4%				577	1.4%				200	0.5%			
BP User	21,078	50.0%	209	1.0%	0.25		<0.001	95	0.5%	0.19		<0.001	37	0.2%	0.23		<0.001
BP Non-user	21,075	50.0%	800	3.8%	0.22	0.30		482	2.3%	0.16	0.24		163	0.8%	0.16	0.32	
Age ≥81	23,088	100.0%	466	2.0%				493	2.1%				150	0.6%			
BP User	11,544	50.0%	108	0.9%	0.30		<0.001	101	0.9%	0.25		<0.001	32	0.3%	0.27		<0.001
BP Non-user	11,544	50.0%	358	3.1%	0.24	0.37		392	3.4%	0.20	0.31		118	1.0%	0.18	0.40	
**Female Patients**	**137,028**	**100.0%**	**3,945**	**2.9%**				**1,828**	**1.3%**				**543**	**0.4%**			
BP User	68,512	50.0%	762	1.1%	0.23		<0.001	333	0.5%	0.22		<0.001	103	0.2%	0.23		<0.001
BP Non-user	68,516	50.0%	3,183	4.6%	0.21	0.25		1,495	2.2%	0.19	0.25		440	0.6%	0.19	0.29	
**By Age**																	
Age ≤20	226	100.0%	7	3.1%				1	0.4%				0	0.0%			
BP User	113	50.0%	3	2.7%	0.74		1.00	0	0.0%	NA		NA	0	0.0%	NA		NA
BP Non-user	113	50.0%	4	3.5%	0.16	3.40		1	0.9%	NA	NA		0	0.0%	NA	NA	
Age 21-40	700	100.0%	34	4.9%				7	1.0%				0	0.0%			
BP User	349	49.9%	6	1.7%	0.20		<0.001	1	0.3%	0.17		0.12	0	0.0%	NA		NA
BP Non-user	351	50.1%	28	8.0%	0.08	0.49		6	1.7%	0.02	1.38		0	0.0%	NA	NA	
Age 41-50	2,639	100.0%	157	5.9%				32	1.2%				10	0.4%			
BP User	1,319	50.0%	31	2.4%	0.23		<0.001	8	0.6%	0.33		0.005	1	0.1%	0.11		0.02
BP Non-user	1,320	50.0%	126	9.5%	0.15	0.34		24	1.8%	0.15	0.74		9	0.7%	0.01	0.87	
Age 51-60	22,101	100.0%	856	3.9%				260	1.2%				70	0.3%			
BP User	11,050	50.0%	159	1.4%	0.22		<0.001	47	0.4%	0.22		<0.001	13	0.1%	0.23		<0.001
BP Non-user	11,051	50.0%	697	6.3%	0.18	0.26		213	1.9%	0.16	0.30		57	0.5%	0.12	0.42	
Age 61-70	52,520	100.0%	1,594	3.0%				591	1.1%				165	0.3%			
BP User	26,260	50.0%	286	1.1%	0.21		<0.001	107	0.4%	0.22		<0.001	29	0.1%	0.21		<0.001
BP Non-user	26,260	50.0%	1,308	5.0%	0.18	0.24		484	1.8%	0.18	0.27		136	0.5%	0.14	0.32	
Age 71-80	38,367	100.0%	877	2.3%				501	1.3%				172	0.4%			
BP User	19,184	50.0%	180	0.9%	0.25		<0.001	85	0.4%	0.20		<0.001	33	0.2%	0.24		<0.001
BP Non-user	19,183	50.0%	697	3.6%	0.21	0.30		416	2.2%	0.16	0.25		139	0.7%	0.16	0.35	
Age ≥81	20,475	100.0%	420	2.1%				436	2.1%				126	0.6%			
BP User	10,237	50.0%	97	0.9%	0.29		<0.001	85	0.8%	0.24		<0.001	27	0.3%	0.27		<0.001
BP Non-user	10,238	50.0%	323	3.2%	0.23	0.37		351	3.4%	0.19	0.30		99	1.0%	0.18	0.41	
**Male Patients**	**14,774**	**100.0%**	**506**	**3.4%**				**271**	**1.8%**				**93**	**0.6%**			
BP User	7,389	50.0%	106	1.4%	0.25		<0.001	50	0.7%	0.22		<0.001	18	0.2%	0.24		<0.001
BP Non-user	7,385	50.0%	400	5.4%	0.20	0.32		221	3.0%	0.16	0.30		75	1.0%	0.14	0.40	
**By Age**																	
Age ≤20	311	100.0%	8	2.6%				1	0.3%				0	0.0%			
BP User	155	49.8%	3	1.9%	0.60		0.72	0	0.0%	NA		NA	0	0.0%	NA		NA
BP Non-user	156	50.2%	5	3.2%	0.14	2.54		1	0.6%	NA	NA		0	0.0%	NA	NA	
Age 21-40	488	100.0%	28	5.7%				10	2.0%				1	0.2%			
BP User	244	50.0%	1	0.4%	0.03		<0.001	1	0.4%	0.11		0.02	0	0.0%	NA		NA
BP Non-user	244	50.0%	27	11.1%	0.00	0.25		9	3.7%	0.01	0.85		1	0.4%	NA	NA	
Age 41-50	728	100.0%	27	3.7%				14	1.9%				6	0.8%			
BP User	365	50.1%	5	1.4%	0.22		<0.001	2	0.5%	0.16		0.007	1	0.3%	0.20		0.12
BP Non-user	363	49.9%	22	6.1%	0.08	0.57		12	3.3%	0.04	0.73		5	1.4%	0.02	1.69	
Age 51-60	2,377	100.0%	95	4.0%				33	1.4%				10	0.4%			
BP User	1,188	50.0%	21	1.8%	0.27		<0.001	5	0.4%	0.18		<0.001	2	0.2%	0.25		0.11
BP Non-user	1,189	50.0%	74	6.2%	0.17	0.44		28	2.4%	0.07	0.46		8	0.7%	0.05	1.17	
Age 61-70	4,471	100.0%	170	3.8%				80	1.8%				24	0.5%			
BP User	2,236	50.0%	36	1.6%	0.26		<0.001	16	0.7%	0.24		<0.001	6	0.3%	0.33		0.01
BP Non-user	2,235	50.0%	134	6.0%	0.18	0.37		64	2.9%	0.14	0.42		18	0.8%	0.13	0.84	
Age 71-80	3,786	100.0%	132	3.5%				76	2.0%				28	0.7%			
BP User	1,894	50.0%	29	1.5%	0.27		<0.001	10	0.5%	0.15		<0.001	4	0.2%	0.16		<0.001
BP Non-user	1,892	50.0%	103	5.4%	0.18	0.41		66	3.5%	0.08	0.29		24	1.3%	0.06	0.48	
Age ≥81	2,613	100.0%	46	1.8%				57	2.2%				24	0.9%			
BP User	1,307	50.0%	11	0.8%	0.31		<0.001	16	1.2%	0.38		<0.001	5	0.4%	0.26		0.004
BP Non-user	1,306	50.0%	35	2.7%	0.16	0.61		41	3.1%	0.21	0.69		19	1.5%	0.10	0.70	

BP: bisphosphonate; LL: lower 95% confidence interval level; NA: not applicable; OR: odds ratio; UL: upper 95% confidence interval level.

**Appendix 2—table 9.** Unadjusted COVID-19-Related Outcomes Stratified by Age, Sex, & Age by Sex; Matched Primary Analysis Cohort, Region=South.

	Region=South Matched																
	All		SARS-CoV-2 Test					COVID-19 Diagnosis					COVID-19 Hospitalization				
	N	%	N	%	OR		p-value	N	%	OR		p-value	N	%	OR		p-value
					LL	UL				LL	UL				LL	UL	
All Patients	319,408	100.0%	8,418	2.6%				3,535	1.1%				849	0.3%			
BP User	159,704	50.0%	1,553	1.0%	0.22		<0.001	624	0.4%	0.21		<0.001	167	0.1%	0.24		<0.001
BP Non-user	159,704	50.0%	6,865	4.3%	0.21	0.23		2,911	1.8%	0.19	0.23		682	0.4%	0.21	0.29	
**By Age**																	
Age ≤20	943	100.0%	29	3.1%				4	0.4%				0	0.0%			
BP User	474	50.3%	15	3.2%	1.06		0.87	0	0.0%	NA		NA	0	0.0%	NA		NA
BP Non-user	469	49.7%	14	3.0%	0.51	2.23		4	0.9%	NA	NA		0	0.0%	NA	NA	
Age 21-40	2,364	100.0%	113	4.8%				25	1.1%				4	0.2%			
BP User	1,177	49.8%	20	1.7%	0.20		<0.001	4	0.3%	0.19		<0.001	2	0.2%	1.01		1.00
BP Non-user	1,187	50.2%	93	7.8%	0.12	0.33		21	1.8%	0.06	0.55		2	0.2%	0.14	7.17	
Age 41-50	6,839	100.0%	329	4.8%				73	1.1%				10	0.1%			
BP User	3,421	50.0%	72	2.1%	0.26		<0.001	18	0.5%	0.32		<0.001	5	0.1%	1.00		0.99
BP Non-user	3,418	50.0%	257	7.5%	0.20	0.34		55	1.6%	0.19	0.55		5	0.1%	0.29	3.45	
Age 51-60	50,186	100.0%	1,999	4.0%				584	1.2%				103	0.2%			
BP User	25,093	50.0%	393	1.6%	0.23		<0.001	114	0.5%	0.24		<0.001	23	0.1%	0.29		<0.001
BP Non-user	25,093	50.0%	1,606	6.4%	0.21	0.26		470	1.9%	0.19	0.29		80	0.3%	0.18	0.46	
Age 61-70	111,800	100.0%	3,246	2.9%				1,106	1.0%				247	0.2%			
BP User	55,896	50.0%	583	1.0%	0.21		<0.001	191	0.3%	0.21		<0.001	38	0.1%	0.18		<0.001
BP Non-user	55,904	50.0%	2,663	4.8%	0.19	0.23		915	1.6%	0.18	0.24		209	0.4%	0.13	0.26	
Age 71-80	99,223	100.0%	1,942	2.0%				1,029	1.0%				260	0.3%			
BP User	49,618	50.0%	322	0.6%	0.19		<0.001	170	0.3%	0.20		<0.001	55	0.1%	0.27		<0.001
BP Non-user	49,605	50.0%	1,620	3.3%	0.17	0.22		859	1.7%	0.17	0.23		205	0.4%	0.20	0.36	
Age ≥81	48,053	100.0%	760	1.6%				714	1.5%				225	0.5%			
BP User	24,025	50.0%	148	0.6%	0.24		<0.001	127	0.5%	0.21		<0.001	44	0.2%	0.24		<0.001
BP Non-user	24,028	50.0%	612	2.5%	0.20	0.28		587	2.4%	0.18	0.26		181	0.8%	0.17	0.34	
**Female Patients**	**289,263**	**100.0%**	**7,519**	**2.6%**				**3,159**	**1.1%**				**745**	**0.3%**			
BP User	144,633	50.0%	1,365	0.9%	0.21		<0.001	562	0.4%	0.21		<0.001	143	0.1%	0.24		<0.001
BP Non-user	144,630	50.0%	6,154	4.3%	0.20	0.23		2,597	1.8%	0.19	0.23		602	0.4%	0.20	0.28	
**By Age**																	
Age ≤20	372	100.0%	11	3.0%				3	0.8%				0	0.0%			
BP User	185	49.7%	6	3.2%	1.22		0.75	0	0.0%	NA		NA	0	0.0%	NA		NA
BP Non-user	187	50.3%	5	2.7%	0.37	4.07		3	1.6%	NA	NA		0	0.0%	NA	NA	
Age 21-40	1,543	100.0%	81	5.2%				16	1.0%				3	0.2%			
BP User	770	49.9%	14	1.8%	0.20		<0.001	4	0.5%	0.33		0.08	2	0.3%	2.01		0.62
BP Non-user	773	50.1%	67	8.7%	0.11	0.35		12	1.6%	0.11	1.03		1	0.1%	0.18	22.22	
Age 41-50	5,569	100.0%	273	4.9%				66	1.2%				9	0.2%			
BP User	2,787	50.0%	65	2.3%	0.30		<0.001	18	0.6%	0.37		<0.001	5	0.2%	1.25		1.00
BP Non-user	2,782	50.0%	208	7.5%	0.22	0.39		48	1.7%	0.21	0.64		4	0.1%	0.33	4.65	
Age 51-60	46,012	100.0%	1,819	4.0%				521	1.1%				89	0.2%			
BP User	23,007	50.0%	358	1.6%	0.23		<0.001	100	0.4%	0.23		<0.001	16	0.1%	0.22		<0.001
BP Non-user	23,005	50.0%	1,461	6.4%	0.21	0.26		421	1.8%	0.19	0.29		73	0.3%	0.13	0.38	
Age 61-70	103,825	100.0%	2,948	2.8%				1,007	1.0%				218	0.2%			
BP User	51,910	50.0%	517	1.0%	0.20		<0.001	177	0.3%	0.21		<0.001	33	0.1%	0.18		<0.001
BP Non-user	51,915	50.0%	2,431	4.7%	0.19	0.23		830	1.6%	0.18	0.25		185	0.4%	0.12	0.26	
Age 71-80	89,474	100.0%	1,729	1.9%				915	1.0%				230	0.3%			
BP User	44,742	50.0%	283	0.6%	0.19		<0.001	153	0.3%	0.20		<0.001	47	0.1%	0.26		<0.001
BP Non-user	44,732	50.0%	1,446	3.2%	0.17	0.22		762	1.7%	0.17	0.24		183	0.4%	0.19	0.35	
Age ≥81	42,468	100.0%	658	1.5%				631	1.5%				196	0.5%			
BP User	21,232	50.0%	122	0.6%	0.22		<0.001	110	0.5%	0.21		<0.001	40	0.2%	0.26		<0.001
BP Non-user	21,236	50.0%	536	2.5%	0.18	0.27		521	2.5%	0.17	0.25		156	0.7%	0.18	0.36	
**Male Patients**	**30,145**	**100.0%**	**899**	**3.0%**				**376**	**1.2%**				**104**	**0.3%**			
BP User	15,071	50.0%	188	1.2%	0.26		<0.001	62	0.4%	0.19		<0.001	24	0.2%	0.30		<0.001
BP Non-user	15,074	50.0%	711	4.7%	0.22	0.30		314	2.1%	0.15	0.26		80	0.5%	0.19	0.47	
**By Age**																	
Age ≤20	571	100.0%	18	3.2%				1	0.2%				0	0.0%			
BP User	289	50.6%	9	3.1%	0.98		0.96	0	0.0%	NA		NA	0	0.0%	NA		NA
BP Non-user	282	49.4%	9	3.2%	0.38	2.49		1	0.4%	NA	NA		0	0.0%	NA	NA	
Age 21-40	821	100.0%	32	3.9%				9	1.1%				1	0.1%			
BP User	407	49.6%	6	1.5%	0.22		<0.001	0	0.0%	NA		NA	0	0.0%	NA		NA
BP Non-user	414	50.4%	26	6.3%	0.09	0.55		9	2.2%	NA	NA		1	0.2%	NA	NA	
Age 41-50	1,270	100.0%	56	4.4%				7	0.6%				1	0.1%			
BP User	634	49.9%	7	1.1%	0.13		<0.001	0	0.0%	NA		NA	0	0.0%	NA		NA
BP Non-user	636	50.1%	49	7.7%	0.06	0.30		7	1.1%	NA	NA		1	0.2%	NA	NA	
Age 51-60	4,174	100.0%	180	4.3%				63	1.5%				14	0.3%			
BP User	2,086	50.0%	35	1.7%	0.23		<0.001	14	0.7%	0.28		<0.001	7	0.3%	1.00		0.99
BP Non-user	2,088	50.0%	145	6.9%	0.16	0.33		49	2.3%	0.15	0.51		7	0.3%	0.35	2.86	
Age 61-70	7,975	100.0%	298	3.7%				99	1.2%				29	0.4%			
BP User	3,986	50.0%	66	1.7%	0.27		<0.001	14	0.4%	0.16		<0.001	5	0.1%	0.21		<0.001
BP Non-user	3,989	50.0%	232	5.8%	0.21	0.36		85	2.1%	0.09	0.29		24	0.6%	0.08	0.54	
Age 71-80	9,749	100.0%	213	2.2%				114	1.2%				30	0.3%			
BP User	4,876	50.0%	39	0.8%	0.22		<0.001	17	0.3%	0.17		<0.001	8	0.2%	0.36		0.01
BP Non-user	4,873	50.0%	174	3.6%	0.15	0.31		97	2.0%	0.10	0.29		22	0.5%	0.16	0.81	
Age ≥81	5,585	100.0%	102	1.8%				83	1.5%				29	0.5%			
BP User	2,793	50.0%	26	0.9%	0.34		<0.001	17	0.6%	0.25		<0.001	4	0.1%	0.16		<0.001
BP Non-user	2,792	50.0%	76	2.7%	0.21	0.53		66	2.4%	0.15	0.43		25	0.9%	0.06	0.46	

BP: bisphosphonate; LL: lower 95% confidence interval level; NA: not applicable; OR: odds ratio; UL: upper 95% confidence interval level.

**Appendix 2—table 10.** Unadjusted COVID-19-Related Outcomes Stratified by Age, Sex, & Age by Sex; Matched Primary Analysis Cohort, Region=West.

	Region=West Matched																
	All		SARS-CoV-2 Test					COVID-19 Diagnosis					COVID-19 Hospitalization				
	N	%	N	%	OR		p-value	N	%	OR		p-value	N	%	OR		p-value
					LL	UL				LL	UL				LL	UL	
All Patients	190,534	100.0%	6,437	3.4%				2,835	1.5%				720	0.4%			
BP User	95,267	50.0%	1,084	1.1%	0.19		<0.001	439	0.5%	0.18		<0.001	113	0.1%	0.19		<0.001
BP Non-user	95,267	50.0%	5,353	5.6%	0.18	0.21		2,396	2.5%	0.16	0.20		607	0.6%	0.15	0.23	
**By Age**																	
Age ≤20	299	100.0%	9	3.0%				1	0.3%				0	0.0%			
BP User	150	50.2%	1	0.7%	0.12		0.02	0	0.0%	NA		NA	0	0.0%	NA		NA
BP Non-user	149	49.8%	8	5.4%	0.01	0.96		1	0.7%	NA	NA		0	0.0%	NA	NA	
Age 21-40	1,115	100.0%	67	6.0%				18	1.6%				3	0.3%			
BP User	557	50.0%	17	3.1%	0.32		<0.001	2	0.4%	0.12		0.001	1	0.2%	0.50		1.00
BP Non-user	558	50.0%	50	9.0%	0.18	0.56		16	2.9%	0.03	0.53		2	0.4%	0.05	5.53	
Age 41-50	2,806	100.0%	147	5.2%				33	1.2%				10	0.4%			
BP User	1,404	50.0%	27	1.9%	0.21		<0.001	3	0.2%	0.10		<0.001	1	0.1%	0.11		0.01
BP Non-user	1,402	50.0%	120	8.6%	0.14	0.32		30	2.1%	0.03	0.32		9	0.6%	0.01	0.87	
Age 51-60	20,761	100.0%	952	4.6%				304	1.5%				54	0.3%			
BP User	10,380	50.0%	178	1.7%	0.22		<0.001	53	0.5%	0.21		<0.001	7	0.1%	0.15		<0.001
BP Non-user	10,381	50.0%	774	7.5%	0.18	0.26		251	2.4%	0.15	0.28		47	0.5%	0.07	0.33	
Age 61-70	62,798	100.0%	2,228	3.5%				828	1.3%				196	0.3%			
BP User	31,392	50.0%	341	1.1%	0.17		<0.001	118	0.4%	0.16		<0.001	21	0.1%	0.12		<0.001
BP Non-user	31,406	50.0%	1,887	6.0%	0.15	0.19		710	2.3%	0.13	0.20		175	0.6%	0.08	0.19	
Age 71-80	64,596	100.0%	1,962	3.0%				915	1.4%				259	0.4%			
BP User	32,303	50.0%	336	1.0%	0.20		<0.001	137	0.4%	0.17		<0.001	43	0.1%	0.20		<0.001
BP Non-user	32,293	50.0%	1,626	5.0%	0.18	0.22		778	2.4%	0.14	0.21		216	0.7%	0.14	0.27	
Age ≥81	38,159	100.0%	1,072	2.8%				736	1.9%				198	0.5%			
BP User	19,081	50.0%	184	1.0%	0.20		<0.001	126	0.7%	0.20		<0.001	40	0.2%	0.25		<0.001
BP Non-user	19,078	50.0%	888	4.7%	0.17	0.23		610	3.2%	0.17	0.24		158	0.8%	0.18	0.36	
**Female Patients**	**168,933**	**100.0%**	**5,575**	**3.3%**				**2,439**	**1.4%**				**576**	**0.3%**			
BP User	84,470	50.0%	909	1.1%	0.19		<0.001	380	0.4%	0.18		<0.001	92	0.1%	0.19		<0.001
BP Non-user	84,463	50.0%	4,666	5.5%	0.17	0.20		2,059	2.4%	0.16	0.20		484	0.6%	0.15	0.24	
**By Age**																	
Age ≤20	107	100.0%	4	3.7%				0	0.0%				0	0.0%			
BP User	54	50.5%	0	0.0%	NA		NA	0	0.0%	NA		NA	0	0.0%	NA		NA
BP Non-user	53	49.5%	4	7.5%	NA	NA		0	0.0%	NA	NA		0	0.0%	NA	NA	
Age 21-40	658	100.0%	44	6.7%				9	1.4%				2	0.3%			
BP User	329	50.0%	10	3.0%	0.27		<0.001	1	0.3%	0.12		0.04	0	0.0%	NA		NA
BP Non-user	329	50.0%	34	10.3%	0.13	0.56		8	2.4%	0.02	0.98		2	0.6%	NA	NA	
Age 41-50	2,158	100.0%	124	5.7%				21	1.0%				5	0.2%			
BP User	1,080	50.0%	21	1.9%	0.19		<0.001	2	0.2%	0.10		<0.001	0	0.0%	NA		NA
BP Non-user	1,078	50.0%	103	9.6%	0.12	0.30		19	1.8%	0.02	0.45		5	0.5%	NA	NA	
Age 51-60	18,480	100.0%	835	4.5%				264	1.4%				43	0.2%			
BP User	9,239	50.0%	146	1.6%	0.20		<0.001	46	0.5%	0.21		<0.001	4	0.0%	0.10		<0.001
BP Non-user	9,241	50.0%	689	7.5%	0.17	0.24		218	2.4%	0.15	0.29		39	0.4%	0.04	0.29	
Age 61-70	57,528	100.0%	1,999	3.5%				727	1.3%				159	0.3%			
BP User	28,761	50.0%	300	1.0%	0.17		<0.001	111	0.4%	0.18		<0.001	19	0.1%	0.14		<0.001
BP Non-user	28,767	50.0%	1,699	5.9%	0.15	0.19		616	2.1%	0.14	0.22		140	0.5%	0.08	0.22	
Age 71-80	57,396	100.0%	1,677	2.9%				775	1.4%				208	0.4%			
BP User	28,703	50.0%	284	1.0%	0.20		<0.001	118	0.4%	0.18		<0.001	36	0.1%	0.21		<0.001
BP Non-user	28,693	50.0%	1,393	4.9%	0.17	0.22		657	2.3%	0.14	0.21		172	0.6%	0.15	0.30	
Age ≥81	32,606	100.0%	892	2.7%				643	2.0%				159	0.5%			
BP User	16,304	50.0%	148	0.9%	0.19		<0.001	102	0.6%	0.18		<0.001	33	0.2%	0.26		<0.001
BP Non-user	16,302	50.0%	744	4.6%	0.16	0.23		541	3.3%	0.15	0.23		126	0.8%	0.18	0.38	
**Male Patients**	**21,601**	**100.0%**	**862**	**4.0%**				**396**	**1.8%**				**144**	**0.7%**			
BP User	10,797	50.0%	175	1.6%	0.24		<0.001	59	0.5%	0.17		<0.001	21	0.2%	0.17		<0.001
BP Non-user	10,804	50.0%	687	6.4%	0.21	0.29		337	3.1%	0.13	0.23		123	1.1%	0.11	0.27	
**By Age**																	
Age ≤20	192	100.0%	5	2.6%				1	0.5%				0	0.0%			
BP User	96	50.0%	1	1.0%	0.24		0.37	0	0.0%	NA		NA	0	0.0%	NA		NA
BP Non-user	96	50.0%	4	4.2%	0.03	2.21		1	1.0%	NA	NA		0	0.0%	NA	NA	
Age 21-40	457	100.0%	23	5.0%				9	2.0%				1	0.2%			
BP User	228	49.9%	7	3.1%	0.42		0.06	1	0.4%	0.12		0.04	1	0.4%	NA		NA
BP Non-user	229	50.1%	16	7.0%	0.17	1.05		8	3.5%	0.02	0.98		0	0.0%	NA	NA	
Age 41-50	648	100.0%	23	3.5%				12	1.9%				5	0.8%			
BP User	324	50.0%	6	1.9%	0.34		0.02	1	0.3%	0.09		0.006	1	0.3%	0.25		0.37
BP Non-user	324	50.0%	17	5.2%	0.13	0.88		11	3.4%	0.01	0.69		4	1.2%	0.03	2.23	
Age 51-60	2,281	100.0%	117	5.1%				40	1.8%				11	0.5%			
BP User	1,141	50.0%	32	2.8%	0.36		<0.001	7	0.6%	0.21		<0.001	3	0.3%	0.37		0.15
BP Non-user	1,140	50.0%	85	7.5%	0.24	0.54		33	2.9%	0.09	0.47		8	0.7%	0.10	1.41	
Age 61-70	5,270	100.0%	229	4.3%				101	1.9%				37	0.7%			
BP User	2,631	49.9%	41	1.6%	0.21		<0.001	7	0.3%	0.07		<0.001	2	0.1%	0.06		<0.001
BP Non-user	2,639	50.1%	188	7.1%	0.15	0.29		94	3.6%	0.03	0.16		35	1.3%	0.01	0.24	
Age 71-80	7,200	100.0%	285	4.0%				140	1.9%				51	0.7%			
BP User	3,600	50.0%	52	1.4%	0.21		<0.001	19	0.5%	0.15		<0.001	7	0.2%	0.16		<0.001
BP Non-user	3,600	50.0%	233	6.5%	0.16	0.29		121	3.4%	0.09	0.25		44	1.2%	0.07	0.35	
Age ≥81	5,553	100.0%	180	3.2%				93	1.7%				39	0.7%			
BP User	2,777	50.0%	36	1.3%	0.24		<0.001	24	0.9%	0.34		<0.001	7	0.3%	0.22		<0.001
BP Non-user	2,776	50.0%	144	5.2%	0.17	0.35		69	2.5%	0.21	0.55		32	1.2%	0.10	0.49	

BP: bisphosphonate; LL: lower 95% confidence interval level; NA: not applicable; OR: odds ratio; UL: upper 95% confidence interval level.

**Appendix 2—table 11.** Unadjusted COVID-19-Related Outcomes Stratified by Age, Sex, & Age by Sex; Matched Primary Analysis Cohort, Region=New York State.

	Region=New York State Matched																
	All		SARS-CoV-2 Test					COVID-19 Diagnosis					COVID-19 Hospitalization				
	N	%	N	%	OR		p-value	N	%	OR		p-value	N	%	OR		p-value
					LL	UL				LL	UL				LL	UL	
All Patients	99,724	100.0%	3,598	3.6%				3,607	3.6%				622	0.6%			
BP User	49,862	50.0%	772	1.5%	0.26		<0.001	811	1.6%	0.28		<0.001	136	0.3%	0.28		<0.001
BP Non-user	49,862	50.0%	2,826	5.7%	0.24	0.28		2,796	5.6%	0.26	0.30		486	1.0%	0.23	0.34	
**By Age**																	
Age ≤20	102	100.0%	4	3.9%				2	2.0%				1	1.0%			
BP User	50	49.0%	2	4.0%	1.04		1.00	1	2.0%	1.04		1.00	1	2.0%	NA		NA
BP Non-user	52	51.0%	2	3.8%	0.14	7.69		1	1.9%	0.06	17.11		0	0.0%	NA	NA	
Age 21-40	453	100.0%	21	4.6%				15	3.3%				1	0.2%			
BP User	228	50.3%	3	1.3%	0.15		<0.001	2	0.9%	0.14		0.004	1	0.4%	NA		NA
BP Non-user	225	49.7%	18	8.0%	0.04	0.53		13	5.8%	0.03	0.65		0	0.0%	NA	NA	
Age 41-50	1,311	100.0%	77	5.9%				36	2.7%				4	0.3%			
BP User	655	50.0%	22	3.4%	0.38		<0.001	8	1.2%	0.28		<0.001	1	0.2%	0.33		0.62
BP Non-user	656	50.0%	55	8.4%	0.23	0.63		28	4.3%	0.13	0.61		3	0.5%	0.03	3.21	
Age 51-60	12,732	100.0%	688	5.4%				527	4.1%				58	0.5%			
BP User	6,364	50.0%	155	2.4%	0.27		<0.001	118	1.9%	0.28		<0.001	17	0.3%	0.41		0.002
BP Non-user	6,368	50.0%	533	8.4%	0.23	0.33		409	6.4%	0.22	0.34		41	0.6%	0.23	0.73	
Age 61-70	32,265	100.0%	1,294	4.0%				1,150	3.6%				141	0.4%			
BP User	16,136	50.0%	277	1.7%	0.26		<0.001	267	1.7%	0.29		<0.001	27	0.2%	0.24		<0.001
BP Non-user	16,129	50.0%	1,017	6.3%	0.23	0.30		883	5.5%	0.25	0.33		114	0.7%	0.15	0.36	
Age 71-80	34,693	100.0%	957	2.8%				1,196	3.4%				240	0.7%			
BP User	17,341	50.0%	204	1.2%	0.26		<0.001	257	1.5%	0.26		<0.001	45	0.3%	0.23		<0.001
BP Non-user	17,352	50.0%	753	4.3%	0.22	0.31		939	5.4%	0.23	0.30		195	1.1%	0.17	0.32	
Age ≥81	18,168	100.0%	557	3.1%				681	3.7%				177	1.0%			
BP User	9,088	50.0%	109	1.2%	0.23		<0.001	158	1.7%	0.29		<0.001	44	0.5%	0.33		<0.001
BP Non-user	9,080	50.0%	448	4.9%	0.19	0.29		523	5.8%	0.24	0.35		133	1.5%	0.23	0.46	
**Female Patients**	**90,567**	**100.0%**	**3,255**	**3.6%**				**3,235**	**3.6%**				**537**	**0.6%**			
BP User	45,285	50.0%	687	1.5%	0.26		<0.001	726	1.6%	0.28		<0.001	108	0.2%	0.25		<0.001
BP Non-user	45,282	50.0%	2,568	5.7%	0.24	0.28		2,509	5.5%	0.26	0.30		429	0.9%	0.20	0.31	
**By Age**																	
Age ≤20	33	100.0%	0	0.0%				1	3.0%				1	3.0%			
BP User	16	48.5%	0	0.0%	NA		NA	1	6.3%	NA		NA	1	6.3%	NA		NA
BP Non-user	17	51.5%	0	0.0%	NA	NA		0	0.0%	NA	NA		0	0.0%	NA	NA	
Age 21-40	261	100.0%	16	6.1%				8	3.1%				1	0.4%			
BP User	132	50.6%	2	1.5%	0.13		0.002	2	1.5%	0.32		0.17	1	0.8%	NA		NA
BP Non-user	129	49.4%	14	10.9%	0.03	0.57		6	4.7%	0.06	1.59		0	0.0%	NA	NA	
Age 41-50	1,032	100.0%	58	5.6%				28	2.7%				3	0.3%			
BP User	516	50.0%	18	3.5%	0.43		0.003	7	1.4%	0.32		0.007	0	0.0%	NA		NA
BP Non-user	516	50.0%	40	7.8%	0.24	0.76		21	4.1%	0.14	0.77		3	0.6%	NA	NA	
Age 51-60	11,699	100.0%	637	5.4%				482	4.1%				47	0.4%			
BP User	5,849	50.0%	138	2.4%	0.26		<0.001	110	1.9%	0.28		<0.001	14	0.2%	0.42		0.006
BP Non-user	5,850	50.0%	499	8.5%	0.21	0.31		372	6.4%	0.23	0.35		33	0.6%	0.23	0.79	
Age 61-70	30,115	100.0%	1,204	4.0%				1,070	3.6%				126	0.4%			
BP User	15,060	50.0%	257	1.7%	0.26		<0.001	248	1.6%	0.29		<0.001	23	0.2%	0.22		<0.001
BP Non-user	15,055	50.0%	947	6.3%	0.22	0.30		822	5.5%	0.25	0.33		103	0.7%	0.14	0.35	
Age 71-80	31,385	100.0%	858	2.7%				1,052	3.4%				208	0.7%			
BP User	15,688	50.0%	176	1.1%	0.25		<0.001	221	1.4%	0.26		<0.001	33	0.2%	0.19		<0.001
BP Non-user	15,697	50.0%	682	4.3%	0.21	0.30		831	5.3%	0.22	0.30		175	1.1%	0.13	0.27	
Age ≥81	16,042	100.0%	482	3.0%				594	3.7%				151	0.9%			
BP User	8,024	50.0%	96	1.2%	0.24		<0.001	137	1.7%	0.29		<0.001	36	0.4%	0.31		<0.001
BP Non-user	8,018	50.0%	386	4.8%	0.19	0.30		457	5.7%	0.24	0.35		115	1.4%	0.21	0.45	
**Male Patients**	**9,157**	**100.0%**	**343**	**3.7%**				**372**	**4.1%**				**85**	**0.9%**			
BP User	4,577	50.0%	85	1.9%	0.32		<0.001	85	1.9%	0.28		<0.001	28	0.6%	0.49		0.002
BP Non-user	4,580	50.0%	258	5.6%	0.25	0.41		287	6.3%	0.22	0.36		57	1.2%	0.31	0.77	
**By Age**																	
Age ≤20	69	100.0%	4	5.8%				1	1.4%				0	0.0%			
BP User	34	49.3%	2	5.9%	1.03		1.00	0	0.0%	NA		NA	0	0.0%	NA		NA
BP Non-user	35	50.7%	2	5.7%	0.14	7.77		1	2.9%	NA	NA		0	0.0%	NA	NA	
Age 21-40	192	100.0%	5	2.6%				7	3.6%				0	0.0%			
BP User	96	50.0%	1	1.0%	0.24		0.37	0	0.0%	NA		NA	0	0.0%	NA		NA
BP Non-user	96	50.0%	4	4.2%	0.03	2.21		7	7.3%	NA	NA		0	0.0%	NA	NA	
Age 41-50	279	100.0%	19	6.8%				8	2.9%				1	0.4%			
BP User	139	49.8%	4	2.9%	0.25		0.02	1	0.7%	0.14		0.07	1	0.7%	NA		NA
BP Non-user	140	50.2%	15	10.7%	0.08	0.76		7	5.0%	0.02	1.13		0	0.0%	NA	NA	
Age 51-60	1,033	100.0%	51	4.9%				45	4.4%				11	1.1%			
BP User	515	49.9%	17	3.3%	0.49		0.02	8	1.6%	0.21		<0.001	3	0.6%	0.37		0.22
BP Non-user	518	50.1%	34	6.6%	0.27	0.88		37	7.1%	0.09	0.44		8	1.5%	0.10	1.42	
Age 61-70	2,150	100.0%	90	4.2%				80	3.7%				15	0.7%			
BP User	1,076	50.0%	20	1.9%	0.27		<0.001	19	1.8%	0.30		<0.001	4	0.4%	0.36		0.08
BP Non-user	1,074	50.0%	70	6.5%	0.16	0.45		61	5.7%	0.18	0.50		11	1.0%	0.11	1.14	
Age 71-80	3,308	100.0%	99	3.0%				144	4.4%				32	1.0%			
BP User	1,653	50.0%	28	1.7%	0.38		<0.001	36	2.2%	0.32		<0.001	12	0.7%	0.60		0.16
BP Non-user	1,655	50.0%	71	4.3%	0.25	0.60		108	6.5%	0.22	0.47		20	1.2%	0.29	1.23	
Age ≥81	2,126	100.0%	75	3.5%				87	4.1%				26	1.2%			
BP User	1,064	50.0%	13	1.2%	0.20		<0.001	21	2.0%	0.30		<0.001	8	0.8%	0.44		0.05
BP Non-user	1,062	50.0%	62	5.8%	0.11	0.37		66	6.2%	0.18	0.50		18	1.7%	0.19	1.02	

BP: bisphosphonate; LL: lower 95% confidence interval level; NA: not applicable; OR: odds ratio; UL: upper 95% confidence interval level.

**Appendix 2—table 12.** Unadjusted COVID-19-Related Outcomes Stratified by Age, Sex, & Age by Sex; Matched Primary Analysis Cohort, Region=New York State.

		SARS-CoV-2 Test				COVID-19 Diagnosis				COVID-19 Hospitalization			
		**OR**	**LL**	**UL**	**p value**	**OR**	**LL**	**UL**	**p value**	**OR**	**LL**	**UL**	**p value**
**All**	**Unadjusted**	0.22	0.21	0.22	<0.001	0.22	0.21	0.23	<0.001	0.24	0.22	0.26	<0.001
	**Adjusted**	0.22	0.21	0.23	<0.001	0.23	0.22	0.24	<0.001	0.26	0.24	0.29	<0.001
**Northeast**	**Unadjusted**	0.22	0.21	0.24	<0.001	0.24	0.23	0.26	<0.001	0.26	0.23	0.30	<0.001
	**Adjusted**	0.23	0.21	0.24	<0.001	0.25	0.23	0.26	<0.001	0.29	0.26	0.33	<0.001
**Midwest**	**Unadjusted**	0.23	0.22	0.25	<0.001	0.22	0.20	0.25	<0.001	0.23	0.19	0.29	<0.001
	**Adjusted**	0.24	0.22	0.26	<0.001	0.24	0.22	0.27	<0.001	0.26	0.21	0.32	<0.001
**South**	**Unadjusted**	0.22	0.21	0.23	<0.001	0.21	0.19	0.23	<0.001	0.24	0.21	0.29	<0.001
	**Adjusted**	0.22	0.21	0.23	<0.001	0.22	0.20	0.24	<0.001	0.26	0.23	0.30	<0.001
**West**	**Unadjusted**	0.19	0.18	0.21	<0.001	0.18	0.16	0.20	<0.001	0.19	0.15	0.23	<0.001
	**Adjusted**	0.20	0.18	0.21	<0.001	0.19	0.17	0.21	<0.001	0.20	0.16	0.25	<0.001
**New York**	**Unadjusted**	0.26	0.24	0.28	<0.001	0.28	0.26	0.30	<0.001	0.28	0.23	0.34	<0.001
	**Adjusted**	0.26	0.24	0.28	<0.001	0.28	0.26	0.31	<0.001	0.33	0.27	0.40	<0.001

LL: lower 95% confidence interval level; OR: odds ratio; UL: upper 95% confidence interval level.

**Appendix 2—table 13.** Statin Use Sensitivity Analysis, Unadjusted/Adjusted Odds Ratio for COVID-19-Related Outcomes, Stratified by Region and New York State.

		SARS-CoV-2 Test				COVID-19 Diagnosis				COVID-19 Hospitalization			
		**Statin Uses versus Non-users**											
		**OR**	**LL**	**UL**	**p value**	**OR**	**LL**	**UL**	**p value**	**OR**	**LL**	**UL**	**p value**
**All**	**Unadjusted**	0.90	0.89	0.91	<0.001	0.91	0.90	0.92	<0.001	1.12	1.09	1.15	<0.001
	**Adjusted**	0.87	0.86	0.87	<0.001	0.79	0.78	0.81	<0.001	0.99	0.96	1.02	0.48
**Northeast**	**Unadjusted**	0.87	0.85	0.88	<0.001	0.88	0.86	0.90	<0.001	1.16	1.11	1.21	<0.001
	**Adjusted**	0.85	0.84	0.87	<0.001	0.77	0.75	0.78	<0.001	1.03	0.98	1.07	0.22
**Midwest**	**Unadjusted**	0.97	0.95	0.99	0.02	1.10	1.07	1.14	<0.001	1.27	1.19	1.36	<0.001
	**Adjusted**	0.92	0.90	0.94	<0.001	0.99	0.96	1.03	0.75	1.15	1.08	1.23	<0.001
**South**	**Unadjusted**	0.90	0.88	0.91	<0.001	0.90	0.88	0.93	<0.001	1.00	0.95	1.06	0.90
	**Adjusted**	0.85	0.84	0.87	<0.001	0.80	0.78	0.83	<0.001	0.88	0.83	0.94	<0.001
**West**	**Unadjusted**	0.88	0.86	0.90	<0.001	0.83	0.80	0.86	<0.001	1.02	0.95	1.10	0.58
	**Adjusted**	0.86	0.83	0.88	<0.001	0.71	0.68	0.74	<0.001	0.87	0.80	0.94	<0.001
**New York**	**Unadjusted**	0.91	0.89	0.93	<0.001	0.93	0.91	0.96	<0.001	1.21	1.14	1.29	<0.001
	**Adjusted**	0.92	0.90	0.95	<0.001	0.79	0.77	0.82	<0.001	1.05	0.98	1.13	0.15
		**BP Users versus BP Non-users among Statin Users**											
		**OR**	**LL**	**UL**	**p value**	**OR**	**LL**	**UL**	**p value**	**OR**	**LL**	**UL**	**p value**
**All**	**Unadjusted**	0.23	0.22	0.24	<0.001	0.26	0.25	0.28	<0.001	0.26	0.23	0.29	<0.001
	**Adjusted**	0.23	0.22	0.24	<0.001	0.27	0.25	0.29	<0.001	0.28	0.25	0.32	<0.001
**Northeast**	**Unadjusted**	0.25	0.23	0.27	<0.001	0.29	0.27	0.31	<0.001	0.28	0.24	0.34	<0.001
	**Adjusted**	0.25	0.23	0.27	<0.001	0.29	0.27	0.32	<0.001	0.32	0.26	0.38	<0.001
**Midwest**	**Unadjusted**	0.24	0.22	0.27	<0.001	0.22	0.19	0.25	<0.001	0.21	0.16	0.27	<0.001
	**Adjusted**	0.25	0.23	0.29	<0.001	0.23	0.22	0.25	<0.001	0.22	0.17	0.30	<0.001
**South**	**Unadjusted**	0.22	0.21	0.24	<0.001	0.26	0.23	0.29	<0.001	0.26	0.21	0.33	<0.001
	**Adjusted**	0.22	0.20	0.24	<0.001	0.27	0.24	0.31	<0.001	0.28	0.22	0.36	<0.001
**West**	**Unadjusted**	0.20	0.18	0.22	<0.001	0.22	0.19	0.25	<0.001	0.25	0.20	0.33	<0.001
	**Adjusted**	0.20	0.18	0.22	<0.001	0.23	0.20	0.27	<0.001	0.28	0.21	0.36	<0.001
**New York**	**Unadjusted**	0.27	0.24	0.30	<0.001	0.31	0.28	0.35	<0.001	0.30	0.23	0.39	<0.001
	**Adjusted**	0.28	0.25	0.32	<0.001	0.31	0.28	0.35	<0.001	0.33	0.25	0.44	<0.001
		**BP Users versus BP Non-users among Statin Non-users**											
		**OR**	**LL**	**UL**	**p value**	**OR**	**LL**	**UL**	**p value**	**OR**	**LL**	**UL**	**p value**
**All**	**Unadjusted**	0.23	0.21	0.24	<0.001	0.21	0.19	0.23	<0.001	0.21	0.17	0.25	<0.001
	**Adjusted**	0.24	0.22	0.25	<0.001	0.23	0.21	0.25	<0.001	0.25	0.21	0.30	<0.001
**Northeast**	**Unadjusted**	0.25	0.22	0.27	<0.001	0.22	0.20	0.25	<0.001	0.24	0.19	0.31	<0.001
	**Adjusted**	0.26	0.23	0.29	<0.001	0.25	0.22	0.28	<0.001	0.29	0.22	0.37	<0.001
**Midwest**	**Unadjusted**	0.24	0.21	0.28	<0.001	0.22	0.18	0.27	<0.001	0.21	0.14	0.31	<0.001
	**Adjusted**	0.24	0.20	0.28	<0.001	0.25	0.20	0.32	<0.001	0.26	0.17	0.39	<0.001
**South**	**Unadjusted**	0.23	0.21	0.25	<0.001	0.19	0.15	0.22	<0.001	0.18	0.12	0.27	<0.001
	**Adjusted**	0.24	0.21	0.27	<0.001	0.21	0.17	0.25	<0.001	0.22	0.15	0.33	<0.001
**West**	**Unadjusted**	0.19	0.17	0.22	<0.001	0.18	0.15	0.22	<0.001	0.16	0.11	0.25	<0.001
	**Adjusted**	0.20	0.17	0.23	<0.001	0.19	0.18	0.21	<0.001	0.18	0.11	0.29	<0.001
**New York**	**Unadjusted**	0.26	0.23	0.30	<0.001	0.26	0.22	0.30	<0.001	0.27	0.19	0.39	<0.001
	**Adjusted**	0.26	0.22	0.31	<0.001	0.25	0.21	0.30	<0.001	0.35	0.23	0.52	<0.001

LL: lower 95% confidence interval level; OR: odds ratio; UL: upper 95% confidence interval level.

**Appendix 2—table 14.** Antihypertensive Use Sensitivity Analysis, Unadjusted/Adjusted Odds Ratio for COVID-19-Related Outcomes, Stratified by Region and New York State.

		Odds of SARS-CoV-2 Test				Odds of COVID-19 Diagnosis				Odds of COVID-19 Hospitalization			
		**Antihypertensive Users versus Non-users**											
		**OR**	**LL**	**UL**	**p value**	**OR**	**LL**	**UL**	**p value**	**OR**	**LL**	**UL**	**p value**
**All**	**Unadjusted**	0.91	0.90	0.92	<0.001	0.86	0.85	0.87	<0.001	1.13	1.10	1.17	<0.001
	**Adjusted**	0.87	0.86	0.88	<0.001	0.75	0.74	0.76	<0.001	0.98	0.95	1.00	0.10
**Northeast**	**Unadjusted**	0.86	0.84	0.87	<0.001	0.83	0.82	0.85	<0.001	1.20	1.15	1.25	<0.001
	**Adjusted**	0.82	0.81	0.83	<0.001	0.72	0.71	0.73	<0.001	1.04	0.99	1.08	0.10
**Midwest**	**Unadjusted**	1.00	0.98	1.02	0.98	1.06	1.03	1.10	<0.001	1.28	1.20	1.36	<0.001
	**Adjusted**	0.94	0.91	0.96	<0.001	0.94	0.90	0.97	<0.001	1.11	1.04	1.19	0.002
**South**	**Unadjusted**	0.93	0.92	0.94	<0.001	0.88	0.86	0.90	<0.001	1.02	0.96	1.07	0.58
	**Adjusted**	0.88	0.87	0.89	<0.001	0.78	0.76	0.80	<0.001	0.89	0.84	0.94	<0.001
**West**	**Unadjusted**	0.90	0.88	0.92	<0.001	0.75	0.73	0.78	<0.001	0.99	0.92	1.06	0.83
	**Adjusted**	0.87	0.85	0.89	<0.001	0.65	0.62	0.67	<0.001	0.84	0.78	0.90	<0.001
**New York**	**Unadjusted**	0.92	0.90	0.94	<0.001	0.90	0.87	0.92	<0.001	1.23	1.15	1.31	<0.001
	**Adjusted**	0.90	0.87	0.92	<0.001	0.75	0.73	0.77	<0.001	1.01	0.95	1.09	0.70
		**BP Users versus BP Non-users among Antihypertensive Users**											
		**OR**	**LL**	**UL**	**p value**	**OR**	**LL**	**UL**	**p value**	**OR**	**LL**	**UL**	**p value**
**All**	**Unadjusted**	0.23	0.22	0.24	<0.001	0.26	0.25	0.28	<0.001	0.26	0.23	0.29	<0.001
	**Adjusted**	0.23	0.22	0.24	<0.001	0.26	0.25	0.28	<0.001	0.27	0.24	0.30	<0.001
**Northeast**	**Unadjusted**	0.24	0.22	0.26	<0.001	0.28	0.26	0.31	<0.001	0.27	0.22	0.32	<0.001
	**Adjusted**	0.23	0.21	0.26	<0.001	0.28	0.26	0.31	<0.001	0.29	0.24	0.34	<0.001
**Midwest**	**Unadjusted**	0.26	0.23	0.29	<0.001	0.27	0.23	0.31	<0.001	0.27	0.21	0.35	<0.001
	**Adjusted**	0.27	0.24	0.30	<0.001	0.28	0.26	0.30	<0.001	0.27	0.20	0.35	<0.001
**South**	**Unadjusted**	0.23	0.21	0.25	<0.001	0.24	0.22	0.28	<0.001	0.26	0.20	0.32	<0.001
	**Adjusted**	0.23	0.21	0.25	<0.001	0.24	0.21	0.28	<0.001	0.25	0.20	0.32	<0.001
**West**	**Unadjusted**	0.20	0.18	0.22	<0.001	0.21	0.18	0.25	<0.001	0.24	0.18	0.31	<0.001
	**Adjusted**	0.20	0.18	0.22	<0.001	0.22	0.18	0.25	<0.001	0.24	0.18	0.33	<0.001
**New York**	**Unadjusted**	0.26	0.23	0.29	<0.001	0.30	0.26	0.33	<0.001	0.29	0.22	0.38	<0.001
	**Adjusted**	0.25	0.22	0.29	<0.001	0.30	0.26	0.34	<0.001	0.33	0.24	0.44	<0.001
		**BP Users versus BP Non-users among Antihypertensive Non-users**											
		**OR**	**LL**	**UL**	**p value**	**OR**	**LL**	**UL**	**p value**	**OR**	**LL**	**UL**	**p value**
**All**	**Unadjusted**	0.21	0.20	0.22	<0.001	0.20	0.18	0.22	<0.001	0.21	0.17	0.25	<0.001
	**Adjusted**	0.21	0.20	0.22	<0.001	0.22	0.20	0.24	<0.001	0.27	0.22	0.32	<0.001
**Northeast**	**Unadjusted**	0.21	0.19	0.23	<0.001	0.22	0.19	0.24	<0.001	0.23	0.18	0.31	<0.001
	**Adjusted**	0.22	0.20	0.25	<0.001	0.25	0.22	0.28	<0.001	0.30	0.22	0.40	<0.001
**Midwest**	**Unadjusted**	0.22	0.19	0.25	<0.001	0.16	0.12	0.20	<0.001	0.20	0.13	0.31	<0.001
	**Adjusted**	0.21	0.18	0.25	<0.001	0.18	0.14	0.23	<0.001	0.26	0.16	0.42	<0.001
**South**	**Unadjusted**	0.20	0.18	0.22	<0.001	0.19	0.16	0.22	<0.001	0.22	0.15	0.32	<0.001
	**Adjusted**	0.20	0.18	0.22	<0.001	0.21	0.17	0.25	<0.001	0.28	0.19	0.41	<0.001
**West**	**Unadjusted**	0.19	0.17	0.22	<0.001	0.18	0.15	0.22	<0.001	0.15	0.09	0.24	<0.001
	**Adjusted**	0.20	0.17	0.22	<0.001	0.20	0.16	0.25	<0.001	0.19	0.11	0.32	<0.001
**New York**	**Unadjusted**	0.26	0.23	0.31	<0.001	0.25	0.21	0.29	<0.001	0.23	0.15	0.36	<0.001
	**Adjusted**	0.27	0.23	0.32	<0.001	0.26	0.22	0.31	<0.001	0.26	0.16	0.43	<0.001

LL: lower 95% confidence interval level; OR: odds ratio; UL: upper 95% confidence interval level.

**Appendix 2—table 15.** Antidiabetic Use Sensitivity Analysis, Unadjusted/Adjusted Odds Ratio for COVID-19-Related Outcomes, Stratified by Region and New York State.

		Odds of SARS-CoV-2 Test				Odds of COVID-19 Diagnosis				Odds of COVID-19 Hospitalization			
		**Antidiabetic Users versus Non-users**											
		**OR**	**LL**	**UL**	**p value**	**OR**	**LL**	**UL**	**p value**	**OR**	**LL**	**UL**	**p value**
**All**	**Unadjusted**	0.98	0.97	0.99	0.01	1.15	1.13	1.18	<0.001	1.50	1.45	1.56	<0.001
	**Adjusted**	0.92	0.90	0.93	<0.001	0.88	0.86	0.90	<0.001	1.13	1.08	1.18	<0.001
**Northeast**	**Unadjusted**	1.00	0.98	1.02	0.92	1.11	1.09	1.14	<0.001	1.55	1.47	1.64	<0.001
	**Adjusted**	0.94	0.92	0.97	<0.001	0.84	0.81	0.86	<0.001	1.18	1.11	1.27	<0.001
**Midwest**	**Unadjusted**	1.04	1.01	1.08	0.01	1.39	1.33	1.46	<0.001	1.61	1.47	1.76	<0.001
	**Adjusted**	0.95	0.91	0.99	0.01	1.11	1.04	1.17	<0.001	1.30	1.17	1.44	<0.001
**South**	**Unadjusted**	0.97	0.95	0.99	0.01	1.16	1.12	1.21	<0.001	1.39	1.29	1.50	<0.001
	**Adjusted**	0.90	0.88	0.93	<0.001	0.91	0.87	0.95	<0.001	1.04	0.95	1.14	0.40
**West**	**Unadjusted**	0.91	0.88	0.94	<0.001	1.07	1.01	1.12	0.01	1.43	1.30	1.58	<0.001
	**Adjusted**	0.86	0.82	0.89	<0.001	0.80	0.75	0.85	<0.001	0.97	0.86	1.09	0.60
**New York**	**Unadjusted**	1.06	1.03	1.10	<0.001	1.15	1.11	1.19	<0.001	1.59	1.46	1.72	<0.001
	**Adjusted**	1.06	1.02	1.10	0.007	0.87	0.83	0.90	<0.001	1.18	1.07	1.30	0.001
		**BP Users versus BP Non-users among Antidiabetic Users**											
		**OR**	**LL**	**UL**	**p value**	**OR**	**LL**	**UL**	**p value**	**OR**	**LL**	**UL**	**p value**
**All**	**Unadjusted**	0.26	0.24	0.28	<0.001	0.29	0.27	0.32	<0.001	0.28	0.24	0.33	<0.001
	**Adjusted**	0.26	0.24	0.28	<0.001	0.29	0.27	0.32	<0.001	0.29	0.25	0.34	<0.001
**Northeast**	**Unadjusted**	0.28	0.24	0.32	<0.001	0.32	0.28	0.35	<0.001	0.29	0.23	0.36	<0.001
	**Adjusted**	0.28	0.24	0.32	<0.001	0.31	0.27	0.35	<0.001	0.30	0.24	0.39	<0.001
**Midwest**	**Unadjusted**	0.27	0.22	0.33	<0.001	0.30	0.24	0.38	<0.001	0.28	0.19	0.41	<0.001
	**Adjusted**	0.27	0.22	0.34	<0.001	0.32	0.26	0.41	<0.001	0.29	0.19	0.42	<0.001
**South**	**Unadjusted**	0.29	0.26	0.33	<0.001	0.31	0.26	0.36	<0.001	0.35	0.26	0.47	<0.001
	**Adjusted**	0.30	0.26	0.34	<0.001	0.30	0.25	0.36	<0.001	0.36	0.26	0.48	<0.001
**West**	**Unadjusted**	0.19	0.16	0.22	<0.001	0.20	0.17	0.25	<0.001	0.21	0.15	0.30	<0.001
	**Adjusted**	0.19	0.16	0.23	<0.001	0.21	0.17	0.26	<0.001	0.22	0.15	0.31	<0.001
**New York**	**Unadjusted**	0.33	0.27	0.40	<0.001	0.34	0.29	0.39	<0.001	0.35	0.26	0.49	<0.001
	**Adjusted**	0.32	0.26	0.40	<0.001	0.32	0.28	0.36	<0.001	0.40	0.28	0.56	<0.001
		**BP Users versus BP Non-users among Antidiabetic Non-users**											
		**OR**	**LL**	**UL**	**p value**	**OR**	**LL**	**UL**	**p value**	**OR**	**LL**	**UL**	**p value**
**All**	**Unadjusted**	0.24	0.23	0.26	<0.001	0.24	0.22	0.26	<0.001	0.24	0.20	0.29	<0.001
	**Adjusted**	0.25	0.23	0.27	<0.001	0.25	0.23	0.28	<0.001	0.27	0.22	0.33	<0.001
**Northeast**	**Unadjusted**	0.24	0.22	0.28	<0.001	0.26	0.22	0.29	<0.001	0.25	0.19	0.34	<0.001
	**Adjusted**	0.25	0.22	0.29	<0.001	0.27	0.24	0.32	<0.001	0.28	0.20	0.39	<0.001
**Midwest**	**Unadjusted**	0.27	0.22	0.32	<0.001	0.22	0.17	0.30	<0.001	0.26	0.16	0.42	<0.001
	**Adjusted**	0.28	0.24	0.31	<0.001	0.23	0.17	0.31	<0.001	0.26	0.16	0.45	<0.001
**South**	**Unadjusted**	0.24	0.21	0.27	<0.001	0.25	0.20	0.30	<0.001	0.29	0.20	0.43	<0.001
	**Adjusted**	0.24	0.21	0.27	<0.001	0.24	0.21	0.28	<0.001	0.33	0.22	0.49	<0.001
**West**	**Unadjusted**	0.23	0.20	0.27	<0.001	0.18	0.14	0.24	<0.001	0.13	0.07	0.23	<0.001
	**Adjusted**	0.23	0.20	0.28	<0.001	0.20	0.15	0.26	<0.001	0.15	0.08	0.28	<0.001
**New York**	**Unadjusted**	0.30	0.25	0.37	<0.001	0.30	0.25	0.36	<0.001	0.22	0.14	0.36	<0.001
	**Adjusted**	0.30	0.25	0.37	<0.001	0.31	0.25	0.37	<0.001	0.24	0.14	0.41	<0.001

LL: lower 95% confidence interval level; OR: odds ratio; UL: upper 95% confidence interval level.

**Appendix 2—table 16.** Antidepressant Use Sensitivity Analysis, Unadjusted/Adjusted Odds Ratio for COVID-19-Related Outcomes, Stratified by Region and New York State.

		Odds of SARS-CoV-2 Test				Odds of COVID-19 Diagnosis				Odds of COVID-19 Hospitalization			
		**Antidepressant Users versus Non-users**											
		**OR**	**LL**	**UL**	**p value**	**OR**	**LL**	**UL**	**p value**	**OR**	**LL**	**UL**	**p value**
**All**	**Unadjusted**	1.04	1.03	1.05	<0.001	0.71	0.70	0.72	<0.001	0.81	0.78	0.83	<0.001
	**Adjusted**	1.00	0.99	1.01	0.61	0.65	0.64	0.66	<0.001	0.75	0.73	0.78	<0.001
**Northeast**	**Unadjusted**	1.01	0.99	1.02	0.54	0.71	0.69	0.72	<0.001	0.84	0.80	0.88	<0.001
	**Adjusted**	0.97	0.95	0.99	0.001	0.65	0.63	0.66	<0.001	0.77	0.73	0.82	<0.001
**Midwest**	**Unadjusted**	1.10	1.08	1.12	<0.001	0.75	0.72	0.78	<0.001	0.84	0.78	0.90	<0.001
	**Adjusted**	1.05	1.03	1.07	<0.001	0.69	0.66	0.71	<0.001	0.78	0.73	0.84	<0.001
**South**	**Unadjusted**	1.04	1.02	1.05	<0.001	0.68	0.66	0.70	<0.001	0.74	0.70	0.79	<0.001
	**Adjusted**	0.99	0.98	1.01	0.49	0.64	0.62	0.66	<0.001	0.72	0.68	0.77	<0.001
**West**	**Unadjusted**	1.04	1.02	1.06	0.00	0.70	0.67	0.73	<0.001	0.77	0.70	0.84	<0.001
	**Adjusted**	0.99	0.97	1.02	0.46	0.64	0.61	0.66	<0.001	0.70	0.64	0.77	<0.001
**New York**	**Unadjusted**	1.00	0.97	1.03	0.86	0.77	0.74	0.80	<0.001	0.83	0.76	0.91	<0.001
	**Adjusted**	0.98	0.95	1.01	0.27	0.72	0.70	0.75	<0.001	0.77	0.70	0.85	<0.001
		**BP Users versus BP Non-users among Antidepressant Users**											
		**OR**	**LL**	**UL**	**p value**	**OR**	**LL**	**UL**	**p value**	**OR**	**LL**	**UL**	**p value**
**All**	**Unadjusted**	0.27	0.26	0.28	<0.001	0.30	0.28	0.32	<0.001	0.31	0.27	0.36	<0.001
	**Adjusted**	0.27	0.25	0.28	<0.001	0.30	0.28	0.32	<0.001	0.33	0.28	0.38	<0.001
**Northeast**	**Unadjusted**	0.28	0.26	0.31	<0.001	0.33	0.30	0.37	<0.001	0.36	0.29	0.45	<0.001
	**Adjusted**	0.28	0.25	0.30	<0.001	0.32	0.29	0.36	<0.001	0.37	0.29	0.47	<0.001
**Midwest**	**Unadjusted**	0.30	0.27	0.34	<0.001	0.26	0.22	0.31	<0.001	0.25	0.18	0.34	<0.001
	**Adjusted**	0.30	0.26	0.34	<0.001	0.27	0.22	0.33	<0.001	0.26	0.18	0.36	<0.001
**South**	**Unadjusted**	0.26	0.24	0.29	<0.001	0.27	0.23	0.31	<0.001	0.32	0.24	0.41	<0.001
	**Adjusted**	0.26	0.24	0.28	<0.001	0.27	0.23	0.32	<0.001	0.32	0.24	0.43	<0.001
**West**	**Unadjusted**	0.25	0.22	0.28	<0.001	0.27	0.22	0.32	<0.001	0.29	0.20	0.41	<0.001
	**Adjusted**	0.24	0.21	0.27	<0.001	0.29	0.28	0.30	<0.001	0.33	0.23	0.48	<0.001
**New York**	**Unadjusted**	0.30	0.26	0.34	<0.001	0.33	0.28	0.38	<0.001	0.24	0.16	0.36	<0.001
	**Adjusted**	0.30	0.25	0.34	<0.001	0.31	0.27	0.37	<0.001	0.25	0.16	0.39	<0.001
		**BP Users versus BP Non-users among Antidepressant Non-users**											
		**OR**	**LL**	**UL**	**p value**	**OR**	**LL**	**UL**	**p value**	**OR**	**LL**	**UL**	**p value**
**All**	**Unadjusted**	0.20	0.19	0.22	<0.001	0.22	0.20	0.24	<0.001	0.24	0.20	0.28	<0.001
	**Adjusted**	0.21	0.19	0.22	<0.001	0.23	0.21	0.25	<0.001	0.27	0.22	0.32	<0.001
**Northeast**	**Unadjusted**	0.21	0.19	0.24	<0.001	0.23	0.20	0.26	<0.001	0.25	0.19	0.32	<0.001
	**Adjusted**	0.22	0.19	0.25	<0.001	0.24	0.22	0.25	<0.001	0.29	0.22	0.39	<0.001
**Midwest**	**Unadjusted**	0.22	0.19	0.26	<0.001	0.23	0.18	0.28	<0.001	0.28	0.19	0.39	<0.001
	**Adjusted**	0.21	0.18	0.25	<0.001	0.26	0.24	0.27	<0.001	0.32	0.22	0.47	<0.001
**South**	**Unadjusted**	0.20	0.18	0.22	<0.001	0.21	0.18	0.25	<0.001	0.21	0.15	0.30	<0.001
	**Adjusted**	0.20	0.18	0.23	<0.001	0.23	0.19	0.27	<0.001	0.22	0.16	0.32	<0.001
**West**	**Unadjusted**	0.18	0.16	0.21	<0.001	0.20	0.16	0.25	<0.001	0.20	0.13	0.30	<0.001
	**Adjusted**	0.19	0.16	0.22	<0.001	0.20	0.20	0.21	<0.001	0.22	0.14	0.35	<0.001
**New York**	**Unadjusted**	0.26	0.22	0.32	<0.001	0.27	0.23	0.32	<0.001	0.29	0.19	0.43	<0.001
	**Adjusted**	0.26	0.23	0.30	<0.001	0.26	0.22	0.32	<0.001	0.35	0.22	0.54	<0.001

LL: lower 95% confidence interval level; OR: odds ratio; UL: upper 95% confidence interval level.

**Appendix 2—table 17.** “*Bone-Rx*” Cohort (All Regions), Patient Characteristics Pre/Post Match.

	"Bone-Rx" Cohort / All Observations Unmatched							"Bone-Rx" Cohort / All Observations Matched						
	**All**		**BP Non-user**		**BP User**		**p-value**	**All**		**BP Non-user**		**BP User**		**p-value**
	**N**	**%**	**N**	**%**	**N**	**%**		**N**	**%**	**N**	**%**	**N**	**%**	
**All Patients**	**502,895**	**100.0%**	**50,844**	**10.1%**	**452,051**	**89.9%**		**100,996**	**100.0%**	**50,498**	**50.0%**	**50,498**	**50.0%**	
**Age**														
≤20	1,164	0.2%	36	0.1%	1,128	0.2%	<0.001	67	0.1%	36	0.1%	31	0.1%	0.97
21-40	3,501	0.7%	410	0.8%	3,091	0.7%		790	0.8%	403	0.8%	387	0.8%	
41-50	9,631	1.9%	1,080	2.1%	8,551	1.9%		2,107	2.1%	1,069	2.1%	1,038	2.1%	
51-60	72,139	14.3%	6,418	12.6%	65,721	14.5%		12,777	12.7%	6,395	12.7%	6,382	12.6%	
61-70	171,687	34.1%	14,809	29.1%	156,878	34.7%		29,509	29.2%	14,751	29.2%	14,758	29.2%	
71-80	157,877	31.4%	16,152	31.8%	141,725	31.4%		32,129	31.8%	16,055	31.8%	16,074	31.8%	
≥81	86,896	17.3%	11,939	23.5%	74,957	16.6%		23,617	23.4%	11,789	23.3%	11,828	23.4%	
**Gender**														
Female	451,790	89.8%	44,354	87.2%	407,436	90.1%	<0.001	88,552	87.7%	44,235	87.6%	44,317	87.8%	0.43
Male	51,105	10.2%	6,490	12.8%	44,615	9.9%		12,444	12.3%	6,263	12.4%	6,181	12.2%	
**Region**														
Midwest	85,391	17.0%	9,424	18.5%	75,967	16.8%	<0.001	18,720	18.5%	9,360	18.5%	9,360	18.5%	1.00
Northeast	135,867	27.0%	16,139	31.7%	119,728	26.5%		31,986	31.7%	15,993	31.7%	15,993	31.7%	
South	178,118	35.4%	17,232	33.9%	160,886	35.6%		34,280	33.9%	17,140	33.9%	17,140	33.9%	
West	103,519	20.6%	8,049	15.8%	95,470	21.1%		16,010	15.9%	8,005	15.9%	8,005	15.9%	
**Insurance**														
Commercial	164,150	32.6%	17,092	33.6%	147,058	32.5%	<0.001	33,977	33.6%	16,963	33.6%	17,014	33.7%	0.91
Dual	33,969	6.8%	2,562	5.0%	31,407	6.9%		5,056	5.0%	2,547	5.0%	2,509	5.0%	
Medicaid	84,514	16.8%	7,034	13.8%	77,480	17.1%		13,925	13.8%	6,986	13.8%	6,939	13.7%	
Medicare	220,262	43.8%	24,156	47.5%	196,106	43.4%		48,038	47.6%	24,002	47.5%	24,036	47.6%	
**PCP Visit 2019**														
No	181,996	36.2%	18,130	35.7%	163,866	36.2%	0.009	35,943	35.6%	17,979	35.6%	17,964	35.6%	0.92
Yes	320,899	63.8%	32,714	64.3%	288,185	63.8%		65,053	64.4%	32,519	64.4%	32,534	64.4%	
**Continuous Outcomes**														
	**mean**	**SD**	**mean**	**SD**	**mean**	**SD**	**p-value**	**mean**	**SD**	**mean**	**SD**	**mean**	**SD**	**p-value**
**CCI**	1.05	1.91	1.99	2.71	0.95	1.76	<0.001	1.93	2.59	1.93	2.60	1.92	2.59	0.76

BP: bisphosphonate; CCI: Charlson Comorbidity Index; PCP: primary care physician; SD: standard deviation.

**Appendix 2—table 18.** “*Bone-Rx*” Cohort (Region=Northeast), Patient Characteristics Pre/Post Match.

	"Bone-Rx" Cohort / Region=Northeast Unmatched							"Bone-Rx" Cohort / Region=Northeast Matched						
	**All**		**BP Non-user**		**BP User**		**p-value**	**All**		**BP Non-user**		**BP User**		**p-value**
	**N**	**%**	**N**	**%**	**N**	**%**		**N**	**%**	**N**	**%**	**N**	**%**	
**All Patients**	**135,867**	**100.0%**	**16,139**	**11.9%**	**119,728**	**88.1%**		**31,986**	**100.0%**	**15,993**	**50.0%**	**15,993**	**50.0%**	
**Age**														
≤20	245	0.2%	≤10	0.1%	236	0.2%	<0.001	15	0.0%	≤10	0.1%	≤10	0.0%	0.99
21-40	891	0.7%	127	0.8%	764	0.6%		250	0.8%	124	0.8%	126	0.8%	
41-50	2,340	1.7%	298	1.8%	2,042	1.7%		570	1.8%	290	1.8%	280	1.8%	
51-60	20,069	14.8%	2,059	12.8%	18,010	15.0%		4,088	12.8%	2,049	12.8%	2,039	12.7%	
61-70	45,896	33.8%	4,802	29.8%	41,094	34.3%		9,526	29.8%	4,767	29.8%	4,759	29.8%	
71-80	42,828	31.5%	5,267	32.6%	37,561	31.4%		10,465	32.7%	5,226	32.7%	5,239	32.8%	
≥81	23,598	17.4%	3,577	22.2%	20,021	16.7%		7,072	22.1%	3,528	22.1%	3,544	22.2%	
**Gender**														
Female	122,485	90.2%	14,115	87.5%	108,370	90.5%	<0.001	28,157	88.0%	14,062	87.9%	14,095	88.1%	0.57
Male	13,382	9.8%	2,024	12.5%	11,358	9.5%		3,829	12.0%	1,931	12.1%	1,898	11.9%	
**Insurance**														
Commercial	37,810	27.8%	4,517	28.0%	33,293	27.8%	<0.001	8,927	27.9%	4,459	27.9%	4,468	27.9%	0.99
Dual	8,434	6.2%	829	5.1%	7,605	6.4%		1,637	5.1%	824	5.2%	813	5.1%	
Medicaid	25,296	18.6%	2,082	12.9%	23,214	19.4%		4,122	12.9%	2,067	12.9%	2,055	12.8%	
Medicare	64,327	47.3%	8,711	54.0%	55,616	46.5%		17,300	54.1%	8,643	54.0%	8,657	54.1%	
**PCP Visit 2019**														
No	56,593	41.7%	6,726	41.7%	49,867	41.7%	0.95	13,307	41.6%	6,654	41.6%	6,653	41.6%	0.99
Yes	79,274	58.3%	9,413	58.3%	69,861	58.3%		18,679	58.4%	9,339	58.4%	9,340	58.4%	
**Continuous Outcomes**														
	**mean**	**SD**	**mean**	**SD**	**mean**	**SD**	**p-value**	**mean**	**SD**	**mean**	**SD**	**mean**	**SD**	**p-value**
**CCI**	1.06	1.89	1.97	2.70	0.93	1.71	<0.001	1.89	2.57	1.89	2.58	1.89	2.57	0.91

BP: bisphosphonate; CCI: Charlson Comorbidity Index; PCP: primary care physician; SD: standard deviation.

**Appendix 2—table 19.** “Bone-Rx” Cohort (Region=Midwest), Patient Characteristics Pre/Post Match.

	"Bone-Rx" Cohort / Region=Midwest Unmatched							"Bone-Rx" Cohort / Region=Midwest Matched						
	**All**		**BP Non-user**		**BP User**		**p-value**	**All**		**BP Non-user**		**BP User**		**p-value**
	**N**	**%**	**N**	**%**	**N**	**%**		**N**	**%**	**N**	**%**	**N**	**%**	
**All Patients**	**85,391**	**100.0%**	**9,424**	**11.0%**	**75,967**	**89.0%**		**18,720**	**100.0%**	**9,360**	**50.0%**	**9,360**	**50.0%**	
**Age**														
≤20	274	0.3%	≤10	0.1%	268	0.4%	<0.001	13	0.1%	≤10	0.1%	≤10	0.1%	1.00
21-40	672	0.8%	79	0.8%	593	0.8%		154	0.8%	78	0.8%	76	0.8%	
41-50	1,886	2.2%	202	2.1%	1,684	2.2%		389	2.1%	200	2.1%	189	2.0%	
51-60	13,522	15.8%	1,284	13.6%	12,238	16.1%		2,559	13.7%	1,280	13.7%	1,279	13.7%	
61-70	31,256	36.6%	2,760	29.3%	28,496	37.5%		5,512	29.4%	2,754	29.4%	2,758	29.5%	
71-80	23,887	28.0%	2,766	29.4%	21,121	27.8%		5,492	29.3%	2,748	29.4%	2,744	29.3%	
≥81	13,894	16.3%	2,327	24.7%	11,567	15.2%		4,601	24.6%	2,294	24.5%	2,307	24.6%	
**Gender**														
Female	76,696	89.8%	8,118	86.1%	68,578	90.3%	<0.001	16,223	86.7%	8,102	86.6%	8,121	86.8%	0.68
Male	8,695	10.2%	1,306	13.9%	7,389	9.7%		2,497	13.3%	1,258	13.4%	1,239	13.2%	
**Insurance**														
Commercial	34,494	40.4%	3,361	35.7%	31,133	41.0%	<0.001	6,699	35.8%	3,345	35.7%	3,354	35.8%	0.96
Dual	4,042	4.7%	436	4.6%	3,606	4.7%		852	4.6%	429	4.6%	423	4.5%	
Medicaid	8,856	10.4%	733	7.8%	8,123	10.7%		1,441	7.7%	729	7.8%	712	7.6%	
Medicare	37,999	44.5%	4,894	51.9%	33,105	43.6%		9,728	52.0%	4,857	51.9%	4,871	52.0%	
**PCP Visit 2019**														
No	32,037	37.5%	3,330	35.3%	28,707	37.8%	<0.001	6,628	35.4%	3,312	35.4%	3,316	35.4%	0.95
Yes	53,354	62.5%	6,094	64.7%	47,260	62.2%		12,092	64.6%	6,048	64.6%	6,044	64.6%	
**Continuous Outcomes**														
	**mean**	**SD**	**mean**	**SD**	**mean**	**SD**	**p-value**	**mean**	**SD**	**mean**	**SD**	**mean**	**SD**	**p-value**
**CCI**	1.12	2.02	2.12	2.83	0.99	1.86	<0.001	2.05	2.72	2.06	2.72	2.05	2.72	0.91

BP: bisphosphonate; CCI: Charlson Comorbidity Index; PCP: primary care physician; SD: standard deviation.

**Appendix 2—table 20.** “*Bone-Rx*” Cohort (Region=South), Patient Characteristics Pre/Post Match.

	"Bone-Rx" Cohort / Region=South Unmatched							"Bone-Rx" Cohort / Region=South Matched						
	**All**		**BP Non-user**		**BP User**		**p-value**	**All**		**BP Non-user**		**BP User**		**p-value**
	**N**	**%**	**N**	**%**	**N**	**%**		**N**	**%**	**N**	**%**	**N**	**%**	
**All Patients**	**178,118**	**100.0%**	**17,232**	**9.7%**	**160,886**	**90.3%**		**34,280**	**100.0%**	**17,140**	**50.0%**	**17,140**	**50.0%**	
**Age**														
≤20	490	0.3%	16	0.1%	474	0.3%	<0.001	31	0.1%	16	0.1%	15	0.1%	1.00
21-40	1,313	0.7%	136	0.8%	1,177	0.7%		262	0.8%	134	0.8%	128	0.7%	
41-50	3,866	2.2%	445	2.6%	3,421	2.1%		884	2.6%	444	2.6%	440	2.6%	
51-60	27,389	15.4%	2,296	13.3%	25,093	15.6%		4,574	13.3%	2,290	13.4%	2,284	13.3%	
61-70	61,038	34.3%	5,142	29.8%	55,896	34.7%		10,271	30.0%	5,129	29.9%	5,142	30.0%	
71-80	56,126	31.5%	5,521	32.0%	50,605	31.5%		10,990	32.1%	5,493	32.0%	5,497	32.1%	
≥81	27,896	15.7%	3,676	21.3%	24,220	15.1%		7,268	21.2%	3,634	21.2%	3,634	21.2%	
**Gender**														
Female	160,994	90.4%	15,179	88.1%	145,815	90.6%	<0.001	30,322	88.5%	15,149	88.4%	15,173	88.5%	0.69
Male	17,124	9.6%	2,053	11.9%	15,071	9.4%		3,958	11.5%	1,991	11.6%	1,967	11.5%	
**Insurance**														
Commercial	66,332	37.2%	7,042	40.9%	59,290	36.9%	<0.001	14,052	41.0%	7,007	40.9%	7,045	41.1%	0.95
Dual	14,829	8.3%	769	4.5%	14,060	8.7%		1,523	4.4%	769	4.5%	754	4.4%	
Medicaid	23,492	13.2%	1,843	10.7%	21,649	13.5%		3,639	10.6%	1,829	10.7%	1,810	10.6%	
Medicare	73,465	41.2%	7,578	44.0%	65,887	41.0%		15,066	43.9%	7,535	44.0%	7,531	43.9%	
**PCP Visit 2019**														
No	60,253	33.8%	5,785	33.6%	54,468	33.9%	0.454	11,462	33.4%	5,736	33.5%	5,726	33.4%	0.91
Yes	117,865	66.2%	11,447	66.4%	106,418	66.1%		22,818	66.6%	11,404	66.5%	11,414	66.6%	
**Continuous Outcomes**														
	**mean**	**SD**	**mean**	**SD**	**mean**	**SD**	**p-value**	**mean**	**SD**	**mean**	**SD**	**mean**	**SD**	**p-value**
**CCI**	0.95	1.84	1.86	2.65	0.86	1.70	<0.001	1.80	2.54	1.80	2.54	1.79	2.53	0.78

BP: bisphosphonate; CCI: Charlson Comorbidity Index; PCP: primary care physician; SD: standard deviation.

**Appendix 2—table 21.** “*Bone-Rx*” Cohort (Region=West), Patient Characteristics Pre/Post Match.

	"Bone-Rx" Cohort / Region=West Unmatched							"Bone-Rx" Cohort / Region=West Matched						
	**All**		**BP Non-user**		**BP User**		**p-value**	**All**		**BP Non-user**		**BP User**		**p-value**
	**N**	**%**	**N**	**%**	**N**	**%**		**N**	**%**	**N**	**%**	**N**	**%**	
**All Patients**	**103,519**	**100.0%**	**8,049**	**7.8%**	**95,470**	**92.2%**		**16,010**	**100.0%**	**8,005**	**50.0%**	**8,005**	**50.0%**	
**Age**														
≤20	155	0.1%	≤10	0.1%	150	0.2%	<0.001	≤10	0.0%	≤10	0.1%	≤10	0.0%	0.96
21-40	625	0.6%	68	0.8%	557	0.6%		124	0.8%	67	0.8%	57	0.7%	
41-50	1,539	1.5%	135	1.7%	1,404	1.5%		264	1.6%	135	1.7%	129	1.6%	
51-60	11,159	10.8%	779	9.7%	10,380	10.9%		1,556	9.7%	776	9.7%	780	9.7%	
61-70	33,497	32.4%	2,105	26.2%	31,392	32.9%		4,200	26.2%	2,101	26.2%	2,099	26.2%	
71-80	35,036	33.8%	2,598	32.3%	32,438	34.0%		5,182	32.4%	2,588	32.3%	2,594	32.4%	
≥81	21,508	20.8%	2,359	29.3%	19,149	20.1%		4,676	29.2%	2,333	29.1%	2,343	29.3%	
**Gender**														
Female	91,615	88.5%	6,942	86.2%	84,673	88.7%	<0.001	13,850	86.5%	6,922	86.5%	6,928	86.5%	0.89
Male	11,904	11.5%	1,107	13.8%	10,797	11.3%		2,160	13.5%	1,083	13.5%	1,077	13.5%	
**Insurance**														
Commercial	25,514	24.6%	2,172	27.0%	23,342	24.4%	<0.001	4,299	26.9%	2,152	26.9%	2,147	26.8%	1.00
Dual	6,664	6.4%	528	6.6%	6,136	6.4%		1,044	6.5%	525	6.6%	519	6.5%	
Medicaid	26,870	26.0%	2,376	29.5%	24,494	25.7%		4,723	29.5%	2,361	29.5%	2,362	29.5%	
Medicare	44,471	43.0%	2,973	36.9%	41,498	43.5%		5,944	37.1%	2,967	37.1%	2,977	37.2%	
**PCP Visit 2019**														
No	33,113	32.0%	2,289	28.4%	30,824	32.3%	<0.001	4,546	28.4%	2,277	28.4%	2,269	28.3%	0.89
Yes	70,406	68.0%	5,760	71.6%	64,646	67.7%		11,464	71.6%	5,728	71.6%	5,736	71.7%	
**Continuous Outcomes**														
	**mean**	**SD**	**mean**	**SD**	**mean**	**SD**	**p-value**	**mean**	**SD**	**mean**	**SD**	**mean**	**SD**	**p-value**
**CCI**	1.17	1.94	2.17	2.67	1.08	1.84	<0.001	2.12	2.59	2.12	2.59	2.12	2.59	0.93

BP: bisphosphonate; CCI: Charlson Comorbidity Index; PCP: primary care physician; SD: standard deviation.

**Appendix 2—table 22.** “*Bone-Rx*” Cohort (Region=New York State), Patient Characteristics Pre/Post Match.

"Bone-Rx" Cohort / Region=New York State Unmatched							"Bone-Rx" Cohort / Region=New York State Matched						
	**All**		**BP Non-user**		**BP User**		**p-value**	**All**		**BP Non-user**		**BP User**		**p-value**
	**N**	**%**	**N**	**%**	**N**	**%**		**N**	**%**	**N**	**%**	**N**	**%**	
**All Patients**	**57,397**	**100.0%**	**7,362**	**12.8%**	**50,035**	**87.2%**		**14,508**	**100.0%**	**7,254**	**50.0%**	**7,254**	**50.0%**	
**Age**														
≤20	56	0.1%	≤10	0.1%	50	0.1%	<0.001	11	0.1%	≤10	0.1%	≤10	0.1%	0.96
21-40	272	0.5%	44	0.6%	228	0.5%		76	0.5%	42	0.6%	34	0.5%	
41-50	775	1.4%	120	1.6%	655	1.3%		207	1.4%	107	1.5%	100	1.4%	
51-60	7,249	12.6%	885	12.0%	6,364	12.7%		1,744	12.0%	871	12.0%	873	12.0%	
61-70	18,433	32.1%	2,297	31.2%	16,136	32.2%		4,540	31.3%	2,264	31.2%	2,276	31.4%	
71-80	19,944	34.7%	2,482	33.7%	17,462	34.9%		4,934	34.0%	2,455	33.8%	2,479	34.2%	
≥81	10,668	18.6%	1,528	20.8%	9,140	18.3%		2,996	20.7%	1,509	20.8%	1,487	20.5%	
**Gender**														
Female	52,047	90.7%	6,589	89.5%	45,458	90.9%	<.001	13,106	90.3%	6,526	90.0%	6,580	90.7%	0.13
Male	5,350	9.3%	773	10.5%	4,577	9.1%		1,402	9.7%	728	10.0%	674	9.3%	
**Insurance**														
Commercial	12,309	21.4%	1,894	25.7%	10,415	20.8%	<0.001	3,706	25.5%	1,850	25.5%	1,856	25.6%	1.00
Dual	1,750	3.0%	154	2.1%	1,596	3.2%		307	2.1%	153	2.1%	154	2.1%	
Medicaid	10,191	17.8%	1,016	13.8%	9,175	18.3%		1,968	13.6%	987	13.6%	981	13.5%	
Medicare	33,147	57.8%	4,298	58.4%	28,849	57.7%		8,527	58.8%	4,264	58.8%	4,263	58.8%	
**PCP Visit 2019**														
No	21,462	37.4%	2,789	37.9%	18,673	37.3%	0.35	5,468	37.7%	2,744	37.8%	2,724	37.6%	0.73
Yes	35,935	62.6%	4,573	62.1%	31,362	62.7%		9,040	62.3%	4,510	62.2%	4,530	62.4%	
**Continuous Outcomes**														
	**mean**	**SD**	**mean**	**SD**	**mean**	**SD**	**p-value**	**mean**	**SD**	**mean**	**SD**	**mean**	**SD**	**p-value**
**CCI**	1.06	1.84	1.81	2.56	0.95	1.68	<0.001	1.69	2.35	1.69	2.36	1.69	2.35	0.98

BP: bisphosphonate; CCI: Charlson Comorbidity Index; PCP: primary care physician; SD: standard deviation.

**Appendix 2—table 23.** “*Osteo-Dx-Rx*” Cohort, Patient Characteristics Pre/Post Match.

	"Osteo-Dx-Rx" Cohort / All Observations Unmatched							"Osteo-Dx-Rx" Cohort / All Observations Matched						
	**All**		**BP Non-user**		**BP User**		**p-value**	**All**		**BP Non-user**		**BP User**		**p-value**
	**N**	**%**	**N**	**%**	**N**	**%**		**N**	**%**	**N**	**%**	**N**	**%**	
**All Patients**	**60,043**	**100.0%**	**8,392**	**14.0%**	**51,651**	**86.0%**		**15,898**	**100.0%**	**7,949**	**50.0%**	**7,949**	**50.0%**	
**Age**														
51-60	6,443	10.7%	753	9.0%	5,690	11.0%	<0.001	1,430	9.0%	723	9.1%	707	8.9%	0.95
61-70	20,187	33.6%	2,492	29.7%	17,695	34.3%		4,821	30.3%	2,397	30.2%	2,424	30.5%	
71-80	21,545	35.9%	2,964	35.3%	18,581	36.0%		5,677	35.7%	2,841	35.7%	2,836	35.7%	
≥81	11,868	19.8%	2,183	26.0%	9,685	18.8%		3,970	25.0%	1,988	25.0%	1,982	24.9%	
**State**														
CA	24,489	40.8%	2,558	30.5%	21,931	42.5%	<0.001	4,886	30.7%	2,443	30.7%	2,443	30.7%	1.00
FL	11,904	19.8%	1,767	21.1%	10,137	19.6%		3,256	20.5%	1,628	20.5%	1,628	20.5%	
IL	4,447	7.4%	678	8.1%	3,769	7.3%		1,168	7.3%	584	7.3%	584	7.3%	
NY	19,203	32.0%	3,389	40.4%	15,814	30.6%		6,588	41.4%	3,294	41.4%	3,294	41.4%	
**Insurance**														
Commercial	12,990	21.6%	2,048	24.4%	10,942	21.2%	<0.001	3,736	23.5%	1,868	23.5%	1,868	23.5%	1.00
Dual	3,652	6.1%	313	3.7%	3,339	6.5%		554	3.5%	277	3.5%	277	3.5%	
Medicaid	13,698	22.8%	1,785	21.3%	11,913	23.1%		3,392	21.3%	1,696	21.3%	1,696	21.3%	
Medicare	29,703	49.5%	4,246	50.6%	25,457	49.3%		8,216	51.7%	4,108	51.7%	4,108	51.7%	
**PCP Visit 2019**														
No	14,089	23.5%	2,427	28.9%	11,662	22.6%	<0.001	4,487	28.2%	2,243	28.2%	2,244	28.2%	0.99
Yes	45,954	76.5%	5,965	71.1%	39,989	77.4%		11,411	71.8%	5,706	71.8%	5,705	71.8%	
**Cancer Dx**														
No	52,301	87.1%	6,765	80.6%	45,536	88.2%	<0.001	13,116	82.5%	6,548	82.4%	6,568	82.6%	0.68
Yes	7,742	12.9%	1,627	19.4%	6,115	11.8%		2,782	17.5%	1,401	17.6%	1,381	17.4%	
**COPD Dx**														
No	53,446	89.0%	7,035	83.8%	46,411	89.9%	<0.001	13,705	86.2%	6,834	86.0%	6,871	86.4%	0.39
Yes	6,597	11.0%	1,357	16.2%	5,240	10.1%		2,193	13.8%	1,115	14.0%	1,078	13.6%	
**Heart Failure Dx**														
No	56,005	93.3%	7,492	89.3%	48,513	93.9%	<0.001	14,475	91.0%	7,218	90.8%	7,257	91.3%	0.28
Yes	4,038	6.7%	900	10.7%	3,138	6.1%		1,423	9.0%	731	9.2%	692	8.7%	
**Hypertension Dx**														
No	24,966	41.6%	3,281	39.1%	21,685	42.0%	<0.001	6,268	39.4%	3,137	39.5%	3,131	39.4%	0.92
Yes	35,077	58.4%	5,111	60.9%	29,966	58.0%		9,630	60.6%	4,812	60.5%	4,818	60.6%	
**Dyslipidemia Dx**														
No	24,095	40.1%	3,295	39.3%	20,800	40.3%	0.08	6,187	38.9%	3,101	39.0%	3,086	38.8%	0.81
Yes	35,948	59.9%	5,097	60.7%	30,851	59.7%		9,711	61.1%	4,848	61.0%	4,863	61.2%	
**Obesity Dx**														
No	53,453	89.0%	7,583	90.4%	45,870	88.8%	<0.001	14,468	91.0%	7,217	90.8%	7,251	91.2%	0.35
Yes	6,590	11.0%	809	9.6%	5,781	11.2%		1,430	9.0%	732	9.2%	698	8.8%	
**Type 2 Diabetes Dx**														
No	44,565	74.2%	6,132	73.1%	38,433	74.4%	0.009	11,759	74.0%	5,859	73.7%	5,900	74.2%	0.46
Yes	15,478	25.8%	2,260	26.9%	13,218	25.6%		4,139	26.0%	2,090	26.3%	2,049	25.8%	
**Depression Dx**														
No	51,609	86.0%	7,114	84.8%	44,495	86.1%	0.001	13,697	86.2%	6,844	86.1%	6,853	86.2%	0.84
Yes	8,434	14.0%	1,278	15.2%	7,156	13.9%		2,201	13.8%	1,105	13.9%	1,096	13.8%	

BP: bisphosphonate; CCI: Charlson Comorbidity Index; CA: California; Dx: diagnosis; FL: Florida; IL: Illinois; NY: New York; PCP: primary care physician.

**Appendix 2—table 24.** Statin Cohort (All Regions), Patient Characteristics Pre/Post Match.

	All Observations by Statin Use: Unmatched							All Observations by Statin Use: Matched						
	All		Statin Non-users		Statin Users		p-value	All		Statin Non-users		Statin Users		p-value
	N	%	N	%	N	%		N	%	N	%	N	%	
All Patients	7,906,603	100.00%	6,403,208	81.00%	1,503,395	19.00%		2,872,600	100.00%	1,436,300	50.00%	1,436,300	50.00%	
Age														
≤20	1,840,050	23.30%	1,838,665	28.70%	1,385	0.10%	<0.001	2,772	0.10%	1,387	0.10%	1,385	0.10%	0.11
21-40	1,446,999	18.30%	1,402,606	21.90%	44,393	3.00%		88,760	3.10%	44,371	3.10%	44,389	3.10%	
41-50	925,309	11.70%	789,385	12.30%	135,924	9.00%		271,615	9.50%	135,748	9.50%	135,867	9.50%	
51-60	1,250,190	15.80%	888,510	13.90%	361,680	24.10%		710,481	24.70%	354,449	24.70%	356,032	24.80%	
61-70	1,181,261	14.90%	728,702	11.40%	452,559	30.10%		857,269	29.80%	428,326	29.80%	428,943	29.90%	
71-80	783,775	9.90%	452,267	7.10%	331,508	22.10%		605,360	21.10%	303,279	21.10%	302,081	21.00%	
≥81	479,019	6.10%	303,073	4.70%	175,946	11.70%		336,343	11.70%	168,740	11.70%	167,603	11.70%	
Gender														
Female	4,670,960	59.10%	3,785,061	59.10%	885,899	58.90%	<0.001	1,682,354	58.60%	839,207	58.40%	843,147	58.70%	<0.001
Male	3,235,643	40.90%	2,618,147	40.90%	617,496	41.10%		1,190,246	41.40%	597,093	41.60%	593,153	41.30%	
Region														
Midwest	1,467,802	18.60%	1,188,569	18.60%	279,233	18.60%	<0.001	542,638	18.90%	271,319	18.90%	271,319	18.90%	1
Northeast	2,152,560	27.20%	1,706,021	26.60%	446,539	29.70%		847,868	29.50%	423,934	29.50%	423,934	29.50%	
South	3,042,604	38.50%	2,490,630	38.90%	551,974	36.70%		1,046,224	36.40%	523,112	36.40%	523,112	36.40%	
West	1,243,637	15.70%	1,017,988	15.90%	225,649	15.00%		435,870	15.20%	217,935	15.20%	217,935	15.20%	
Insurance														
Commercial	3,938,603	49.80%	3,350,332	52.30%	588,271	39.10%	<0.001	1,175,472	40.90%	587,847	40.90%	587,625	40.90%	0.34
Dual	156,497	2.00%	73,532	1.10%	82,965	5.50%		110,207	3.80%	54,851	3.80%	55,356	3.90%	
Medicaid	2,594,500	32.80%	2,254,531	35.20%	339,969	22.60%		641,345	22.30%	320,434	22.30%	320,911	22.30%	
Medicare	1,217,003	15.40%	724,813	11.30%	492,190	32.70%		945,576	32.90%	473,168	32.90%	472,408	32.90%	
PCP Visit 2019														
No	4,283,697	54.20%	3,773,784	58.90%	509,913	33.90%	<0.001	1,016,313	35.40%	508,587	35.40%	507,726	35.30%	0.29
Yes	3,622,906	45.80%	2,629,424	41.10%	993,482	66.10%		1,856,287	64.60%	927,713	64.60%	928,574	64.70%	
Continuous Outcomes														
	mean	SD	mean	SD	mean	SD	p-value	mean	SD	mean	SD	mean	SD	p-value
CCI	0.62	1.38	0.49	1.23	1.15	1.79	<0.001	1.11	1.77	1.12	1.79	1.11	1.75	<0.001

CCI: Charlson Comorbidity Index; PCP: primary care physician; SD: standard deviation.

**Appendix 2—table 25.** Statin Cohort (Region=New York State), Patient Characteristics Pre/Post Match.

	Region=NY by Statin Use: Unmatched							Region=NY by Statin Use: Matched						
	**All**		**Statin Non-users**		**Statin Users**		**p-value**	**All**		**Statin Non-users**		**Statin Users**		**p-value**
	**N**	**%**	**N**	**%**	**N**	**%**		**N**	**%**	**N**	**%**	**N**	**%**	
**All Patients**	**968,296**	**100.0%**	**761,995**	**78.7%**	**206,301**	**21.3%**		**371,072**	**100.0%**	**185,536**	**50.0%**	**185,536**	**50.0%**	
**Age**														
≤20	133,178	13.8%	133,111	17.5%	67	0.0%	<0.001	134	0.0%	67	0.0%	67	0.0%	1.00
21-40	192,959	19.9%	188,446	24.7%	4,513	2.2%		9,019	2.4%	4,508	2.4%	4,511	2.4%	
41-50	127,794	13.2%	112,342	14.7%	15,452	7.5%		30,860	8.3%	15,420	8.3%	15,440	8.3%	
51-60	172,444	17.8%	128,472	16.9%	43,972	21.3%		86,136	23.2%	43,068	23.2%	43,068	23.2%	
61-70	159,912	16.5%	100,884	13.2%	59,028	28.6%		106,460	28.7%	53,233	28.7%	53,227	28.7%	
71-80	120,117	12.4%	64,549	8.5%	55,568	26.9%		91,337	24.6%	45,675	24.6%	45,662	24.6%	
≥81	61,892	6.4%	34,191	4.5%	27,701	13.4%		47,126	12.7%	23,565	12.7%	23,561	12.7%	
**Gender**														
Female	573,610	59.2%	454,050	59.6%	119,560	58.0%	<0.001	215,375	58.0%	107,420	57.9%	107,955	58.2%	0.08
Male	394,686	40.8%	307,945	40.4%	86,741	42.0%		155,697	42.0%	78,116	42.1%	77,581	41.8%	
**Insurance**														
Commercial	500,918	51.7%	442,990	58.1%	57,928	28.1%	<0.001	116,123	31.3%	58,206	31.4%	57,917	31.2%	0.57
Dual	6,814	0.7%	2,410	0.3%	4,404	2.1%		4,447	1.2%	2,190	1.2%	2,257	1.2%	
Medicaid	252,366	26.1%	206,109	27.0%	46,257	22.4%		83,550	22.5%	41,703	22.5%	41,847	22.6%	
Medicare	208,198	21.5%	110,486	14.5%	97,712	47.4%		166,952	45.0%	83,437	45.0%	83,515	45.0%	
**PCP Visit 2019**														
No	521,282	53.8%	446,929	58.7%	74,353	36.0%	<0.001	146,967	39.6%	73,675	39.7%	73,292	39.5%	0.20
Yes	447,014	46.2%	315,066	41.3%	131,948	64.0%		224,105	60.4%	111,861	60.3%	112,244	60.5%	
**Continuous Outcomes**														
	**mean**	**SD**	**mean**	**SD**	**mean**	**SD**	**p-value**	**mean**	**SD**	**mean**	**SD**	**mean**	**SD**	**p-value**
**CCI**	0.65	1.39	0.51	1.24	1.17	1.77	<0.001	1.07	1.73	1.08	1.76	1.06	1.70	<0.001

CCI: Charlson Comorbidity Index; PCP: primary care physician; SD: standard deviation.

**Appendix 2—table 26.** Statin User Cohort (All Regions) by BP Use, Patient Characteristics Pre/Post Match of BP Users/Non-users.

	All Statin Users by BP: Unmatched							All Statin Users by BP: Matched						
	**All**		**BP Non-user**		**BP User**		**p-value**	**All**		**BP Non-user**		**BP User**		**p-value**
	**N**	**%**	**N**	**%**	**N**	**%**		**N**	**%**	**N**	**%**	**N**	**%**	
**All Patients**	**1,436,300**	**100.0%**	**1,218,319**	**84.8%**	**217,981**	**15.2%**		**426,960**	**100.0%**	**213,480**	**50.0%**	**213,480**	**50.0%**	
**Age**														
≤20	1,385	0.1%	1,365	0.1%	20	0.0%	<0.001	42	0.0%	22	0.0%	20	0.0%	1.00
21-40	44,389	3.1%	44,042	3.6%	347	0.2%		704	0.2%	357	0.2%	347	0.2%	
41-50	135,867	9.5%	133,850	11.0%	2,017	0.9%		4,033	0.9%	2,016	0.9%	2,017	0.9%	
51-60	356,032	24.8%	333,325	27.4%	22,707	10.4%		45,439	10.6%	22,732	10.6%	22,707	10.6%	
61-70	428,943	29.9%	356,208	29.2%	72,735	33.4%		144,861	33.9%	72,341	33.9%	72,520	34.0%	
71-80	302,081	21.0%	223,651	18.4%	78,430	36.0%		150,527	35.3%	75,316	35.3%	75,211	35.2%	
≥81	167,603	11.7%	125,878	10.3%	41,725	19.1%		81,354	19.1%	40,696	19.1%	40,658	19.0%	
**Gender**														
Female	843,147	58.7%	646,846	53.1%	196,301	90.1%	<0.001	383,586	89.8%	191,786	89.8%	191,800	89.8%	0.94
Male	593,153	41.3%	571,473	46.9%	21,680	9.9%		43,374	10.2%	21,694	10.2%	21,680	10.2%	
**Region**														
Midwest	271,319	18.9%	237,718	19.5%	33,601	15.4%	<0.001	67,050	15.7%	33,525	15.7%	33,525	15.7%	1.00
Northeast	423,934	29.5%	366,936	30.1%	56,998	26.1%		113,308	26.5%	56,654	26.5%	56,654	26.5%	
South	523,112	36.4%	442,996	36.4%	80,116	36.8%		157,838	37.0%	78,919	37.0%	78,919	37.0%	
West	217,935	15.2%	170,669	14.0%	47,266	21.7%		88,764	20.8%	44,382	20.8%	44,382	20.8%	
**Insurance**														
Commercial	587,625	40.9%	533,843	43.8%	53,782	24.7%	<0.001	107,552	25.2%	53,774	25.2%	53,778	25.2%	1.00
Dual	55,356	3.9%	42,041	3.5%	13,315	6.1%		24,380	5.7%	12,183	5.7%	12,197	5.7%	
Medicaid	320,911	22.3%	280,799	23.0%	40,112	18.4%		76,121	17.8%	38,050	17.8%	38,071	17.8%	
Medicare	472,408	32.9%	361,636	29.7%	110,772	50.8%		218,907	51.3%	109,473	51.3%	109,434	51.3%	
**PCP Visit 2019**														
No	507,726	35.3%	430,446	35.3%	77,280	35.5%	0.27	151,395	35.5%	75,614	35.4%	75,781	35.5%	0.59
Yes	928,574	64.7%	787,873	64.7%	140,701	64.5%		275,565	64.5%	137,866	64.6%	137,699	64.5%	
**Continuous Outcomes**														
	**mean**	**SD**	**mean**	**SD**	**mean**	**SD**	**p-value**	**mean**	**SD**	**mean**	**SD**	**mean**	**SD**	**p-value**
**CCI**	1.11	1.75	1.13	1.77	0.95	1.66	<0.001	0.97	1.66	0.97	1.66	0.97	1.67	0.79

BP: bisphosphonate; CCI: Charlson Comorbidity Index; PCP: primary care physician; SD: standard deviation.

**Appendix 2—table 27.** Statin User Cohort (Region=New York State) by BP Use, Patient Characteristics Pre/Post Match of BP Users/Non-users.

	Region=NY Statin Users by BP: Unmatched							Region=NY Statin Users by BP: Matched						
	**All**		**BP Non-user**		**BP User**		**p-value**	**All**		**BP Non-user**		**BP User**		**p-value**
	**N**	**%**	**N**	**%**	**N**	**%**		**N**	**%**	**N**	**%**	**N**	**%**	
**All Patients**	**185,536**	**100.0%**	**161,673**	**87.1%**	**23,863**	**12.9%**		**47,472**	**100.0%**	**23,736**	**50.0%**	**23,736**	**50.0%**	
**Age**														
≤20	67	0.0%	67	0.0%	0	0.0%	<0.001	52	0.1%	26	0.1%	26	0.1%	1.00
21-40	4,511	2.4%	4,485	2.8%	26	0.1%		304	0.6%	152	0.6%	152	0.6%	
41-50	15,440	8.3%	15,288	9.5%	152	0.6%		4,381	9.2%	2,192	9.2%	2,189	9.2%	
51-60	43,068	23.2%	40,879	25.3%	2,189	9.2%		14,717	31.0%	7,358	31.0%	7,359	31.0%	
61-70	53,227	28.7%	45,861	28.4%	7,366	30.9%		18,189	38.3%	9,092	38.3%	9,097	38.3%	
71-80	45,662	24.6%	36,474	22.6%	9,188	38.5%		9,829	20.7%	4,916	20.7%	4,913	20.7%	
≥81	23,561	12.7%	18,619	11.5%	4,942	20.7%		0	0.0%		0.0%		0.0%	
**Gender**														
Female	107,955	58.2%	86,194	53.3%	21,761	91.2%	<0.001	43,265	91.1%	21,631	91.1%	21,634	91.1%	0.96
Male	77,581	41.8%	75,479	46.7%	2,102	8.8%		4,207	8.9%	2,105	8.9%	2,102	8.9%	
**Insurance**														
Commercial	57,917	31.2%	54,411	33.7%	3,506	14.7%	<0.001	7,008	14.8%	3,502	14.8%	3,506	14.8%	1.00
Dual	2,257	1.2%	1,664	1.0%	593	2.5%		1,128	2.4%	564	2.4%	564	2.4%	
Medicaid	41,847	22.6%	37,926	23.5%	3,921	16.4%		7,644	16.1%	3,821	16.1%	3,823	16.1%	
Medicare	83,515	45.0%	67,672	41.9%	15,843	66.4%		31,692	66.8%	15,849	66.8%	15,843	66.7%	
**PCP Visit 2019**														
No	73,292	39.5%	63,797	39.5%	9,495	39.8%	0.33	18,870	39.7%	9,434	39.7%	9,436	39.8%	0.99
Yes	112,244	60.5%	97,876	60.5%	14,368	60.2%		28,602	60.3%	14,302	60.3%	14,300	60.2%	
**Continuous Outcomes**														
	**mean**	**SD**	**mean**	**SD**	**mean**	**SD**	**p-value**	**mean**	**SD**	**mean**	**SD**	**mean**	**SD**	**p-value**
**CCI**	1.06	1.70	1.08	1.71	0.92	1.59	<0.001	0.92	1.58	0.92	1.57	0.93	1.59	0.64

BP: bisphosphonate; CCI: Charlson Comorbidity Index; PCP: primary care physician; SD: standard deviation.

**Appendix 2—table 28.** Statin Non-user Cohort (All Regions) by BP Use, Patient Characteristics Pre/Post Match of BP Users/Non-users.

	All Statin Non-users by BP Use: Unmatched							All Statin Non-users by BP: Matched						
	**All**		**BP Non-users**		**BP Users**		**p-value**	**All**		**BP Non-users**		**BP Users**		**p-value**
	**N**	**%**	**N**	**%**	**N**	**%**		**N**	**%**	**N**	**%**	**N**	**%**	
**All Patients**	**1,436,300**	**100.0%**	**1,311,457**	**91.3%**	**124,843**	**8.7%**		**249,432**	**100.0%**	**124,716**	**50.0%**	**124,716**	**50.0%**	
**Age**														
≤20	1,387	0.1%	1,383	0.1%	4	0.0%	<0.001	6	0.0%	2	0.0%	4	0.0%	0.99
21-40	44,371	3.1%	44,170	3.4%	201	0.2%		413	0.2%	212	0.2%	201	0.2%	
41-50	135,748	9.5%	134,305	10.2%	1,443	1.2%		2,880	1.2%	1,437	1.2%	1,443	1.2%	
51-60	354,449	24.7%	336,779	25.7%	17,670	14.2%		35,335	14.2%	17,665	14.2%	17,670	14.2%	
61-70	428,326	29.8%	381,936	29.1%	46,390	37.2%		92,791	37.2%	46,401	37.2%	46,390	37.2%	
71-80	303,279	21.1%	264,157	20.1%	39,122	31.3%		78,077	31.3%	39,037	31.3%	39,040	31.3%	
≥81	168,740	11.7%	148,727	11.3%	20,013	16.0%		39,930	16.0%	19,962	16.0%	19,968	16.0%	
**Gender**														
Female	839,207	58.4%	727,324	55.5%	111,883	89.6%	<0.001	223,501	89.6%	111,745	89.6%	111,756	89.6%	0.94
Male	597,093	41.6%	584,133	44.5%	12,960	10.4%		25,931	10.4%	12,971	10.4%	12,960	10.4%	
**Region**														
Midwest	271,319	18.9%	249,383	19.0%	21,936	17.6%	<0.001	43,870	17.6%	21,935	17.6%	21,935	17.6%	1.00
Northeast	423,934	29.5%	390,134	29.7%	33,800	27.1%		67,594	27.1%	33,797	27.1%	33,797	27.1%	
South	523,112	36.4%	480,680	36.7%	42,432	34.0%		84,618	33.9%	42,309	33.9%	42,309	33.9%	
West	217,935	15.2%	191,260	14.6%	26,675	21.4%		53,350	21.4%	26,675	21.4%	26,675	21.4%	
**Insurance**														
Commercial	587,847	40.9%	552,487	42.1%	35,360	28.3%	<0.001	70,725	28.4%	35,365	28.4%	35,360	28.4%	1.00
Dual	54,851	3.8%	46,371	3.5%	8,480	6.8%		16,696	6.7%	8,342	6.7%	8,354	6.7%	
Medicaid	320,434	22.3%	296,591	22.6%	23,843	19.1%		47,674	19.1%	23,832	19.1%	23,842	19.1%	
Medicare	473,168	32.9%	416,008	31.7%	57,160	45.8%		114,337	45.8%	57,177	45.8%	57,160	45.8%	
**PCP Visit 2019**														
No	508,587	35.4%	473,241	36.1%	35,346	28.3%	<0.001	70,689	28.3%	35,343	28.3%	35,346	28.3%	0.99
Yes	927,713	64.6%	838,216	63.9%	89,497	71.7%		178,743	71.7%	89,373	71.7%	89,370	71.7%	
**Continuous Outcomes**														
	**mean**	**SD**	**mean**	**SD**	**mean**	**SD**	**p-value**	**mean**	**SD**	**mean**	**SD**	**mean**	**SD**	**p-value**
**CCI**	1.12	1.79	1.13	1.79	1.02	1.86	<0.001	1.02	1.85	1.02	1.84	1.02	1.86	0.49

BP: bisphosphonate; CCI: Charlson Comorbidity Index; PCP: primary care physician; SD: standard deviation.

**Appendix 2—table 29.** Statin Non-user Cohort (Region=New York State) by BP Use, Patient Characteristics Pre/Post Match of BP Users/Non-users.

	Region=NY Statin Non-users by BP: Unmatched							Region=NY Statin Non-users by BP: Matched						
	**All**		**BP Non-users**		**BP Users**		**p-value**	**All**		**BP Non-users**		**BP Users**		**p-value**
	**N**	**%**	**N**	**%**	**N**	**%**		**N**	**%**	**N**	**%**	**N**	**%**	
**All Patients**	**185,536**	**100.0%**	**170,990**	**92.2%**	**14,546**	**7.8%**		**29,042**	**100.0%**	**14,521**	**50.0%**	**14,521**	**50.0%**	
**Age**														
≤20	67	0.0%	67	0.0%	0	0.0%	<0.001	0	0.0%	0	0.0%	0	0.0%	1.00
21-40	4,508	2.4%	4,498	2.6%	10	0.1%		23	0.1%	13	0.1%	10	0.1%	
41-50	15,420	8.3%	15,314	9.0%	106	0.7%		211	0.7%	105	0.7%	106	0.7%	
51-60	43,068	23.2%	41,317	24.2%	1,751	12.0%		3,502	12.1%	1,751	12.1%	1,751	12.1%	
61-70	53,233	28.7%	48,148	28.2%	5,085	35.0%		10,174	35.0%	5,089	35.0%	5,085	35.0%	
71-80	45,675	24.6%	40,731	23.8%	4,944	34.0%		9,877	34.0%	4,937	34.0%	4,940	34.0%	
≥81	23,565	12.7%	20,915	12.2%	2,650	18.2%		5,255	18.1%	2,626	18.1%	2,629	18.1%	
**Gender**														
Female	107,420	57.9%	94,242	55.1%	13,178	90.6%	<0.001	26,304	90.6%	13,151	90.6%	13,153	90.6%	0.97
Male	78,116	42.1%	76,748	44.9%	1,368	9.4%		2,738	9.4%	1,370	9.4%	1,368	9.4%	
**Insurance**														
Commercial	58,206	31.4%	56,313	32.9%	1,893	13.0%	<0.001	3,785	13.0%	1,892	13.0%	1,893	13.0%	0.96
Dual	2,190	1.2%	1,754	1.0%	436	3.0%		883	3.0%	449	3.1%	434	3.0%	
Medicaid	41,703	22.5%	38,177	22.3%	3,526	24.2%		6,994	24.1%	3,491	24.0%	3,503	24.1%	
Medicare	83,437	45.0%	74,746	43.7%	8,691	59.7%		17,380	59.8%	8,689	59.8%	8,691	59.9%	
**PCP Visit 2019**														
No	73,675	39.7%	69,382	40.6%	4,293	29.5%	<0.001	8,564	29.5%	4,280	29.5%	4,284	29.5%	0.96
Yes	111,861	60.3%	101,608	59.4%	10,253	70.5%		20,478	70.5%	10,241	70.5%	10,237	70.5%	
**Continuous Outcomes**														
	**mean**	**SD**	**mean**	**SD**	**mean**	**SD**	**p-value**	**mean**	**SD**	**mean**	**SD**	**mean**	**SD**	**p-value**
**CCI**	1.08	1.76	1.09	1.76	0.95	1.75	<0.001	0.95	1.74	0.95	1.73	0.95	1.75	0.82

BP: bisphosphonate; CCI: Charlson Comorbidity Index; PCP: primary care physician; SD: standard deviation.

**Appendix 2—table 30.** Antihypertensive Cohort (All Regions), Patient Characteristics Pre/Post Match.

	All Observations by Antihypertensive Use: Unmatched							All Observations by Antihypertensive Use: Matched						
	**All**		**HTN Non-users**		**HTN Users**		**p-value**	**All**		**HTN Non-users**		**HTN Users**		**p-value**
	**N**	**%**	**N**	**%**	**N**	**%**		**N**	**%**	**N**	**%**	**N**	**%**	
**All Patients**	**7,906,603**	**100.0%**	**5,805,483**	**73.4%**	**2,101,120**	**26.6%**		**3,572,002**	**100.0%**	**1,786,001**	**50.0%**	**1,786,001**	**50.0%**	
**Age**														
≤20	1,840,050	23.3%	1,823,229	31.4%	16,821	0.8%	<0.001	33,574	0.9%	16,785	0.9%	16,789	0.9%	0.44
21-40	1,446,999	18.3%	1,299,520	22.4%	147,479	7.0%		293,445	8.2%	146,712	8.2%	146,733	8.2%	
41-50	925,309	11.7%	685,931	11.8%	239,378	11.4%		463,130	13.0%	231,312	13.0%	231,818	13.0%	
51-60	1,250,190	15.8%	759,987	13.1%	490,203	23.3%		870,549	24.4%	434,995	24.4%	435,554	24.4%	
61-70	1,181,261	14.9%	626,235	10.8%	555,026	26.4%		918,823	25.7%	459,192	25.7%	459,631	25.7%	
71-80	783,775	9.9%	381,957	6.6%	401,818	19.1%		619,578	17.3%	309,898	17.4%	309,680	17.3%	
≥81	479,019	6.1%	228,624	3.9%	250,395	11.9%		372,903	10.4%	187,107	10.5%	185,796	10.4%	
**Gender**														
Female	4,670,960	59.1%	3,402,357	58.6%	1,268,603	60.4%	<0.001	2,159,365	60.5%	1,079,468	60.4%	1,079,897	60.5%	0.64
Male	3,235,643	40.9%	2,403,126	41.4%	832,517	39.6%		1,412,637	39.5%	706,533	39.6%	706,104	39.5%	
**Region**														
Midwest	1,467,802	18.6%	1,065,772	18.4%	402,030	19.1%	<0.001	694,206	19.4%	347,103	19.4%	347,103	19.4%	1.00
Northeast	2,152,560	27.2%	1,568,239	27.0%	584,321	27.8%		997,132	27.9%	498,566	27.9%	498,566	27.9%	
South	3,042,604	38.5%	2,240,163	38.6%	802,441	38.2%		1,338,570	37.5%	669,285	37.5%	669,285	37.5%	
West	1,243,637	15.7%	931,309	16.0%	312,328	14.9%		542,094	15.2%	271,047	15.2%	271,047	15.2%	
**Insurance**														
Commercial	3,938,603	49.8%	3,060,354	52.7%	878,249	41.8%	<0.001	1,695,516	47.5%	848,106	47.5%	847,410	47.4%	0.80
Dual	156,497	2.0%	55,827	1.0%	100,670	4.8%		93,467	2.6%	46,774	2.6%	46,693	2.6%	
Medicaid	2,594,500	32.8%	2,091,349	36.0%	503,151	23.9%		812,737	22.8%	406,012	22.7%	406,725	22.8%	
Medicare	1,217,003	15.4%	597,953	10.3%	619,050	29.5%		970,282	27.2%	485,109	27.2%	485,173	27.2%	
**PCP Visit 2019**														
No	4,283,697	54.2%	3,531,914	60.8%	751,783	35.8%	<0.001	1,438,005	40.3%	719,756	40.3%	718,249	40.2%	0.10
Yes	3,622,906	45.8%	2,273,569	39.2%	1,349,337	64.2%		2,133,997	59.7%	1,066,245	59.7%	1,067,752	59.8%	
**Continuous Outcomes**														
	**mean**	**SD**	**mean**	**SD**	**mean**	**SD**	**p-value**	**mean**	**SD**	**mean**	**SD**	**mean**	**SD**	**p-value**
**CCI**	0.62	1.38	0.43	1.14	1.13	1.80	<0.001	0.95	1.65	0.96	1.66	0.95	1.64	<0.05

CCI: Charlson Comorbidity Index; HTN: antihypertensive; PCP: primary care physician; SD: standard deviation.

**Appendix 2—table 31.** Antihypertensive Cohort (Region=New York State), Patient Characteristics Pre/Post Match.

	Region=NY by Antihypertensive Use: Unmatched							Region=NY by Antihypertensive Use: Matched						
	**All**		**HTN Non-users**		**HTN Users**		**p-value**	**All**		**HTN Non-users**		**HTN Users**		**p-value**
	**N**	**%**	**N**	**%**	**N**	**%**		**N**	**%**	**N**	**%**	**N**	**%**	
**All Patients**	**968,296**	**100.0%**	**709,644**	**73.3%**	**258,652**	**26.7%**		**407,248**	**100.0%**	**203,624**	**50.0%**	**203,624**	**50.0%**	
**Age**														
≤20	133,178	13.8%	132,352	18.7%	826	0.3%	<0.001	1,622	0.4%	811	0.4%	811	0.4%	1.00
21-40	192,959	19.9%	181,447	25.6%	11,512	4.5%		22,930	5.6%	11,465	5.6%	11,465	5.6%	
41-50	127,794	13.2%	105,490	14.9%	22,304	8.6%		43,846	10.8%	21,923	10.8%	21,923	10.8%	
51-60	172,444	17.8%	119,643	16.9%	52,801	20.4%		96,318	23.7%	48,159	23.7%	48,159	23.7%	
61-70	159,912	16.5%	92,103	13.0%	67,809	26.2%		109,858	27.0%	54,929	27.0%	54,929	27.0%	
71-80	120,117	12.4%	54,076	7.6%	66,041	25.5%		88,734	21.8%	44,367	21.8%	44,367	21.8%	
≥81	61,892	6.4%	24,533	3.5%	37,359	14.4%		43,940	10.8%	21,970	10.8%	21,970	10.8%	
**Gender**														
Female	573,610	59.2%	419,901	59.2%	153,709	59.4%	0.02	240,930	59.2%	120,465	59.2%	120,465	59.2%	1.00
Male	394,686	40.8%	289,743	40.8%	104,943	40.6%		166,318	40.8%	83,159	40.8%	83,159	40.8%	
**Insurance**														
Commercial	500,918	51.7%	425,181	59.9%	75,737	29.3%	<0.001	150,918	37.1%	75,459	37.1%	75,459	37.1%	1.00
Dual	6,814	0.7%	1,659	0.2%	5,155	2.0%		2,986	0.7%	1,493	0.7%	1,493	0.7%	
Medicaid	252,366	26.1%	193,207	27.2%	59,159	22.9%		95,032	23.3%	47,516	23.3%	47,516	23.3%	
Medicare	208,198	21.5%	89,597	12.6%	118,601	45.9%		158,312	38.9%	79,156	38.9%	79,156	38.9%	
**PCP Visit 2019**														
No	521,282	53.8%	423,952	59.7%	97,330	37.6%	<0.001	181,234	44.5%	90,617	44.5%	90,617	44.5%	1.00
Yes	447,014	46.2%	285,692	40.3%	161,322	62.4%		226,014	55.5%	113,007	55.5%	113,007	55.5%	
**Continuous Outcomes**														
	**mean**	**SD**	**mean**	**SD**	**mean**	**SD**	**p-value**	**mean**	**SD**	**mean**	**SD**	**mean**	**SD**	**p-value**
**CCI**	0.65	1.39	0.46	1.16	1.17	1.80	<0.001	0.95	1.60	0.95	1.60	0.95	1.60	1.00

CCI: Charlson Comorbidity Index; HTN: antihypertensive; PCP: primary care physician; SD: standard deviation.

**Appendix 2—table 32.** Antihypertensive User Cohort (All Regions) by BP Use, Patient Characteristics Pre/Post Match of BP Users/Non-users.

	All Antihypertensive Users by BP: Unmatched							All Antihypertensive Users by BP: Matched						
	**All**		**BP Non-user**		**BP User**		**p-value**	**All**		**BP Non-user**		**BP User**		**p-value**
	**N**	**%**	**N**	**%**	**N**	**%**		**N**	**%**	**N**	**%**	**N**	**%**	
**All Patients**	**1,786,001**	**100.0%**	**1,579,388**	**88.4%**	**206,613**	**11.6%**		**408,792**	**100.0%**	**204,396**	**50.0%**	**204,396**	**50.0%**	
**Age**														
≤20	16,789	0.9%	16,586	1.1%	203	0.1%	<0.001	411	0.1%	208	0.1%	203	0.1%	1.00
21-40	146,733	8.2%	145,872	9.2%	861	0.4%		1,728	0.4%	868	0.4%	860	0.4%	
41-50	231,818	13.0%	229,150	14.5%	2,668	1.3%		5,333	1.3%	2,667	1.3%	2,666	1.3%	
51-60	435,554	24.4%	413,155	26.2%	22,399	10.8%		44,796	11.0%	22,399	11.0%	22,397	11.0%	
61-70	459,631	25.7%	390,664	24.7%	68,967	33.4%		137,730	33.7%	68,862	33.7%	68,868	33.7%	
71-80	309,680	17.3%	237,749	15.1%	71,931	34.8%		140,882	34.5%	70,439	34.5%	70,443	34.5%	
≥81	185,796	10.4%	146,212	9.3%	39,584	19.2%		77,912	19.1%	38,953	19.1%	38,959	19.1%	
**Gender**														
Female	1,079,897	60.5%	894,472	56.6%	185,425	89.7%	<0.001	366,424	89.6%	183,212	89.6%	183,212	89.6%	1.00
Male	706,104	39.5%	684,916	43.4%	21,188	10.3%		42,368	10.4%	21,184	10.4%	21,184	10.4%	
**Region**														
Midwest	347,103	19.4%	313,523	19.9%	33,580	16.3%	<0.001	67,058	16.4%	33,529	16.4%	33,529	16.4%	1.00
Northeast	498,566	27.9%	444,828	28.2%	53,738	26.0%		107,150	26.2%	53,575	26.2%	53,575	26.2%	
South	669,285	37.5%	595,410	37.7%	73,875	35.8%		146,890	35.9%	73,445	35.9%	73,445	35.9%	
West	271,047	15.2%	225,627	14.3%	45,420	22.0%		87,694	21.5%	43,847	21.5%	43,847	21.5%	
**Insurance**														
Commercial	847,410	47.4%	787,519	49.9%	59,891	29.0%	<0.001	119,737	29.3%	59,863	29.3%	59,874	29.3%	1.00
Dual	46,693	2.6%	37,153	2.4%	9,540	4.6%		17,884	4.4%	8,945	4.4%	8,939	4.4%	
Medicaid	406,725	22.8%	369,893	23.4%	36,832	17.8%		70,769	17.3%	35,387	17.3%	35,382	17.3%	
Medicare	485,173	27.2%	384,823	24.4%	100,350	48.6%		200,402	49.0%	100,201	49.0%	100,201	49.0%	
**PCP Visit 2019**														
No	718,249	40.2%	633,042	40.1%	85,207	41.2%	<0.001	168,255	41.2%	84,128	41.2%	84,127	41.2%	1.00
Yes	1,067,752	59.8%	946,346	59.9%	121,406	58.8%		240,537	58.8%	120,268	58.8%	120,269	58.8%	
**Continuous Outcomes**														
	**mean**	**SD**	**mean**	**SD**	**mean**	**SD**	**p-value**	**mean**	**SD**	**mean**	**SD**	**mean**	**SD**	**p-value**
**CCI**	0.95	1.64	0.95	1.64	0.94	1.68	0.02	0.95	1.67	0.95	1.67	0.95	1.68	0.68

BP: bisphosphonate; CCI: Charlson Comorbidity Index; PCP: primary care physician; SD: standard deviation.

**Appendix 2—table 33.** Antihypertensive User Cohort (Region=New York State) by BP Use, Patient Characteristics Pre/Post Match of BP Users/Non-users.

	Region=NY Antihypertensive Users by BP: Unmatched							Region=NY Antihypertensive Users by BP: Matched						
	**All**		**BP Non-user**		**BP User**		**p-value**	**All**		**BP Non-user**		**BP User**		**p-value**
	**N**	**%**	**N**	**%**	**N**	**%**		**N**	**%**	**N**	**%**	**N**	**%**	
**All Patients**	**203,624**	**100.0%**	**182,411**	**89.6%**	**21,213**	**10.4%**		**42,252**	**100.0%**	**21,126**	**50.0%**	**21,126**	**50.0%**	
**Age**														
≤20	811	0.4%	798	0.4%	13	0.1%	<0.001	27	0.1%	14	0.1%	13	0.1%	1.00
21-40	11,465	5.6%	11,396	6.2%	69	0.3%		137	0.3%	68	0.3%	69	0.3%	
41-50	21,923	10.8%	21,747	11.9%	176	0.8%		354	0.8%	178	0.8%	176	0.8%	
51-60	48,159	23.7%	46,047	25.2%	2,112	10.0%		4,218	10.0%	2,108	10.0%	2,110	10.0%	
61-70	54,929	27.0%	48,022	26.3%	6,907	32.6%		13,804	32.7%	6,902	32.7%	6,902	32.7%	
71-80	44,367	21.8%	36,409	20.0%	7,958	37.5%		15,777	37.3%	7,886	37.3%	7,891	37.4%	
≥81	21,970	10.8%	17,992	9.9%	3,978	18.8%		7,935	18.8%	3,970	18.8%	3,965	18.8%	
**Gender**														
Female	120,465	59.2%	101,190	55.5%	19,275	90.9%	<0.001	38,380	90.8%	19,190	90.8%	19,190	90.8%	1.00
Male	83,159	40.8%	81,221	44.5%	1,938	9.1%		3,872	9.2%	1,936	9.2%	1,936	9.2%	
**Insurance**														
Commercial	75,459	37.1%	71,460	39.2%	3,999	18.9%	<0.001	7,993	18.9%	3,997	18.9%	3,996	18.9%	1.00
Dual	1,493	0.7%	1,151	0.6%	342	1.6%		643	1.5%	322	1.5%	321	1.5%	
Medicaid	47,516	23.3%	44,248	24.3%	3,268	15.4%		6,414	15.2%	3,207	15.2%	3,207	15.2%	
Medicare	79,156	38.9%	65,552	35.9%	13,604	64.1%		27,202	64.4%	13,600	64.4%	13,602	64.4%	
**PCP Visit 2019**														
No	90,617	44.5%	80,739	44.3%	9,878	46.6%	<0.001	19,672	46.6%	9,837	46.6%	9,835	46.6%	0.98
Yes	113,007	55.5%	101,672	55.7%	11,335	53.4%		22,580	53.4%	11,289	53.4%	11,291	53.4%	
**Continuous Outcomes**														
	**mean**	**SD**	**mean**	**SD**	**mean**	**SD**	**p-value**	**mean**	**SD**	**mean**	**SD**	**mean**	**SD**	**p-value**
**CCI**	0.95	1.60	0.95	1.61	0.88	1.54	<0.001	0.87	1.53	0.87	1.52	0.87	1.53	0.87

BP: bisphosphonate; CCI: Charlson Comorbidity Index; PCP: primary care physician; SD: standard deviation.

**Appendix 2—table 34.** Antihypertensive Non-user Cohort (All Regions) by BP Use, Patient Characteristics Pre/Post Match of BP Users/Non-users.

	All Antihypertensive Non-users by BP: Unmatched							All Antihypertensive Non-users by BP: Matched						
	**All**		**BP Non-user**		**BP User**		**p-value**	**All**		**BP Non-user**		**BP User**		**p-value**
	**N**	**%**	**N**	**%**	**N**	**%**		**N**	**%**	**N**	**%**	**N**	**%**	
**All Patients**	**1,786,001**	**100.0%**	**1,649,985**	**92.4%**	**136,016**	**7.6%**		**271,448**	**100.0%**	**135,724**	**50.0%**	**135,724**	**50.0%**	
**Age**														
≤20	16,785	0.9%	16,767	1.0%	18	0.0%	<0.001	34	0.0%	16	0.0%	18	0.0%	1.00
21-40	146,712	8.2%	146,210	8.9%	502	0.4%		1,009	0.4%	507	0.4%	502	0.4%	
41-50	231,312	13.0%	228,725	13.9%	2,587	1.9%		5,163	1.9%	2,577	1.9%	2,586	1.9%	
51-60	434,995	24.4%	410,636	24.9%	24,359	17.9%		48,700	17.9%	24,349	17.9%	24,351	17.9%	
61-70	459,192	25.7%	404,445	24.5%	54,747	40.3%		109,415	40.3%	54,711	40.3%	54,704	40.3%	
71-80	309,898	17.4%	271,617	16.5%	38,281	28.1%		76,139	28.0%	38,070	28.0%	38,069	28.0%	
≥81	187,107	10.5%	171,585	10.4%	15,522	11.4%		30,988	11.4%	15,494	11.4%	15,494	11.4%	
**Gender**														
Female	1,079,468	60.4%	956,403	58.0%	123,065	90.5%	<0.001	245,537	90.5%	122,762	90.4%	122,775	90.5%	0.93
Male	706,533	39.6%	693,582	42.0%	12,951	9.5%		25,911	9.5%	12,962	9.6%	12,949	9.5%	
**Region**														
Midwest	347,103	19.4%	321,267	19.5%	25,836	19.0%	<0.001	51,638	19.0%	25,819	19.0%	25,819	19.0%	1.00
Northeast	498,566	27.9%	463,273	28.1%	35,293	25.9%		70,544	26.0%	35,272	26.0%	35,272	26.0%	
South	669,285	37.5%	622,064	37.7%	47,221	34.7%		93,980	34.6%	46,990	34.6%	46,990	34.6%	
West	271,047	15.2%	243,381	14.8%	27,666	20.3%		55,286	20.4%	27,643	20.4%	27,643	20.4%	
**Insurance**														
Commercial	848,106	47.5%	798,579	48.4%	49,527	36.4%	<0.001	99,039	36.5%	49,523	36.5%	49,516	36.5%	1.00
Dual	46,774	2.6%	40,212	2.4%	6,562	4.8%		12,645	4.7%	6,319	4.7%	6,326	4.7%	
Medicaid	406,012	22.7%	381,472	23.1%	24,540	18.0%		49,025	18.1%	24,516	18.1%	24,509	18.1%	
Medicare	485,109	27.2%	429,722	26.0%	55,387	40.7%		110,739	40.8%	55,366	40.8%	55,373	40.8%	
**PCP Visit 2019**														
No	719,756	40.3%	676,255	41.0%	43,501	32.0%	<0.001	86,956	32.0%	43,478	32.0%	43,478	32.0%	1.00
Yes	1,066,245	59.7%	973,730	59.0%	92,515	68.0%		184,492	68.0%	92,246	68.0%	92,246	68.0%	
**Continuous Outcomes**														
	**mean**	**SD**	**mean**	**SD**	**mean**	**SD**	**p-value**	**mean**	**SD**	**mean**	**SD**	**mean**	**SD**	**p-value**
**CCI**	0.96	1.66	0.96	1.65	0.88	1.76	<0.001	0.88	1.75	0.88	1.74	0.88	1.75	0.76

BP: bisphosphonate; CCI: Charlson Comorbidity Index; PCP: primary care physician; SD: standard deviation.

**Appendix 2—table 35.** Antihypertensive Non-user Cohort (Region=New York State) by BP Use, Patient Characteristics Pre/Post Match of BP Users/Non-users.

	Region=NY Antihypertensive Non-Users by BP: Unmatched							Region=NY Antihypertensive Non-users by BP: Matched						
	**All**		**BP Non-user**		**BP User**		**p-value**	**All**		**BP Non-user**		**BP User**		**p-value**
	**N**	**%**	**N**	**%**	**N**	**%**		**N**	**%**	**N**	**%**	**N**	**%**	
**All Patients**	**203,624**	**100.0%**	**189,573**	**93.1%**	**14,051**	**6.9%**		**27,966**	**100.0%**	**13,983**	**50.0%**	**13,983**	**50.0%**	
**Age**														
≤20	811	0.4%	810	0.4%	1	0.0%	<0.001	2	0.0%	1	0.0%	1	0.0%	1.00
21-40	11,465	5.6%	11,451	6.0%	14	0.1%		28	0.1%	14	0.1%	14	0.1%	
41-50	21,923	10.8%	21,762	11.5%	161	1.1%		324	1.2%	163	1.2%	161	1.2%	
51-60	48,159	23.7%	46,035	24.3%	2,124	15.1%		4,245	15.2%	2,121	15.2%	2,124	15.2%	
61-70	54,929	27.0%	49,409	26.1%	5,520	39.3%		11,027	39.4%	5,512	39.4%	5,515	39.4%	
71-80	44,367	21.8%	39,789	21.0%	4,578	32.6%		9,054	32.4%	4,528	32.4%	4,526	32.4%	
≥81	21,970	10.8%	20,317	10.7%	1,653	11.8%		3,286	11.7%	1,644	11.8%	1,642	11.7%	
**Gender**														
Female	120,465	59.2%	107,632	56.8%	12,833	91.3%	<0.001	25,530	91.3%	12,764	91.3%	12,766	91.3%	0.97
Male	83,159	40.8%	81,941	43.2%	1,218	8.7%		2,436	8.7%	1,219	8.7%	1,217	8.7%	
**Insurance**														
Commercial	75,459	37.1%	73,115	38.6%	2,344	16.7%	<0.001	4,683	16.7%	2,342	16.7%	2,341	16.7%	1.00
Dual	1,493	0.7%	1,211	0.6%	282	2.0%		554	2.0%	277	2.0%	277	2.0%	
Medicaid	47,516	23.3%	43,809	23.1%	3,707	26.4%		7,295	26.1%	3,648	26.1%	3,647	26.1%	
Medicare	79,156	38.9%	71,438	37.7%	7,718	54.9%		15,434	55.2%	7,716	55.2%	7,718	55.2%	
**PCP Visit 2019**														
No	90,617	44.5%	85,875	45.3%	4,742	33.7%	<0.001	9,461	33.8%	4,728	33.8%	4,733	33.8%	0.95
Yes	113,007	55.5%	103,698	54.7%	9,309	66.3%		18,505	66.2%	9,255	66.2%	9,250	66.2%	
**Continuous Outcomes**														
	**mean**	**SD**	**mean**	**SD**	**mean**	**SD**	**p-value**	**mean**	**SD**	**mean**	**SD**	**mean**	**SD**	**p-value**
**CCI**	0.95	1.60	0.96	1.60	0.81	1.60	<0.001	0.81	1.59	0.81	1.58	0.81	1.59	0.92

BP: bisphosphonate; CCI: Charlson Comorbidity Index; PCP: primary care physician; SD: standard deviation.

**Appendix 2—table 36.** Antidiabetic Cohort (All Regions), Patient Characteristics Pre/Post Match.

	All Observations by Antidiabetic Use: Unmatched							All Observations by Antidiabetic Use: Matched						
	**All**		**DIAB Non-users**		**DIAB Users**		**p-value**	**All**		**DIAB Non-users**		**DIAB Users**		**p-value**
	**N**	**%**	**N**	**%**	**N**	**%**		**N**	**%**	**N**	**%**	**N**	**%**	
**All Patients**	**7,906,603**	**100.0%**	**7,151,351**	**90.4%**	**755,252**	**9.6%**		**1,509,106**	**100.0%**	**754,553**	**50.0%**	**754,553**	**50.0%**	
**Age**														
≤20	1,840,050	23.3%	1,833,838	25.6%	6,212	0.8%	<0.001	12,422	0.8%	6,211	0.8%	6,211	0.8%	1.00
21-40	1,446,999	18.3%	1,389,243	19.4%	57,756	7.6%		115,448	7.7%	57,723	7.6%	57,725	7.7%	
41-50	925,309	11.7%	833,333	11.7%	91,976	12.2%		183,810	12.2%	91,905	12.2%	91,905	12.2%	
51-60	1,250,190	15.8%	1,058,878	14.8%	191,312	25.3%		382,390	25.3%	191,196	25.3%	191,194	25.3%	
61-70	1,181,261	14.9%	973,670	13.6%	207,591	27.5%		414,869	27.5%	207,435	27.5%	207,434	27.5%	
71-80	783,775	9.9%	645,256	9.0%	138,519	18.3%		276,619	18.3%	138,310	18.3%	138,309	18.3%	
≥81	479,019	6.1%	417,133	5.8%	61,886	8.2%		123,548	8.2%	61,773	8.2%	61,775	8.2%	
**Gender**														
Female	4,670,960	59.1%	4,212,086	58.9%	458,874	60.8%	<0.001	916,914	60.8%	458,455	60.8%	458,459	60.8%	0.99
Male	3,235,643	40.9%	2,939,265	41.1%	296,378	39.2%		592,192	39.2%	296,098	39.2%	296,094	39.2%	
**Region**														
Midwest	1,467,802	18.6%	1,333,631	18.6%	134,171	17.8%	<0.001	268,044	17.8%	134,022	17.8%	134,022	17.8%	1.00
Northeast	2,152,560	27.2%	1,935,311	27.1%	217,249	28.8%		434,080	28.8%	217,040	28.8%	217,040	28.8%	
South	3,042,604	38.5%	2,752,618	38.5%	289,986	38.4%		579,562	38.4%	289,781	38.4%	289,781	38.4%	
West	1,243,637	15.7%	1,129,791	15.8%	113,846	15.1%		227,420	15.1%	113,710	15.1%	113,710	15.1%	
**Insurance**														
Commercial	3,938,603	49.8%	3,631,514	50.8%	307,089	40.7%	<0.001	614,045	40.7%	307,022	40.7%	307,023	40.7%	1.00
Dual	156,497	2.0%	113,496	1.6%	43,001	5.7%		85,209	5.6%	42,603	5.6%	42,606	5.6%	
Medicaid	2,594,500	32.8%	2,387,519	33.4%	206,981	27.4%		413,743	27.4%	206,875	27.4%	206,868	27.4%	
Medicare	1,217,003	15.4%	1,018,822	14.2%	198,181	26.2%		396,109	26.2%	198,053	26.2%	198,056	26.2%	
**PCP Visit 2019**														
No	4,283,697	54.2%	4,030,804	56.4%	252,893	33.5%	<0.001	505,500	33.5%	252,752	33.5%	252,748	33.5%	0.99
Yes	3,622,906	45.8%	3,120,547	43.6%	502,359	66.5%		1,003,606	66.5%	501,801	66.5%	501,805	66.5%	
**Continuous Outcomes**														
	**mean**	**SD**	**mean**	**SD**	**mean**	**SD**	**p-value**	**mean**	**SD**	**mean**	**SD**	**mean**	**SD**	**p-value**
**CCI**	0.62	1.38	0.55	1.30	1.25	1.84	<0.001	1.24	1.82	1.24	1.82	1.24	1.82	0.99

CCI: Charlson Comorbidity Index; DIAB: antidiabetic; PCP: primary care physician; SD: standard deviation.

**Appendix 2—table 37.** Antidiabetic Cohort (Region=New York State), Patient Characteristics Pre/Post Match.

	Region=NY by Antidiabetic Use: Unmatched							Region=NY by Antidiabetic Use: Matched						
	**All**		**DIAB Non-users**		**DIAB Users**		**p-value**	**All**		**DIAB Non-users**		**DIAB Users**		**p-value**
	**N**	**%**	**N**	**%**	**N**	**%**		**N**	**%**	**N**	**%**	**N**	**%**	
**All Patients**	**968,296**	**100.0%**	**863,179**	**89.1%**	**105,117**	**10.9%**		**209,382**	**100.0%**	**104,691**	**50.0%**	**104,691**	**50.0%**	
**Age**														
≤20	133,178	13.8%	132,723	15.4%	455	0.4%	<0.001	910	0.4%	455	0.4%	455	0.4%	1.00
21-40	192,959	19.9%	186,785	21.6%	6,174	5.9%		12,328	5.9%	6,164	5.9%	6,164	5.9%	
41-50	127,794	13.2%	117,342	13.6%	10,452	9.9%		20,880	10.0%	10,440	10.0%	10,440	10.0%	
51-60	172,444	17.8%	148,040	17.2%	24,404	23.2%		48,735	23.3%	24,369	23.3%	24,366	23.3%	
61-70	159,912	16.5%	130,968	15.2%	28,944	27.5%		57,638	27.5%	28,819	27.5%	28,819	27.5%	
71-80	120,117	12.4%	95,621	11.1%	24,496	23.3%		48,625	23.2%	24,311	23.2%	24,314	23.2%	
≥81	61,892	6.4%	51,700	6.0%	10,192	9.7%		20,266	9.7%	10,133	9.7%	10,133	9.7%	
**Gender**														
Female	573,610	59.2%	512,889	59.4%	60,721	57.8%	<0.001	120,937	57.8%	60,467	57.8%	60,470	57.8%	0.99
Male	394,686	40.8%	350,290	40.6%	44,396	42.2%		88,445	42.2%	44,224	42.2%	44,221	42.2%	
**Insurance**														
Commercial	500,918	51.7%	468,804	54.3%	32,114	30.6%	<0.001	64,200	30.7%	32,100	30.7%	32,100	30.7%	1.00
Dual	6,814	0.7%	4,408	0.5%	2,406	2.3%		4,389	2.1%	2,196	2.1%	2,193	2.1%	
Medicaid	252,366	26.1%	224,334	26.0%	28,032	26.7%		55,853	26.7%	27,925	26.7%	27,928	26.7%	
Medicare	208,198	21.5%	165,633	19.2%	42,565	40.5%		84,940	40.6%	42,470	40.6%	42,470	40.6%	
**PCP Visit 2019**														
No	521,282	53.8%	484,071	56.1%	37,211	35.4%	<0.001	74,215	35.4%	37,106	35.4%	37,109	35.4%	0.99
Yes	447,014	46.2%	379,108	43.9%	67,906	64.6%		135,167	64.6%	67,585	64.6%	67,582	64.6%	
**Continuous Outcomes**														
	**mean**	**SD**	**mean**	**SD**	**mean**	**SD**	**p-value**	**mean**	**SD**	**mean**	**SD**	**mean**	**SD**	**p-value**
**CCI**	0.65	1.39	0.56	1.30	1.34	1.84	<0.001	1.32	1.79	1.32	1.79	1.32	1.79	0.98

CCI: Charlson Comorbidity Index; DIAB: antidiabetic; PCP: primary care physician; SD: standard deviation.

**Appendix 2—table 38.** Antidiabetic User Cohort (All Regions) by BP Use, Patient Characteristics Pre/Post Match of BP Users/Non users.

	All Antidiabetic Users by BP: Unmatched							All Antidiabetic Users by BP: Matched						
	**All**		**BP Non-user**		**BP User**		**p-value**	**All**		**BP Non-user**		**BP User**		**p-value**
	**N**	**%**	**N**	**%**	**N**	**%**		**N**	**%**	**N**	**%**	**N**	**%**	
**All Patients**	**754,553**	**100.0%**	**674,024**	**89.3%**	**80,529**	**10.7%**		**159,000**	**100.0%**	**79,500**	**50.0%**	**79,500**	**50.0%**	
**Age**														
≤20	6,211	0.8%	6,169	0.9%	42	0.1%	<0.001	83	0.1%	41	0.1%	42	0.1%	1.00
21-40	57,725	7.7%	57,535	8.5%	190	0.2%		380	0.2%	190	0.2%	190	0.2%	
41-50	91,905	12.2%	90,952	13.5%	953	1.2%		1,905	1.2%	952	1.2%	953	1.2%	
51-60	191,194	25.3%	182,922	27.1%	8,272	10.3%		16,536	10.4%	8,268	10.4%	8,268	10.4%	
61-70	207,434	27.5%	180,895	26.8%	26,539	33.0%		53,028	33.4%	26,512	33.3%	26,516	33.4%	
71-80	138,309	18.3%	107,467	15.9%	30,842	38.3%		60,240	37.9%	30,121	37.9%	30,119	37.9%	
≥81	61,775	8.2%	48,084	7.1%	13,691	17.0%		26,828	16.9%	13,416	16.9%	13,412	16.9%	
**Gender**														
Female	458,459	60.8%	386,400	57.3%	72,059	89.5%	<0.001	142,068	89.4%	71,027	89.3%	71,041	89.4%	0.91
Male	296,094	39.2%	287,624	42.7%	8,470	10.5%		16,932	10.6%	8,473	10.7%	8,459	10.6%	
**Region**														
Midwest	134,022	17.8%	123,909	18.4%	10,113	12.6%	<0.001	20,168	12.7%	10,084	12.7%	10,084	12.7%	1.00
Northeast	217,040	28.8%	196,723	29.2%	20,317	25.2%		40,446	25.4%	20,223	25.4%	20,223	25.4%	
South	289,781	38.4%	257,599	38.2%	32,182	40.0%		63,740	40.1%	31,870	40.1%	31,870	40.1%	
West	113,710	15.1%	95,793	14.2%	17,917	22.2%		34,646	21.8%	17,323	21.8%	17,323	21.8%	
**Insurance**														
Commercial	307,023	40.7%	290,957	43.2%	16,066	20.0%	<0.001	32,086	20.2%	16,043	20.2%	16,043	20.2%	1.00
Dual	42,606	5.6%	32,797	4.9%	9,809	12.2%		18,653	11.7%	9,321	11.7%	9,332	11.7%	
Medicaid	206,868	27.4%	188,638	28.0%	18,230	22.6%		35,513	22.3%	17,759	22.3%	17,754	22.3%	
Medicare	198,056	26.2%	161,632	24.0%	36,424	45.2%		72,748	45.8%	36,377	45.8%	36,371	45.7%	
**PCP Visit 2019**														
No	252,748	33.5%	228,203	33.9%	24,545	30.5%	<0.001	48,374	30.4%	24,184	30.4%	24,190	30.4%	0.97
Yes	501,805	66.5%	445,821	66.1%	55,984	69.5%		110,626	69.6%	55,316	69.6%	55,310	69.6%	
**Continuous Outcomes**														
	**mean**	**SD**	**mean**	**SD**	**mean**	**SD**	**p-value**	**mean**	**SD**	**mean**	**SD**	**mean**	**SD**	**p-value**
**CCI**	1.24	1.82	1.23	1.81	1.32	1.90	<0.001	1.31	1.88	1.31	1.87	1.32	1.88	0.75

BP: bisphosphonate; CCI: Charlson Comorbidity Index; PCP: primary care physician; SD: standard deviation.

**Appendix 2—table 39.** Influences on exploratory choice including WASI scores.

	Region=NY Antidiabetic Users by BP: Unmatched							Region=NY Antidiabetic Users by BP: Matched						
	**All**		**BP Non-user**		**BP User**		**p-value**	**All**		**BP Non-user**		**BP User**		**p-value**
	**N**	**%**	**N**	**%**	**N**	**%**		**N**	**%**	**N**	**%**	**N**	**%**	
**All Patients**	**104,691**	**100.0%**	**95,162**	**90.9%**	**9,529**	**9.1%**		**18,912**	**100.0%**	**9,456**	**50.0%**	**9,456**	**50.0%**	
**Age**														
≤20	455	0.4%	454	0.5%	1	0.0%	<0.001	2	0.0%	1	0.0%	1	0.0%	1.00
21-40	6,164	5.9%	6,152	6.5%	12	0.1%		25	0.1%	13	0.1%	12	0.1%	
41-50	10,440	10.0%	10,363	10.9%	77	0.8%		151	0.8%	75	0.8%	76	0.8%	
51-60	24,366	23.3%	23,532	24.7%	834	8.8%		1,665	8.8%	831	8.8%	834	8.8%	
61-70	28,819	27.5%	25,939	27.3%	2,880	30.2%		5,741	30.4%	2,870	30.4%	2,871	30.4%	
71-80	24,314	23.2%	20,338	21.4%	3,976	41.7%		7,880	41.7%	3,941	41.7%	3,939	41.7%	
≥81	10,133	9.7%	8,384	8.8%	1,749	18.4%		3,448	18.2%	1,725	18.2%	1,723	18.2%	
**Gender**														
Female	60,470	57.8%	51,884	54.5%	8,586	90.1%	<0.001	17,022	90.0%	8,509	90.0%	8,513	90.0%	0.92
Male	44,221	42.2%	43,278	45.5%	943	9.9%		1,890	10.0%	947	10.0%	943	10.0%	
**Insurance**														
Commercial	32,100	30.7%	31,172	32.8%	928	9.7%	<0.001	1,849	9.8%	924	9.8%	925	9.8%	1.00
Dual	2,193	2.1%	1,693	1.8%	500	5.2%		978	5.2%	490	5.2%	488	5.2%	
Medicaid	27,928	26.7%	25,978	27.3%	1,950	20.5%		3,793	20.1%	1,897	20.1%	1,896	20.1%	
Medicare	42,470	40.6%	36,319	38.2%	6,151	64.6%		12,292	65.0%	6,145	65.0%	6,147	65.0%	
**PCP Visit 2019**														
No	37,109	35.4%	33,894	35.6%	3,215	33.7%	<.001	6,363	33.6%	3,182	33.7%	3,181	33.6%	0.99
Yes	67,582	64.6%	61,268	64.4%	6,314	66.3%		12,549	66.4%	6,274	66.3%	6,275	66.4%	
**Continuous Outcomes**														
	**mean**	**SD**	**mean**	**SD**	**mean**	**SD**	**p-value**	**mean**	**SD**	**mean**	**SD**	**mean**	**SD**	**p-value**
**CCI**	1.32	1.79	1.31	1.79	1.46	1.87	<0.001	1.44	1.83	1.44	1.82	1.45	1.84	0.75

BP: bisphosphonate; CCI: Charlson Comorbidity Index; PCP: primary care physician; SD: standard deviation.

**Appendix 2—table 40.** Antidiabetic Non-user Cohort (All Regions) by BP Use, Patient Characteristics Pre/Post Match of BP Users/Non-users.

	All Antidiabetic Non-users by BP: Unmatched							All Antidiabetic Non-users by BP: Matched						
	**All**		**BP Non-user**		**BP User**		**p-value**	**All**		**BP Non-user**		**BP User**		**p-value**
	**N**	**%**	**N**	**%**	**N**	**%**		**N**	**%**	**N**	**%**	**N**	**%**	
**All Patients**	**754,553**	**100.0%**	**681,380**	**90.3%**	**73,173**	**9.7%**		**145,028**	**100.0%**	**72,514**	**50.0%**	**72,514**	**50.0%**	
**Age**														
≤20	6,211	0.8%	6,199	0.9%	12	0.0%	<0.001	24	0.0%	12	0.0%	12	0.0%	1.00
21-40	57,723	7.6%	57,497	8.4%	226	0.3%		455	0.3%	229	0.3%	226	0.3%	
41-50	91,905	12.2%	90,693	13.3%	1,212	1.7%		2,421	1.7%	1,209	1.7%	1,212	1.7%	
51-60	191,196	25.3%	180,332	26.5%	10,864	14.8%		21,721	15.0%	10,860	15.0%	10,861	15.0%	
61-70	207,435	27.5%	180,825	26.5%	26,610	36.4%		53,115	36.6%	26,558	36.6%	26,557	36.6%	
71-80	138,310	18.3%	114,018	16.7%	24,292	33.2%		47,723	32.9%	23,861	32.9%	23,862	32.9%	
≥81	61,773	8.2%	51,816	7.6%	9,957	13.6%		19,569	13.5%	9,785	13.5%	9,784	13.5%	
**Gender**														
Female	458,455	60.8%	393,376	57.7%	65,079	88.9%	<0.001	128,836	88.8%	64,411	88.8%	64,425	88.8%	0.91
Male	296,098	39.2%	288,004	42.3%	8,094	11.1%		16,192	11.2%	8,103	11.2%	8,089	11.2%	
**Region**														
Midwest	134,022	17.8%	123,283	18.1%	10,739	14.7%	<0.001	21,390	14.7%	10,695	14.7%	10,695	14.7%	1.00
Northeast	217,040	28.8%	197,710	29.0%	19,330	26.4%		38,510	26.6%	19,255	26.6%	19,255	26.6%	
South	289,781	38.4%	261,382	38.4%	28,399	38.8%		55,812	38.5%	27,906	38.5%	27,906	38.5%	
West	113,710	15.1%	99,005	14.5%	14,705	20.1%		29,316	20.2%	14,658	20.2%	14,658	20.2%	
**Insurance**														
Commercial	307,022	40.7%	289,018	42.4%	18,004	24.6%	<0.001	35,983	24.8%	17,988	24.8%	17,995	24.8%	1.00
Dual	42,603	5.6%	33,444	4.9%	9,159	12.5%		17,221	11.9%	8,611	11.9%	8,610	11.9%	
Medicaid	206,875	27.4%	190,166	27.9%	16,709	22.8%		33,264	22.9%	16,636	22.9%	16,628	22.9%	
Medicare	198,053	26.2%	168,752	24.8%	29,301	40.0%		58,560	40.4%	29,279	40.4%	29,281	40.4%	
**PCP Visit 2019**														
No	252,752	33.5%	233,775	34.3%	18,977	25.9%	<0.001	37,812	26.1%	18,903	26.1%	18,909	26.1%	0.97
Yes	501,801	66.5%	447,605	65.7%	54,196	74.1%		107,216	73.9%	53,611	73.9%	53,605	73.9%	
**Continuous Outcomes**														
	**mean**	**SD**	**mean**	**SD**	**mean**	**SD**	**p-value**	**mean**	**SD**	**mean**	**SD**	**mean**	**SD**	**p-value**
**CCI**	1.24	1.82	1.24	1.81	1.24	1.89	0.92	1.24	1.87	1.24	1.87	1.25	1.88	0.63

BP: bisphosphonate; CCI: Charlson Comorbidity Index; PCP: primary care physician; SD: standard deviation.

**Appendix 2—table 41.** Antidiabetic Non-user Cohort (Region=New York State) by BP Use, Patient Characteristics Pre/Post Match of BP Users/Non-users.

	Region=NY Antidiabetic Non-users by BP: Unmatched							Region=NY Antidiabetic Non-users by BP: Matched						
	**All**		**BP Non-user**		**BP User**		**p-value**	**All**		**BP Non-user**		**BP User**		**p-value**
	**N**	**%**	**N**	**%**	**N**	**%**		**N**	**%**	**N**	**%**	**N**	**%**	
**All Patients**	**104,691**	**100.0%**	**95,416**	**91.1%**	**9,275**	**8.9%**		**18,288**	**100.0%**	**9,144**	**50.0%**	**9,144**	**50.0%**	
**Age**														
≤20	455	0.4%	455	0.5%	0	0.0%	<0.001	0	0.0%	0	0.0%	0	0.0%	1.00
21-40	6,164	5.9%	6,146	6.4%	18	0.2%		36	0.2%	18	0.2%	18	0.2%	
41-50	10,440	10.0%	10,367	10.9%	73	0.8%		147	0.8%	74	0.8%	73	0.8%	
51-60	24,369	23.3%	23,304	24.4%	1,065	11.5%		2,128	11.6%	1,064	11.6%	1,064	11.6%	
61-70	28,819	27.5%	25,720	27.0%	3,099	33.4%		6,190	33.8%	3,097	33.9%	3,093	33.8%	
71-80	24,311	23.2%	20,826	21.8%	3,485	37.6%		6,839	37.4%	3,419	37.4%	3,420	37.4%	
≥81	10,133	9.7%	8,598	9.0%	1,535	16.5%		2,948	16.1%	1,472	16.1%	1,476	16.1%	
**Gender**														
Female	60,467	57.8%	52,194	54.7%	8,273	89.2%	<0.001	16,291	89.1%	8,146	89.1%	8,145	89.1%	0.98
Male	44,224	42.2%	43,222	45.3%	1,002	10.8%		1,997	10.9%	998	10.9%	999	10.9%	
**Insurance**														
Commercial	32,100	30.7%	31,095	32.6%	1,005	10.8%	<0.001	2,002	10.9%	1,000	10.9%	1,002	11.0%	1.00
Dual	2,196	2.1%	1,675	1.8%	521	5.6%		1,006	5.5%	502	5.5%	504	5.5%	
Medicaid	27,925	26.7%	25,530	26.8%	2,395	25.8%		4,575	25.0%	2,289	25.0%	2,286	25.0%	
Medicare	42,470	40.6%	37,116	38.9%	5,354	57.7%		10,705	58.5%	5,353	58.5%	5,352	58.5%	
**PCP Visit 2019**														
No	37,106	35.4%	34,553	36.2%	2,553	27.5%	<0.001	5,039	27.6%	2,518	27.5%	2,521	27.6%	0.96
Yes	67,585	64.6%	60,863	63.8%	6,722	72.5%		13,249	72.4%	6,626	72.5%	6,623	72.4%	
**Continuous Outcomes**														
	**mean**	**SD**	**mean**	**SD**	**mean**	**SD**	**p-value**	**mean**	**SD**	**mean**	**SD**	**mean**	**SD**	**p-value**
**CCI**	1.32	1.79	1.32	1.79	1.37	1.81	0.007	1.37	1.78	1.36	1.78	1.37	1.79	0.92

BP: bisphosphonate; CCI: Charlson Comorbidity Index; PCP: primary care physician; SD: standard deviation.

**Appendix 2—table 42.** Antidepressant Cohort (All Regions), Patient Characteristics Pre/Post Match.

	All Observations by Antidepressant Use: Unmatched							All Observations by Antidepressant Use: Matched						
	**All**		**DEPR Non-users**		**DEPR Users**		**p-value**	**All**		**DEPR Non-users**		**DEPR Users**		**p-value**
	**N**	**%**	**N**	**%**	**N**	**%**		**N**	**%**	**N**	**%**	**N**	**%**	
**All Patients**	**7,906,603**	**100.0%**	**6,335,598**	**80.1%**	**1,571,005**	**19.9%**		**3,072,096**	**100.0%**	**1,536,048**	**50.0%**	**1,536,048**	**50.0%**	
**Age**														
≤20	1,840,050	23.3%	1,750,435	27.6%	89,615	5.7%	<0.001	179,136	5.8%	89,565	5.8%	89,571	5.8%	1.00
21-40	1,446,999	18.3%	1,128,316	17.8%	318,683	20.3%		631,186	20.5%	315,593	20.5%	315,593	20.5%	
41-50	925,309	11.7%	683,455	10.8%	241,854	15.4%		466,681	15.2%	233,336	15.2%	233,345	15.2%	
51-60	1,250,190	15.8%	899,512	14.2%	350,678	22.3%		667,305	21.7%	333,650	21.7%	333,655	21.7%	
61-70	1,181,261	14.9%	879,560	13.9%	301,701	19.2%		592,345	19.3%	296,182	19.3%	296,163	19.3%	
71-80	783,775	9.9%	613,922	9.7%	169,853	10.8%		338,594	11.0%	169,295	11.0%	169,299	11.0%	
≥81	479,019	6.1%	380,398	6.0%	98,621	6.3%		196,849	6.4%	98,427	6.4%	98,422	6.4%	
**Gender**														
Female	4,670,960	59.1%	3,527,859	55.7%	1,143,101	72.8%	<0.001	2,219,179	72.2%	1,109,580	72.2%	1,109,599	72.2%	0.98
Male	3,235,643	40.9%	2,807,739	44.3%	427,904	27.2%		852,917	27.8%	426,468	27.8%	426,449	27.8%	
**Region**														
Midwest	1,467,802	18.6%	1,120,969	17.7%	346,833	22.1%	<0.001	671,016	21.8%	335,508	21.8%	335,508	21.8%	1.00
Northeast	2,152,560	27.2%	1,765,134	27.9%	387,426	24.7%		766,046	24.9%	383,023	24.9%	383,023	24.9%	
South	3,042,604	38.5%	2,428,383	38.3%	614,221	39.1%		1,192,058	38.8%	596,029	38.8%	596,029	38.8%	
West	1,243,637	15.7%	1,021,112	16.1%	222,525	14.2%		442,976	14.4%	221,488	14.4%	221,488	14.4%	
**Insurance**														
Commercial	3,938,603	49.8%	3,230,475	51.0%	708,128	45.1%	<0.001	1,415,351	46.1%	707,675	46.1%	707,676	46.1%	1.00
Dual	156,497	2.0%	94,682	1.5%	61,815	3.9%		109,676	3.6%	54,836	3.6%	54,840	3.6%	
Medicaid	2,594,500	32.8%	2,083,688	32.9%	510,812	32.5%		972,897	31.7%	486,446	31.7%	486,451	31.7%	
Medicare	1,217,003	15.4%	926,753	14.6%	290,250	18.5%		574,172	18.7%	287,091	18.7%	287,081	18.7%	
**PCP Visit 2019**														
No	4,283,697	54.2%	3,672,879	58.0%	610,818	38.9%	<0.001	1,210,520	39.4%	605,256	39.4%	605,264	39.4%	0.99
Yes	3,622,906	45.8%	2,662,719	42.0%	960,187	61.1%		1,861,576	60.6%	930,792	60.6%	930,784	60.6%	
**Continuous Outcomes**														
	**mean**	**SD**	**mean**	**SD**	**mean**	**SD**	**p-value**	**mean**	**SD**	**mean**	**SD**	**mean**	**SD**	**p-value**
**CCI**	0.62	1.38	0.55	1.29	0.90	1.65	<0.001	0.87	1.60	0.87	1.60	0.87	1.60	0.98

CCI: Charlson Comorbidity Index; DEPR: antidepressant; PCP: primary care physician; SD: standard deviation.

**Appendix 2—table 43.** Antidepressant Cohort (Region=New York State), Patient Characteristics Pre/Post Match.

	Region=NY by Antidepressant Use: Unmatched							Region=NY by Antidepressant Use: Matched						
	**All**		**DEPR Non-users**		**DEPR Users**		**p-value**	**All**		**DEPR Non-users**		**DEPR Users**		**p-value**
	**N**	**%**	**N**	**%**	**N**	**%**		**N**	**%**	**N**	**%**	**N**	**%**	
**All Patients**	**968,296**	**100.0%**	**832,215**	**85.9%**	**136,081**	**14.1%**		**271,032**	**100.0%**	**135,516**	**50.0%**	**135,516**	**50.0%**	
**Age**														
≤20	133,178	13.8%	128,810	15.5%	4,368	3.2%	<0.001	8,728	3.2%	4,365	3.2%	4,363	3.2%	1.00
21-40	192,959	19.9%	170,076	20.4%	22,883	16.8%		45,666	16.8%	22,832	16.8%	22,834	16.8%	
41-50	127,794	13.2%	109,184	13.1%	18,610	13.7%		36,965	13.6%	18,483	13.6%	18,482	13.6%	
51-60	172,444	17.8%	142,702	17.1%	29,742	21.9%		58,966	21.8%	29,481	21.8%	29,485	21.8%	
61-70	159,912	16.5%	132,317	15.9%	27,595	20.3%		55,083	20.3%	27,543	20.3%	27,540	20.3%	
71-80	120,117	12.4%	99,040	11.9%	21,077	15.5%		42,076	15.5%	21,038	15.5%	21,038	15.5%	
≥81	61,892	6.4%	50,086	6.0%	11,806	8.7%		23,548	8.7%	11,774	8.7%	11,774	8.7%	
**Gender**														
Female	573,610	59.2%	476,684	57.3%	96,926	71.2%	<0.001	192,930	71.2%	96,468	71.2%	96,462	71.2%	0.98
Male	394,686	40.8%	355,531	42.7%	39,155	28.8%		78,102	28.8%	39,048	28.8%	39,054	28.8%	
**Insurance**														
Commercial	500,918	51.7%	449,071	54.0%	51,847	38.1%	<0.001	103,658	38.2%	51,829	38.2%	51,829	38.2%	1.00
Dual	6,814	0.7%	5,072	0.6%	1,742	1.3%		3,191	1.2%	1,591	1.2%	1,600	1.2%	
Medicaid	252,366	26.1%	213,705	25.7%	38,661	28.4%		77,136	28.5%	38,569	28.5%	38,567	28.5%	
Medicare	208,198	21.5%	164,367	19.8%	43,831	32.2%		87,047	32.1%	43,527	32.1%	43,520	32.1%	
**PCP Visit 2019**														
No	521,282	53.8%	467,739	56.2%	53,543	39.3%	<0.001	106,797	39.4%	53,397	39.4%	53,400	39.4%	0.99
Yes	447,014	46.2%	364,476	43.8%	82,538	60.7%		164,235	60.6%	82,119	60.6%	82,116	60.6%	
**Continuous Outcomes**														
	**mean**	**SD**	**mean**	**SD**	**mean**	**SD**	**p-value**	**mean**	**SD**	**mean**	**SD**	**mean**	**SD**	**p-value**
**CCI**	0.65	1.39	0.59	1.32	0.98	1.71	<0.001	0.96	1.68	0.96	1.68	0.96	1.68	0.99

CCI: Charlson Comorbidity Index; DEPR: antidepressant; PCP: primary care physician; SD: standard deviation.

**Appendix 2—table 44.** Antidepressant User Cohort (All Regions) by BP Use, Patient Characteristics Pre/Post Match of BP Users/Non-users.

	All Antidepressant Users by BP: Unmatched							All Antidepressant Users by BP: Matched						
	**All**		**BP Non-user**		**BP User**		**p-value**	**All**		**BP Non-user**		**BP User**		**p-value**
	**N**	**%**	**N**	**%**	**N**	**%**		**N**	**%**	**N**	**%**	**N**	**%**	
**All Patients**	**1,536,048**	**100.0%**	**1,390,939**	**90.6%**	**145,109**	**9.4%**		**288,564**	**100.0%**	**144,282**	**50.0%**	**144,282**	**50.0%**	
**Age**														
≤20	89,571	5.8%	89,415	6.4%	156	0.1%	<0.001	313	0.1%	157	0.1%	156	0.1%	1.00
21-40	315,593	20.5%	314,429	22.6%	1,164	0.8%		2,326	0.8%	1,162	0.8%	1,164	0.8%	
41-50	233,345	15.2%	229,878	16.5%	3,467	2.4%		6,933	2.4%	3,467	2.4%	3,466	2.4%	
51-60	333,655	21.7%	310,316	22.3%	23,339	16.1%		46,674	16.2%	23,339	16.2%	23,335	16.2%	
61-70	296,163	19.3%	244,247	17.6%	51,916	35.8%		103,798	36.0%	51,905	36.0%	51,893	36.0%	
71-80	169,299	11.0%	126,089	9.1%	43,210	29.8%		85,292	29.6%	42,643	29.6%	42,649	29.6%	
≥81	98,422	6.4%	76,565	5.5%	21,857	15.1%		43,228	15.0%	21,609	15.0%	21,619	15.0%	
**Gender**														
Female	1,109,599	72.2%	976,214	70.2%	133,385	91.9%	<0.001	265,123	91.9%	132,553	91.9%	132,570	91.9%	0.91
Male	426,449	27.8%	414,725	29.8%	11,724	8.1%		23,441	8.1%	11,729	8.1%	11,712	8.1%	
**Region**														
Midwest	335,508	21.8%	309,597	22.3%	25,911	17.9%	<0.001	51,754	17.9%	25,877	17.9%	25,877	17.9%	1.00
Northeast	383,023	24.9%	347,944	25.0%	35,079	24.2%		70,010	24.3%	35,005	24.3%	35,005	24.3%	
South	596,029	38.8%	540,382	38.9%	55,647	38.3%		110,518	38.3%	55,259	38.3%	55,259	38.3%	
West	221,488	14.4%	193,016	13.9%	28,472	19.6%		56,282	19.5%	28,141	19.5%	28,141	19.5%	
**Insurance**														
Commercial	707,676	46.1%	664,625	47.8%	43,051	29.7%	<0.001	86,053	29.8%	43,023	29.8%	43,030	29.8%	1.00
Dual	54,840	3.6%	43,171	3.1%	11,669	8.0%		22,384	7.8%	11,193	7.8%	11,191	7.8%	
Medicaid	486,451	31.7%	457,656	32.9%	28,795	19.8%		56,959	19.7%	28,479	19.7%	28,480	19.7%	
Medicare	287,081	18.7%	225,487	16.2%	61,594	42.4%		123,168	42.7%	61,587	42.7%	61,581	42.7%	
**PCP Visit 2019**														
No	605,264	39.4%	553,886	39.8%	51,378	35.4%	<0.001	102,148	35.4%	51,064	35.4%	51,084	35.4%	0.94
Yes	930,784	60.6%	837,053	60.2%	93,731	64.6%		186,416	64.6%	93,218	64.6%	93,198	64.6%	
**Continuous Outcomes**														
	**mean**	**SD**	**mean**	**SD**	**mean**	**SD**	**p-value**	**mean**	**SD**	**mean**	**SD**	**mean**	**SD**	**p-value**
**CCI**	0.87	1.60	0.84	1.58	1.09	1.81	<0.001	1.09	1.79	1.08	1.78	1.09	1.79	0.56

BP: bisphosphonate; CCI: Charlson Comorbidity Index; PCP: primary care physician; SD: standard deviation.

**Appendix 2—table 45.** Antidepressant User Cohort (Region=New York State) by BP Use, Patient Characteristics Pre/Post Match of BP Users/Non-users.

	Region=NY Antidepressant Users by BP: Unmatched							Region=NY Antidepressant Users by BP: Matched						
	**All**		**BP Non-user**		**BP User**		**p-value**	**All**		**BP Non-user**		**BP User**		**p-value**
	**N**	**%**	**N**	**%**	**N**	**%**		**N**	**%**	**N**	**%**	**N**	**%**	
**All Patients**	**135,516**	**100.0%**	**122,566**	**90.4%**	**12,950**	**9.6%**		**25,718**	**100.0%**	**12,859**	**50.0%**	**12,859**	**50.0%**	
**Age**														
≤20	4,363	3.2%	4,357	3.6%	6	0.0%	<0.001	12	0.0%	6	0.0%	6	0.0%	1.00
21-40	22,834	16.8%	22,770	18.6%	64	0.5%		126	0.5%	62	0.5%	64	0.5%	
41-50	18,482	13.6%	18,263	14.9%	219	1.7%		440	1.7%	221	1.7%	219	1.7%	
51-60	29,485	21.8%	27,702	22.6%	1,783	13.8%		3,570	13.9%	1,788	13.9%	1,782	13.9%	
61-70	27,540	20.3%	23,385	19.1%	4,155	32.1%		8,292	32.2%	4,146	32.2%	4,146	32.2%	
71-80	21,038	15.5%	16,548	13.5%	4,490	34.7%		8,863	34.5%	4,430	34.5%	4,433	34.5%	
≥81	11,774	8.7%	9,541	7.8%	2,233	17.2%		4,415	17.2%	2,206	17.2%	2,209	17.2%	
**Gender**														
Female	96,462	71.2%	84,469	68.9%	11,993	92.6%	<0.001	23,810	92.6%	11,906	92.6%	11,904	92.6%	0.96
Male	39,054	28.8%	38,097	31.1%	957	7.4%		1,908	7.4%	953	7.4%	955	7.4%	
**Insurance**														
Commercial	51,829	38.2%	49,332	40.2%	2,497	19.3%	<0.001	4,991	19.4%	2,495	19.4%	2,496	19.4%	1.00
Dual	1,600	1.2%	1,221	1.0%	379	2.9%		710	2.8%	356	2.8%	354	2.8%	
Medicaid	38,567	28.5%	36,366	29.7%	2,201	17.0%		4,269	16.6%	2,131	16.6%	2,138	16.6%	
Medicare	43,520	32.1%	35,647	29.1%	7,873	60.8%		15,748	61.2%	7,877	61.3%	7,871	61.2%	
**PCP Visit 2019**														
No	53,400	39.4%	48,911	39.9%	4,489	34.7%	<0.001	8,901	34.6%	4,449	34.6%	4,452	34.6%	0.97
Yes	82,116	60.6%	73,655	60.1%	8,461	65.3%		16,817	65.4%	8,410	65.4%	8,407	65.4%	
**Continuous Outcomes**														
	**mean**	**SD**	**mean**	**SD**	**mean**	**SD**	**p-value**	**mean**	**SD**	**mean**	**SD**	**mean**	**SD**	**p-value**
**CCI**	0.96	1.68	0.95	1.66	1.13	1.78	<0.001	1.12	1.76	1.12	1.75	1.12	1.77	0.86

BP: bisphosphonate; CCI: Charlson Comorbidity Index; PCP: primary care physician; SD: standard deviation.

**Appendix 2—table 46.** Antidepressant Non-user Cohort (All Regions) by BP Use, Patient Characteristics Pre/Post Match of BP Users/Non-users.

	All Antidepressant Non-users by BP: Unmatched							All Antidepressant Non-users by BP: Matched						
	**All**		**BP Non-user**		**BP User**		**p-value**	**All**		**BP Non-user**		**BP User**		**p-value**
	**N**	**%**	**N**	**%**	**N**	**%**		**N**	**%**	**N**	**%**	**N**	**%**	
**All Patients**	**1,536,048**	**100.0%**	**1,422,938**	**92.6%**	**113,110**	**7.4%**		**224,804**	**100.0%**	**112,402**	**50.0%**	**112,402**	**50.0%**	
**Age**														
≤20	89,565	5.8%	89,486	6.3%	79	0.1%	<0.001	155	0.1%	76	0.1%	79	0.1%	1.00
21-40	315,593	20.5%	314,815	22.1%	778	0.7%		1,562	0.7%	784	0.7%	778	0.7%	
41-50	233,336	15.2%	230,961	16.2%	2,375	2.1%		4,746	2.1%	2,371	2.1%	2,375	2.1%	
51-60	333,650	21.7%	314,109	22.1%	19,541	17.3%		39,072	17.4%	19,536	17.4%	19,536	17.4%	
61-70	296,182	19.3%	254,286	17.9%	41,896	37.0%		83,664	37.2%	41,834	37.2%	41,830	37.2%	
71-80	169,295	11.0%	136,746	9.6%	32,549	28.8%		64,163	28.5%	32,073	28.5%	32,090	28.5%	
≥81	98,427	6.4%	82,535	5.8%	15,892	14.1%		31,442	14.0%	15,728	14.0%	15,714	14.0%	
**Gender**														
Female	1,109,580	72.2%	1,004,112	70.6%	105,468	93.2%	<0.001	209,510	93.2%	104,743	93.2%	104,767	93.2%	0.84
Male	426,468	27.8%	418,826	29.4%	7,642	6.8%		15,294	6.8%	7,659	6.8%	7,635	6.8%	
**Region**														
Midwest	335,508	21.8%	315,179	22.1%	20,329	18.0%	<0.001	40,548	18.0%	20,274	18.0%	20,274	18.0%	1.00
Northeast	383,023	24.9%	356,184	25.0%	26,839	23.7%		53,590	23.8%	26,795	23.8%	26,795	23.8%	
South	596,029	38.8%	552,754	38.8%	43,275	38.3%		85,440	38.0%	42,720	38.0%	42,720	38.0%	
West	221,488	14.4%	198,821	14.0%	22,667	20.0%		45,226	20.1%	22,613	20.1%	22,613	20.1%	
**Insurance**														
Commercial	707,675	46.1%	672,990	47.3%	34,685	30.7%	<0.001	69,354	30.9%	34,675	30.8%	34,679	30.9%	1.00
Dual	54,836	3.6%	44,281	3.1%	10,555	9.3%		19,871	8.8%	9,927	8.8%	9,944	8.8%	
Medicaid	486,446	31.7%	463,857	32.6%	22,589	20.0%		45,057	20.0%	22,537	20.1%	22,520	20.0%	
Medicare	287,091	18.7%	241,810	17.0%	45,281	40.0%		90,522	40.3%	45,263	40.3%	45,259	40.3%	
**PCP Visit 2019**														
No	605,256	39.4%	572,701	40.2%	32,555	28.8%	<0.001	64,959	28.9%	32,483	28.9%	32,476	28.9%	0.97
Yes	930,792	60.6%	850,237	59.8%	80,555	71.2%		159,845	71.1%	79,919	71.1%	79,926	71.1%	
**Continuous Outcomes**														
	**mean**	**SD**	**mean**	**SD**	**mean**	**SD**	**p-value**	**mean**	**SD**	**mean**	**SD**	**mean**	**SD**	**p-value**
**CCI**	0.87	1.60	0.85	1.58	1.06	1.84	<0.001	1.06	1.82	1.05	1.81	1.06	1.83	0.57

BP: bisphosphonate; CCI: Charlson Comorbidity Index; PCP: primary care physician; SD: standard deviation.

**Appendix 2—table 47.** Antidepressant Non-user Cohort (Region=New York State) by BP Use, Patient Characteristics Pre/Post Match of BP Users/Non-users.

	Region=NY Antidepressant Non-users by BP: Unadjusted							Region=NY Antidepressant Non-users by BP: Matched						
	**All**		**BP Non-user**		**BP User**		**p-value**	**All**		**BP Non-user**		**BP User**		**p-value**
	**N**	**%**	**N**	**%**	**N**	**%**		**N**	**%**	**N**	**%**	**N**	**%**	
**All Patients**	**135,516**	**100.0%**	**125,342**	**92.5%**	**10,174**	**7.5%**		**20,182**	**100.0%**	**10,091**	**50.0%**	**10,091**	**50.0%**	
**Age**														
≤20	4,365	3.2%	4,364	3.5%	1	0.0%	<0.001	2	0.0%	1	0.0%	1	0.0%	1.00
21-40	22,832	16.8%	22,799	18.2%	33	0.3%		66	0.3%	33	0.3%	33	0.3%	
41-50	18,483	13.6%	18,350	14.6%	133	1.3%		267	1.3%	134	1.3%	133	1.3%	
51-60	29,481	21.8%	28,038	22.4%	1,443	14.2%		2,879	14.3%	1,440	14.3%	1,439	14.3%	
61-70	27,543	20.3%	24,197	19.3%	3,346	32.9%		6,686	33.1%	3,345	33.1%	3,341	33.1%	
71-80	21,038	15.5%	17,695	14.1%	3,343	32.9%		6,589	32.6%	3,294	32.6%	3,295	32.7%	
≥81	11,774	8.7%	9,899	7.9%	1,875	18.4%		3,693	18.3%	1,844	18.3%	1,849	18.3%	
**Gender**														
Female	96,468	71.2%	86,945	69.4%	9,523	93.6%	<0.001	18,892	93.6%	9,446	93.6%	9,446	93.6%	1.00
Male	39,048	28.8%	38,397	30.6%	651	6.4%		1,290	6.4%	645	6.4%	645	6.4%	
**Insurance**														
Commercial	51,829	38.2%	50,405	40.2%	1,424	14.0%	<0.001	2,848	14.1%	1,425	14.1%	1,423	14.1%	1.00
Dual	1,591	1.2%	1,210	1.0%	381	3.7%		690	3.4%	345	3.4%	345	3.4%	
Medicaid	38,569	28.5%	36,303	29.0%	2,266	22.3%		4,449	22.0%	2,226	22.1%	2,223	22.0%	
Medicare	43,527	32.1%	37,424	29.9%	6,103	60.0%		12,195	60.4%	6,095	60.4%	6,100	60.4%	
**PCP Visit 2019**														
No	53,397	39.4%	50,515	40.3%	2,882	28.3%	<0.001	5,723	28.4%	2,863	28.4%	2,860	28.3%	0.96
Yes	82,119	60.6%	74,827	59.7%	7,292	71.7%		14,459	71.6%	7,228	71.6%	7,231	71.7%	
**Continuous Outcomes**														
	**mean**	**SD**	**mean**	**SD**	**mean**	**SD**	**p-value**	**mean**	**SD**	**mean**	**SD**	**mean**	**SD**	**p-value**
**CCI**	0.96	1.68	0.95	1.66	1.13	1.81	<0.001	1.11	1.77	1.11	1.76	1.12	1.78	0.78

BP: bisphosphonate; CCI: Charlson Comorbidity Index; PCP: primary care physician; SD: standard deviation.

## Appendix 3

### Post-hoc analysis on the impact of censoring due to death

#### Background

Following completion of all core study analyses, an additional post-hoc investigation was performed to assess whether censoring bias due to patient death could impact our current findings of a decrease in the odds of COVID-19 outcomes seen amongst BP users. Typically, it is very difficult to perform assessments on this type of bias due to the fact that insurance claims databases in the United States do not include this information. Some claims database providers, including Komodo Health, do have the capability to ‘link’ their de-identified claims data with external sources on decedent enrolees, but at the time of study initiation and data extraction there were enhanced HIPAA constraints associated with claims datasets that included COVID-identifying diagnosis/treatment codes due to the heightened risk of patient re-identification due to the then lower prevalence and high visibility associated for patients with COVID-19. Eventually the increased prevalence of COVID-19 reduced the HIPAA concerns on working with claims data that include COVID-19-identifiers, and in support of this analysis and the potentially significant public health implications of our findings, Komodo Health linked their COVID-identifiable dataset with mortality data sources that account for roughly 80–85% of available death records. In conjunction with Komodo Health, queries on this mortality-linked COVID-19-identifiable dataset were performed to determine whether bias caused by patient censoring due to death could have impacted the validity and/or reliability of our current findings

#### Methodological concerns of patient censoring due to death

The single motivating factor for initiation of this post-hoc analysis was the fact that the decrease in odds of COVID-19 outcomes among BP users in this study was found to be statistically significant, large in magnitude, and robust across almost all analysis variations performed. The exhaustive use of methodological techniques to control for unmeasured confounding and/or outside sources of bias employed in this current study were undertaken not in search of statistical significance, but in search of non-significance. This was undertaken because the consistency seen in statistical significance, in addition to the magnitude of the decrease in the odds of our outcomes of interest, are typically not seen to this degree. As such, the next logical step after exhausting all methodological techniques is to search for other sources that could induce a large-enough bias on the underlying patient population itself, such as censoring of the target study cohort, that could drastically alter the typical composition of the overall sample and thus impact the reliability and validity of outcomes measured.

The high rate of death associated with COVID-19 infection, which was even worse during the early months of the pandemic, represents such an instance where outside influences could impact the underlying data, and as such, the validity of research performed on that data. The primary concern is whether patients who have died are censored from the analytical sample due to the application of one of the most fundamental inclusion/exclusion criteria used in claims-based research, the requirement for continuous insurance eligibility over the entire study period that is needed so that healthcare resource utilization events from all subjects are captured and available in the data for analysis. If in our current sample, a larger number of BP users died after contracting COVID-19 and were censored due to insurance eligibility, and a lower number of BP non-users survived and thus met the insurance eligibility criteria, then the remaining study sample would be comprised of healthier-looking BP users and a higher number of BP non-users with COVID-19 related healthcare services.

The potential for such a censoring bias in this current study sample, and the impact of that bias on the magnitude and statistical significance of our core study findings, was assessed in this post-hoc analysis by: (1) adjusting eligibility criteria to prevent the censoring of patients that may have died during the first half of 2020; (2) replicating key exposure (BP-use, use of other non-BP bone health medications) and outcomes (COVID-19 diagnosis) in this expanded sample that aligns with the core study methods; (3) analysing the impact on study findings that would result from the retention and inclusion of deceased-patient observations in the core study sample on the odds of COVID-19 diagnosis; and (4) calculating the number of missing patient observations censored due to death that would be required to reach a statistically non-significant difference in the odds of COVID-19.

### Post-Hoc analysis

#### Methods

##### Cohort definition

- Continuous insurance eligibility 1/1/2019-12/31/2019; used to ensure that any censoring due to death occurs during the observation period of 1/1/2020-6/30/2020
- BP users compared to BP non-users to produce a cohort comparison similar to the primary analysis cohort
- BP users compared to users of non-BP anti-resorptive bone health medications to produce a cohort comparison similar to the “*Bone-Rx*” active comparator analysis

##### Exposures of interest

- Patients were assigned into the BP user cohort if they had any claim 1/1/2019-2/29/2020 for one of the following: alendronate, alendronic acid, etidronate, ibandronate, ibandronic acid, pamidronate, risedronate, and zoledronic acid; for the cohort comparison of all osteoporosis medication users BP users were further restricted to those that had no claims for a non-BP anti-resorptive bone health medication 1/1/2019-2/29/2020.
- Patients were assigned into the non-BP anti-resorptive bone health medication user cohort if: (1) they had any claim 1/1/2019-2/29/2020 for one of the following: denosumab, calcitonin, raloxifene, romosozumab-aqqg, teriparatide, abaloparatide, or bazedoxifene; and (2) they had no BP claims

##### Outcomes / endpoints

- Patients were assigned into the COVID-19 diagnosis cohort based on any medical service claim with an ICD-10 diagnosis code of U07.1 occurring 1/1/2200-6/30/2020
- Patients with a date-of-death between 1/1/2020-6/30/2020 were classified into the deceased cohort

##### Statistical analysis

- Chi-square testing was used to assess whether statistically significant differences exist between BP users and BP non-users in the unadjusted odds of having any COVID-19 diagnosis during the first half of 2020 among cohorts that approximate the primary analysis and “*Bone-Rx*” study cohorts for the following:

1. Among all patient-observations with a COVID-19 diagnosis to assess the potential ‘true’ comparison that would occur
2. With deceased patient-observations that had a known COVID-19 diagnosis removed prior to testing to replicate findings that would occur if these observations were censored
3. When making the assumption that all patients who died during this period died due to COVID-19, and thus should be classified as having a COVID-19 diagnosis

An additional analysis was performed on the last variation modelled (assuming all patients died due to COVID-19) to determine the additional BP user patient observations that would be needed to be classified as having had a COVID-19 diagnosis to yield a similar distribution of COVID-19 diagnosis (yes/no) as was seen in the BP non-user cohort to yield an odds ratio ~1.0

Finally, the impact on odds ratio testing results comparing BP users to BP non-users was modelled based on the additional number of BP users needed to be classified as having been diagnosed with COVID-19 to reach statistical non-significance

#### Results

##### Patient count distribution

Among the full sample a decreased rate of COVID-19 among BP users compared to BP non-users was seen in both the full sample population (1.2% vs 4.7%) as well as when restricted to users of non-BP anti-resorptive bone health medications (1.2% versus 4.3%) (Appendix 3—table 1)

##### Unadjusted Chi-square comparison inclusive of deceased patients

The decrease in the odds of any COVID-19 diagnosis amongst BP users compared to BP non-users was found to be robust in both the full (OR = 0.24) and “Bone-Rx” (OR = 0.35) comparisons when including deceased patients with a known COVID-19 diagnosis (Appendix 3—table 2)

##### Unadjusted Chi-square comparison with deceased patients removed

The decrease in the odds of any COVID-19 diagnosis amongst BP users compared to BP non-users was found to be robust in both the full (OR = 0.23) and “Bone-Rx” (OR = 0.26) comparisons when removing deceased patients with a known COVID-19 diagnosis (Appendix 3—table 3)

##### Unadjusted Chi-square comparison assuming all deceased patients had COVID-19

- The decrease in the odds of any COVID-19 diagnosis amongst BP users compared to BP non-users was found to be robust in both the full (OR = 0.39) and “*Bone-Rx*” (OR = 0.29) comparisons when assuming that all deceased patients had a COVID-19 diagnosis (Appendix 3—table 4)
- Among this final analysis that assumes all deceased patients had a diagnosis of COVID-19, the percentage of BP non-users with an assumed COVID-19 diagnosis was 5.5% and 7.2% for the full and OPRX comparisons, respectively.
- These proportions were then used to estimate the number of additional BP users with a COVID-19 diagnosis that would be needed to have the same distribution and thus an odds ratio ~1.0 (Appendix 3—table 5)
- It would require an additional 22,235 (37,095-14,860) BP-user patient observations from the full cohort comparison to be classified as having a COVID-19 diagnosis to have an equivalent odds of being diagnosed with COVID-19 as was seen among the BP non-user cohort
- It would require an additional 32,598 (46,637-14,039) BP-user patient observations from the “*Bone-Rx*” cohort comparison to be classified as having a COVID-19 diagnosis to have an equivalent odds of being diagnosed with COVID-19 as was seen among the BP non-user cohort
- In the full (all observations) comparison, the minimum number of additional BP users classified as having a COVID-19 diagnosis needed to reach statistical non-significance for the calculated unadjusted odds ratio was 21,860 (Appendix 3—figure 1)
- In the “*Bone-Rx*” comparison, the minimum number of additional BP users classified as having a COVID-19 diagnosis needed to reach statistical non-significance for the calculated unadjusted odds ratio was 31,360 (Appendix 3—figure 2)

**Appendix 3—table 1.** Patient Count Distribution Inclusive of Deceased Enrolees.

	All Observations		All Bone Health Rx Users(“Bone-Rx”)	
	BP Users	BP Non-users	BP Users	BP Non-users
Total (N)	672,913	10,978,373	645,118	75,195
Deceased (N) [*any reason*]	7,364	101,282	6,922	2,450
COVID-19 Dx (N)	7,927	519,387	7,527	3,201
COVID-19 Dx (%)	1.2%	4.7%	1.2%	4.3%
COVID-19 Dx & Deceased (N)	431	15,470	410	215
COVID-19 Dx & Deceased (%)	5.4%	3.0%	5.4%	6.7%

Dx: diagnosis.

**Appendix 3—table 2.** Unadjusted Chi-Square Comparison Inclusive of Deceased Patients.

	All Observations (with deceased)		“Bone-Rx” Observations (with deceased)	
	COVID-19 Dx	No COVID-19 Dx	COVID-19 Dx	No COVID-19 Dx
BP users	7,927	664,986	7,527	637,591
BP Non-users	519,387	10,458,986	2,450	71,994
	Odds Ratio	0.24	Odds Ratio	0.35
	95 % CI:	0.2347 to 0.2455	95 % CI:	0.3312 to 0.3633
	p-value	P < 0.0001	p-value	P < 0.0001

BP: bisphosphonate; CI: confidence interval; Dx: diagnosis.

**Appendix 3—table 3.** Unadjusted Chi-Square Comparison with Deceased Patients Removed.

	All Observations (without deceased)		“Bone-Rx” Observations (without deceased)	
	COVID-19 Dx	No COVID-19 Dx	COVID-19 Dx	No COVID-19 Dx
BP users	7,496	657,622	7,117	630,669
BP Non-users	503,917	10,357,704	2,986	69,544
	Odds Ratio	0.23	Odds Ratio	0.26
	95 % CI:	0.2290–0.2397	95 % CI:	0.2516–0.2745
	p-value	*P*<0.0001	p-value	*P*<0.0001

BP: bisphosphonate; CI: confidence interval; Dx: diagnosis.

**Appendix 3—table 4.** Unadjusted Chi-Square Comparison Assuming all Deceased Patients had COVID-19.

	All Observations (assume deceased = COVID-19)		“Bone-Rx” Observations (assume deceased = COVID-19)	
	COVID-19 Dx	No COVID-19 Dx	COVID-19 Dx	No COVID-19 Dx
BP users	14,860	658,053	14,039	631,079
BP Non-users	605,199	10,373,174	5,436	69,759
	Odds Ratio	0.39	Odds Ratio	0.29
	95 % CI:	0.3807–0.3935	95 % CI:	0.2764–0.2948
	p-value	*P*<0.0001	p-value	*P*<0.0001

BP: bisphosphonate; CI: confidence interval; Dx: diagnosis.

**Appendix 3—table 5.** Unadjusted Chi-Square Comparison to Yield Odds Ratio = 1.00 (no difference).

	All Observations (assume deceased = COVID-19)		“Bone-Rx” Observations (assume deceased = COVID-19)	
	COVID-19 Dx	No COVID-19 Dx	COVID-19 Dx	No COVID-19 Dx
BP users	37,095	635,818	46,637	598,481
BP Non-users	605,199	10,373,174	5,436	69,759
	Odds Ratio	1.00	Odds Ratio	1.00
	95 % CI:	0.9893–1.0108	95 % CI:	0.9713–1.0296
	p-value	*P*=0.9987	p-value	*P*=0.9999

BP: bisphosphonate; CI: confidence interval; Dx: diagnosis.

## References

[bib1] Abdullah MI, Abed MN, Richardson A (2017). Inhibition of the mevalonate pathway augments the activity of pitavastatin against ovarian cancer cells. Scientific Reports.

[bib2] Atmaca A, Demirci I, Haymana C, Tasci I, Sahin I, Cakal E, Ata N, Dagdelen S, Salman S, Emral R, Sahin M, Celik O, Demir T, Ertugrul D, Unluturk U, Caglayan M, Satman I, Sonmez A (2022). No association of anti-osteoporosis drugs with COVID-19-related outcomes in women: a nationwide cohort study. Osteoporosis International.

[bib3] Azar KMJ, Shen Z, Romanelli RJ, Lockhart SH, Smits K, Robinson S, Brown S, Pressman AR (2020). Disparities In Outcomes Among COVID-19 Patients In A Large Health Care System In California. Health Affairs.

[bib4] Black DM, Geiger EJ, Eastell R, Vittinghoff E, Li BH, Ryan DS, Dell RM, Adams AL (2020). Atypical femur fracture risk versus fragility fracture prevention with bisphosphonates. The New England Journal of Medicine.

[bib5] Blanch-Rubió J, Soldevila-Domenech N, Tío L, Llorente-Onaindia J, Ciria-Recasens M, Polino L, Gurt A, de la Torre R, Maldonado R, Monfort J, the Covidmar Study Group (2020). Influence of anti-osteoporosis treatments on the incidence of COVID-19 in patients with non-inflammatory rheumatic conditions. Aging.

[bib6] Brufsky A, Marti JLG, Nasrazadani A, Lotze MT (2020). Boning up: amino-bisphophonates as immunostimulants and endosomal disruptors of dendritic cell in SARS-CoV-2 infection. Journal of Translational Medicine.

[bib7] CDC (2021a). United States COVID-19 Cases and Death by State Over Time. https://www.cdc.gov/nchs/pressroom/sosmap/covid19_mortality_final/COVID19.htm.

[bib8] CDC (2021b). Risk for COVID-19 Infection, Hospitalization, and Death By Race/Ethnicity. https://stacks.cdc.gov/view/cdc/105453.

[bib9] Chadwick JW, Tenenbaum HC, Sun CX, Wood RE, Glogauer M (2020). The effect of pamidronate delivery in bisphosphonate-naïve patients on neutrophil chemotaxis and oxidative burst. Scientific Reports.

[bib10] Chen J, Rizzo JA (2008). Racial and ethnic disparities in antidepressant drug use. The Journal of Mental Health Policy and Economics.

[bib11] Colón-Emeric CS, Mesenbrink P, Lyles KW, Pieper CF, Boonen S, Delmas P, Eriksen EF, Magaziner J (2010). Potential mediators of the mortality reduction with zoledronic acid after hip fracture. Journal of Bone and Mineral Research.

[bib12] Cremers S, Drake MT, Ebetino FH, Bilezikian JP, Russell RGG (2019). Pharmacology of bisphosphonates. British Journal of Clinical Pharmacology.

[bib13] Curtis JR, Larson JC, Delzell E, Brookhart MA, Cadarette SM, Chlebowski R, Judd S, Safford M, Solomon DH, Lacroix AZ (2011). Placebo adherence, clinical outcomes, and mortality in the women’s health initiative randomized hormone therapy trials. Medical Care.

[bib14] Degli Esposti L, Perrone V, Sangiorgi D, Andretta M, Bartolini F, Cavaliere A, Ciaccia A, Dell’orco S, Grego S, Salzano S, Ubertazzo L, Vercellone A, Gatti D, Fassio A, Viapiana O, Rossini M, Adami G (2021). The Use of Oral Amino-Bisphosphonates and Coronavirus Disease 2019 (COVID-19) Outcomes. Journal of Bone and Mineral Research.

[bib15] Dhesy-Thind S, Fletcher GG, Blanchette PS, Clemons MJ, Dillmon MS, Frank ES, Gandhi S, Gupta R, Mates M, Moy B, Vandenberg T, Van Poznak CH (2017). Use of adjuvant bisphosphonates and other bone-modifying agents in breast cancer: A Cancer care ontario and american society of clinical oncology clinical practice guideline. Journal of Clinical Oncology.

[bib16] Dormuth CR, Patrick AR, Shrank WH, Wright JM, Glynn RJ, Sutherland J, Brookhart MA (2009). Statin adherence and risk of accidents: a cautionary tale. Circulation.

[bib17] Emanuel EJ, Persad G, Upshur R, Thome B, Parker M, Glickman A, Zhang C, Boyle C, Smith M, Phillips JP (2020). Fair Allocation of Scarce Medical Resources in the Time of Covid-19. The New England Journal of Medicine.

[bib18] Escobar GJ, Adams AS, Liu VX, Soltesz L, Chen YFI, Parodi SM, Ray GT, Myers LC, Ramaprasad CM, Dlott R, Lee C (2021). Racial Disparities in COVID-19 Testing and Outcomes. Annals of Internal Medicine.

[bib19] Favot CL, Forster C, Glogauer M (2013). The effect of bisphosphonate therapy on neutrophil function: a potential biomarker. International Journal of Oral and Maxillofacial Surgery.

[bib20] George S, Weber DR, Kaplan P, Hummel K, Monk HM, Levine MA (2015). Short-term safety of zoledronic acid in young patients with bone disorders: An extensive institutional experience. The Journal of Clinical Endocrinology and Metabolism.

[bib21] Gu A, Yue Y, Desai RP, Argulian E (2017). Racial and Ethnic Differences in Antihypertensive Medication Use and Blood Pressure Control Among US Adults With Hypertension. Circulation.

[bib22] Hermesh T, Moltedo B, Moran TM, López CB (2010). Antiviral instruction of bone marrow leukocytes during respiratory viral infections. Cell Host & Microbe.

[bib23] Hewitt RE, Lissina A, Green AE, Slay ES, Price DA, Sewell AK (2005). The bisphosphonate acute phase response: rapid and copious production of proinflammatory cytokines by peripheral blood gd T cells in response to aminobisphosphonates is inhibited by statins. Clinical and Experimental Immunology.

[bib24] Hoertel N, Sánchez-Rico M, Vernet R, Beeker N, Jannot A-S, Neuraz A, Salamanca E, Paris N, Daniel C, Gramfort A, Lemaitre G, Bernaux M, Bellamine A, Lemogne C, Airagnes G, Burgun A, Limosin F, AP-HP / Universities / INSERM COVID-19 Research Collaboration and AP-HP COVID CDR Initiative (2021). Association between antidepressant use and reduced risk of intubation or death in hospitalized patients with COVID-19: results from an observational study. Molecular Psychiatry.

[bib25] Israel A, Schäffer AA, Cicurel A, Cheng K, Sinha S, Schiff E, Feldhamer I, Tal A, Lavie G, Ruppin E (2021). Identification of drugs associated with reduced severity of COVID-19 - a case-control study in a large population. eLife.

[bib26] Istvan ES, Deisenhofer J (2001). Structural mechanism for statin inhibition of HMG-CoA reductase. Science.

[bib27] Jacobson M, Chang TY, Shah M, Pramanik R, Shah SB (2021). Racial and Ethnic Disparities in SARS-CoV-2 Testing and COVID-19 Outcomes in a Medicaid Managed Care Cohort. American Journal of Preventive Medicine.

[bib28] Karmakar M, Lantz PM, Tipirneni R (2021). Association of Social and Demographic Factors With COVID-19 Incidence and Death Rates in the US. JAMA Network Open.

[bib29] Kaufman HW, Niles JK, Nash DB (2021). Disparities in SARS-CoV-2 Positivity Rates: Associations with Race and Ethnicity. Population Health Management.

[bib30] Kavanagh KL, Guo K, Dunford JE, Wu X, Knapp S, Ebetino FH, Rogers MJ, Russell RGG, Oppermann U (2006). The molecular mechanism of nitrogen-containing bisphosphonates as antiosteoporosis drugs. PNAS.

[bib31] Kluberg SA, Hou L, Dutcher SK, Billings M, Kit B, Toh S, Dublin S, Haynes K, Kline A, Maiyani M, Pawloski PA, Watson ES, Cocoros NM (2022). Validation of diagnosis codes to identify hospitalized COVID-19 patients in health care claims data. Pharmacoepidemiology and Drug Safety.

[bib32] Kuiper JWP, Forster C, Sun C, Peel S, Glogauer M (2012). Zoledronate and pamidronate depress neutrophil functions and survival in mice. British Journal of Pharmacology.

[bib33] Ladova K, Vlcek J, Vytrisalova M, Maly J (2014). Healthy adherer effect - the pitfall in the interpretation of the effect of medication adherence on health outcomes. Journal of Evaluation in Clinical Practice.

[bib34] Lee P, Ng C, Slattery A, Nair P, Eisman JA, Center JR (2016). Preadmission bisphosphonate and mortality in critically ill patients. The Journal of Clinical Endocrinology and Metabolism.

[bib35] Liu H, Wang SH, Chen SC, Chen CY, Lo JL, Lin TM (2016). Immune modulation of CD4+CD25+ regulatory T cells by zoledronic acid. BMC Immunology.

[bib36] Lohia P, Kapur S, Benjaram S, Mir T (2021). Association between antecedent statin use and severe disease outcomes in COVID-19: A retrospective study with propensity score matching. Journal of Clinical Lipidology.

[bib37] Mazo IB, Honczarenko M, Leung H, Cavanagh LL, Bonasio R, Weninger W, Engelke K, Xia L, McEver RP, Koni PA, Silberstein LE, von Andrian UH (2005). Bone marrow is a major reservoir and site of recruitment for central memory CD8+ T cells. Immunity.

[bib38] Meizlish ML, Pine AB, Bishai JD, Goshua G, Nadelmann ER, Simonov M, Chang CH, Zhang H, Shallow M, Bahel P, Owusu K, Yamamoto Y, Arora T, Atri DS, Patel A, Gbyli R, Kwan J, Won CH, Dela Cruz C, Price C, Koff J, King BA, Rinder HM, Wilson FP, Hwa J, Halene S, Damsky W, van Dijk D, Lee AI, Chun HJ (2021). A neutrophil activation signature predicts critical illness and mortality in COVID-19. Blood Advances.

[bib39] Migliorati CA, Siegel MA, Elting LS (2006). Bisphosphonate-associated osteonecrosis: a long-term complication of bisphosphonate treatment. The Lancet. Oncology.

[bib40] Muñoz-Price LS, Nattinger AB, Rivera F, Hanson R, Gmehlin CG, Perez A, Singh S, Buchan BW, Ledeboer NA, Pezzin LE (2020). Racial Disparities in Incidence and Outcomes Among Patients With COVID-19. JAMA Network Open.

[bib41] Nada MH, Wang H, Workalemahu G, Tanaka Y, Morita CT (2017). Enhancing adoptive cancer immunotherapy with Vγ2Vδ2 T cells through pulse zoledronate stimulation. Journal for Immunotherapy of Cancer.

[bib42] Nau C, Bruxvoort K, Navarro RA, Chevez SG, Hogan TA, Ironside KR, Ludwig SM, Ngo-Metzger Q, Mourra NR, Young DR, Sangha N, Turner BP, Li IX, Padilla A, Chen A, Hong V, Yau V, Tartof S (2021). COVID-19 Inequities Across Multiple Racial and Ethnic Groups: Results From an Integrated Health Care Organization. Annals of Internal Medicine.

[bib43] No authors listed (2021). QuickStats: Percentage* of Adults Aged ≥50 Years with Osteoporosis,† by Race and Hispanic 1285 Origin§ — United States, 2017–2018. MMWR Morbidity and Mortality Weekly Report.

[bib44] Parsons LS (2001). Reducing bias in a propensity score matched-pair sample using greedy matching techniques.

[bib45] Petersen ML, Porter KE, Gruber S, Wang Y, van der Laan MJ (2012). Diagnosing and responding to violations in the positivity assumption. Statistical Methods in Medical Research.

[bib46] Poccia F, Agrati C, Castilletti C, Bordi L, Gioia C, Horejsh D, Ippolito G, Chan PKS, Hui DSC, Sung JJY, Capobianchi MR, Malkovsky M (2006). Anti-severe acute respiratory syndrome coronavirus immune responses: the role played by V gamma 9V delta 2 T cells. The Journal of Infectious Diseases.

[bib47] Price-Haywood EG, Burton J, Fort D, Seoane L (2020). Hospitalization and Mortality among Black Patients and White Patients with Covid-19. The New England Journal of Medicine.

[bib48] Quan H, Sundararajan V, Halfon P, Fong A, Burnand B, Luthi JC, Saunders LD, Beck CA, Feasby TE, Ghali WA (2005). Coding algorithms for defining comorbidities in ICD-9-CM and ICD-10 administrative data. Medical Care.

[bib49] Reid IR, Horne AM, Mihov B, Stewart A, Bastin S, Gamble GD (2021). Effect of zoledronate on lower respiratory infections in older women: secondary analysis of a randomized controlled trial. Calcified Tissue International.

[bib50] Reitsma MB, Claypool AL, Vargo J, Shete PB, McCorvie R, Wheeler WH, Rocha DA, Myers JF, Murray EL, Bregman B, Dominguez DM, Nguyen AD, Porse C, Fritz CL, Jain S, Watt JP, Salomon JA, Goldhaber-Fiebert JD (2021). Racial/Ethnic Disparities In COVID-19 Exposure Risk, Testing, And Cases At The Subcounty Level In California. Health Affairs.

[bib51] Reusch N, De Domenico E, Bonaguro L, Schulte-Schrepping J, Baßler K, Schultze JL, Aschenbrenner AC (2021). Neutrophils in COVID-19. Frontiers in Immunology.

[bib52] Ribot JC, Lopes N, Silva-Santos B (2021). γδ T cells in tissue physiology and surveillance. Nature Reviews. Immunology.

[bib53] Roelofs AJ, Coxon FP, Ebetino FH, Lundy MW, Henneman ZJ, Nancollas GH, Sun S, Blazewska KM, Bala JLF, Kashemirov BA, Khalid AB, McKenna CE, Rogers MJ (2010a). Fluorescent risedronate analogues reveal bisphosphonate uptake by bone marrow monocytes and localization around osteocytes in vivo. Journal of Bone and Mineral Research.

[bib54] Roelofs AJ, Thompson K, Ebetino FH, Rogers MJ, Coxon FP (2010b). Bisphosphonates: molecular mechanisms of action and effects on bone cells, monocytes and macrophages. Current Pharmaceutical Design.

[bib55] Rogers TL, Holen I (2011). Tumour macrophages as potential targets of bisphosphonates. Journal of Translational Medicine.

[bib56] Rogers TN, Rogers CR, VanSant-Webb E, Gu LY, Yan B, Qeadan F (2020). Racial Disparities in COVID-19 Mortality Among Essential Workers in the United States. World Medical & Health Policy.

[bib57] Rosenthal N, Cao Z, Gundrum J, Sianis J, Safo S (2020). Risk Factors Associated With In-Hospital Mortality in a US National Sample of Patients With COVID-19. JAMA Network Open.

[bib58] Rubin-Miller L (2020). COVID-19 Racial Disparities in Testing, Infection, Hospitalization, and Death: Analysis of Epic Patient Data. https://www.kff.org/coronavirus-covid-19/issue-brief/covid-19-racial-disparities-testing-infection-hospitalization-death-analysis-epic-patient-data.

[bib59] Russell RGG (2007). Bisphosphonates: mode of action and pharmacology. Pediatrics.

[bib60] Russell RGG, Watts NB, Ebetino FH, Rogers MJ (2008). Mechanisms of action of bisphosphonates: similarities and differences and their potential influence on clinical efficacy. Osteoporosis International.

[bib61] Saita Y, Ishijima M, Kaneko K (2015). Atypical femoral fractures and bisphosphonate use: current evidence and clinical implications. Therapeutic Advances in Chronic Disease.

[bib62] Salami JA, Warraich H, Valero-Elizondo J, Spatz ES, Desai NR, Rana JS, Virani SS, Blankstein R, Khera A, Blaha MJ, Blumenthal RS, Lloyd-Jones D, Nasir K (2017). National Trends in Statin Use and Expenditures in the US Adult Population From 2002 to 2013: Insights From the Medical Expenditure Panel Survey. JAMA Cardiology.

[bib63] Sarhan D, Leijonhufvud C, Murray S, Witt K, Seitz C, Wallerius M, Xie H, Ullén A, Harmenberg U, Lidbrink E, Rolny C, Andersson J, Lundqvist A (2017). Zoledronic acid inhibits NFAT and IL-2 signaling pathways in regulatory T cells and diminishes their suppressive function in patients with metastatic cancer. Oncoimmunology.

[bib64] Sbrocchi AM, Forget S, Laforte D, Azouz EM, Rodd C (2010). Zoledronic acid for the treatment of osteopenia in pediatric Crohn’s disease. Pediatrics International.

[bib65] Selden TM, Berdahl TA (2020). COVID-19 And Racial/Ethnic Disparities In Health Risk, Employment, And Household Composition. Health Affairs.

[bib66] Sing CW, Kiel DP, Hubbard RB, Lau WC, Li GH, Kung AW, Wong IC, Cheung CL (2020). Nitrogen-containing bisphosphonates are associated with reduced risk of pneumonia in patients with hip fracture. Journal of Bone and Mineral Research.

[bib67] Sultana J, Crisafulli S, Gabbay F, Lynn E, Shakir S, Trifirò G (2020). Challenges for Drug Repurposing in the COVID-19 Pandemic Era. Frontiers in Pharmacology.

[bib68] Suresh E, Pazianas M, Abrahamsen B (2014). Safety issues with bisphosphonate therapy for osteoporosis. Rheumatology.

[bib69] Tonti E, Jiménez de Oya N, Galliverti G, Moseman EA, Di Lucia P, Amabile A, Sammicheli S, De Giovanni M, Sironi L, Chevrier N, Sitia G, Gennari L, Guidotti LG, von Andrian UH, Iannacone M (2013). Bisphosphonates target B cells to enhance humoral immune responses. Cell Reports.

[bib70] Tu W, Zheng J, Liu Y, Sia SF, Liu M, Qin G, Ng IHY, Xiang Z, Lam KT, Peiris JSM, Lau YL (2011). The aminobisphosphonate pamidronate controls influenza pathogenesis by expanding a gammadelta T cell population in humanized mice. The Journal of Experimental Medicine.

[bib71] United States Census Bureau (2019). QuickFacts California - Race and Hispanic Origin. United States Census Bureau.

[bib72] US Bureau of Labor Statistics (2019). BLS Reports October 2019: Labor force characteristics by race and ethnicity, 2018. United States Department of Labor.

[bib73] Wang H, Sarikonda G, Puan KJ, Tanaka Y, Feng J, Giner JL, Cao R, Mönkkönen J, Oldfield E, Morita CT (2011). Indirect stimulation of human Vγ2Vδ2 T cells through alterations in isoprenoid metabolism. Journal of Immunology.

[bib74] Wolf AM, Rumpold H, Tilg H, Gastl G, Gunsilius E, Wolf D (2006). The effect of zoledronic acid on the function and differentiation of myeloid cells. Haematologica.

[bib75] Xia Y, Xie Y, Yu Z, Xiao H, Jiang G, Zhou X, Yang Y, Li X, Zhao M, Li L, Zheng M, Han S, Zong Z, Meng X, Deng H, Ye H, Fa Y, Wu H, Oldfield E, Hu X, Liu W, Shi Y, Zhang Y (2018). The mevalonate pathway is a druggable target for vaccine adjuvant discovery. Cell.

[bib76] Zhang XJ, Qin JJ, Cheng X, Shen L, Zhao YC, Yuan Y, Lei F, Chen MM, Yang H, Bai L, Song X, Lin L, Xia M, Zhou F, Zhou J, She ZG, Zhu L, Ma X, Xu Q, Ye P, Chen G, Liu L, Mao W, Yan Y, Xiao B, Lu Z, Peng G, Liu M, Yang J, Yang L, Zhang C, Lu H, Xia X, Wang D, Liao X, Wei X, Zhang BH, Zhang X, Yang J, Zhao GN, Zhang P, Liu PP, Loomba R, Ji YX, Xia J, Wang Y, Cai J, Guo J, Li H (2020). In-Hospital Use of Statins Is Associated with a Reduced Risk of Mortality among Individuals with COVID-19. Cell Metabolism.

[bib77] Zhao E, Xu H, Wang L, Kryczek I, Wu K, Hu Y, Wang G, Zou W (2012). Bone marrow and the control of immunity. Cellular & Molecular Immunology.

[bib78] Zheng J, Wu WL, Liu Y, Xiang Z, Liu M, Chan KH, Lau SY, Lam KT, To KKW, Chan JFW, Li L, Chen H, Lau YL, Yuen KY, Tu W (2015). The Therapeutic Effect of Pamidronate on Lethal Avian Influenza A H7N9 Virus Infected Humanized Mice. PLOS ONE.

[bib79] Zimniak M, Kirschner L, Hilpert H, Geiger N, Danov O, Oberwinkler H, Steinke M, Sewald K, Seibel J, Bodem J (2021). The serotonin reuptake inhibitor Fluoxetine inhibits SARS-CoV-2 in human lung tissue. Scientific Reports.

